# LARP4B promotes hepatocellular carcinoma progression and impairs sorafenib efficacy by activating SPINK1-mediated EGFR pathway

**DOI:** 10.1038/s41420-024-01985-6

**Published:** 2024-05-01

**Authors:** Chuanxu Wang, Rui Dong, Feicheng Yang, Lu Zheng, Yingling Liu, Yue Yan, Mengjie Zhang, Bing Ni, Jing Li

**Affiliations:** 1 Department of Hepatobiliary Surgery, Xinqiao Hospital, Third Military Medical University (Army Medical University), Chongqing, China; 2 Department of Pathophysiology, Third Military Medical University (Army Medical University), Chongqing, China; 3 Chongqing International Institute for Immunology, Chongqing, China; 4 Department of Pathology, The First Affiliated Hospital of Hunan Normal University (Hunan Provincial People’s Hospital), Changsha, China

**Keywords:** Cancer epidemiology, RNA modification

## Abstract

La-related proteins (LARPs) regulate gene expression by binding to RNAs and exhibit critical effects on disease progression, including tumors. However, the role of LARP4B and its underlying mechanisms in the progression of hepatocellular carcinoma (HCC) remain largely unclear. In this study, we found that LARP4B expression is upregulated and correlates with poor prognosis in patients with HCC. Gain- and loss-of-function assays showed that LARP4B promotes stemness, proliferation, metastasis, and angiogenesis in vitro and in vivo. Furthermore, LARP4B inhibition enhances the antitumor effects of sorafenib and blocks the metastasis-enhancing effects of low sorafenib concentrations in HCC. Mechanistically, LARP4B expression is upregulated by METTL3-mediated N^6^-methyladenosine (m6A)-IGF2BP3-dependent modification in HCC. RNA- and RNA immunoprecipitation (RIP)- sequencing uncovered that LARP4B upregulates SPINK1 by binding to SPINK1 mRNA via the La motif and maintaining mRNA stability. LARP4B activates the SPINK1-mediated EGFR signaling pathway, which supports stemness, progression and sorafenib resistance in HCC. Additionally, a positive feedback loop with the LARP4B/SPINK1/p-AKT/C/EBP-β axis is responsible for the sorafenib-therapeutic benefit of LARP4B depletion. Overall, this study demonstrated that LARP4B facilitates HCC progression, and LARP4B inhibition provides benefits to sorafenib treatment in HCC, suggesting that LARP4B might be a potential therapeutic target for HCC.

## Background

Hepatocellular carcinoma (HCC) is highly malignant, and exhibits significant biological complexity and heterogeneity. Heterogeneity reflects various genetic or epigenetic changes that endow tumor cells with aggressive tumorigenicity and different sensitivities to treatment, along with diverse molecular signaling features [1, 2]. Intratumoral heterogeneity results from the tumor microenvironment and cancer stem cells (CSCs) [3, 4]. A substantial number of studies have shown that CSCs play an essential role in HCC progression, and molecular signaling pathways related to stemness are unusually activated, particularly through aberrant activation of epidermal growth factor receptor (EGFR) [5, 6]. Amplification and mutation of EGFR can trigger various downstream signaling pathways, leading to uncontrolled cell proliferation, migration, drug resistance, and CSC development [7, 8]. Although some EGFR inhibitors (known as tyrosine kinase inhibitors, TKIs), such as gefitinib, were developed for cancer treatment in HCC, their clinical outcomes are unsatisfactory [9, 10].

RNA-binding proteins (RBPs) interact with RNA via conventional motifs or structural elements to regulate their processing, location, stability, and translation [11, 12]. During the process of malignant transformation, the constantly adjusting dynamic regulatory network of RBPs is adapted to accommodate cancer progression along with the abnormal activation or deactivation of specific oncogenes or tumor suppressor genes [13, 14]. La-related proteins (LARPs) are typical RBPs defined by a highly conserved domain, the La motif, which closely resembles the genuine La protein and combines with RNA recognition motifs to form the La module. Based on their diversity and resemblance to genuine La protein, seven LARPs have been identified: LARP1, 1B, 4, 4B, 6, and 7 [15, 16]. LARPs bind directly to mRNA within conserved La modules that mediate mRNA stability or translation. Emerging evidence has shown that LARPs are dysregulated in cancer and play key roles in cancer progression [16]; however, the exact role of LARP4B in cancer development remains unclear.

In this study, we uncovered the crucial functions of LARP4B in stemness, tumorigenesis, and sorafenib sensitivity and identified the specific mechanisms underlying the oncogenic roles of LARP4B, thus offering a novel target for HCC therapy.

## Results

### LARP4B is highly expressed in HCC and associated with poor prognosis in patients with HCC

**Fig. 1:**
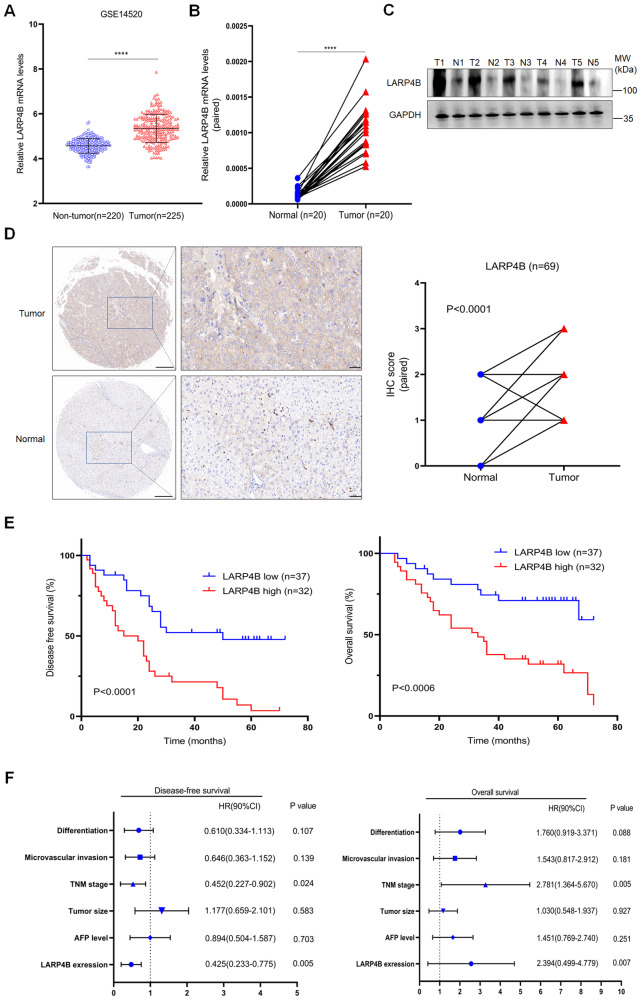
LARP4B is highly expressed in HCC and associated with poor prognosis in patients with HCC. **A** LARP4B mRNA expression in HCC tissues and normal tissues in GEO databases. **B** qPCR of LARP4B in HCC tissues and adjacent normal tissues. **C** Immunoblotting for LARP4B in HCC tissues and adjacent normal tissues. **D** IHC staining of LARP4B (left) and IHC staining scores (right) in a TMA. Scale bars: 200 µm (left), and 50 µm (right). **E** Kaplan–Meier analysis of the DFS (left) and OS (right) of patients with HCC based on the LARP4B expression level. **F** Forest plots based on multivariate analysis of several factors associated with DFS and OS of patients with HCC. All results are presented as the mean ± SD. ****P < 0.0001 by a Mann–Whitney U test for (**A**), and a two-tailed Wilcoxon signed rank test for (**B**, **D**).

First, we analyzed Gene Expression Omnibus (GEO) data to explore the expression profiles of LARPs in HCC. The datasets showed that LARP1 and 4B mRNA were consistently upregulated in HCC tissues compared to normal tissues (Supplementary Fig. S1A–D and Fig. 1A). However, this study focused on exploring LARP4B expression in HCC. In the clinical samples we collected, we found that LARP4B mRNA and protein levels were significantly higher in HCC tissues than in paired normal tissues (Fig. 1B–D). The increased LARP4B expression was associated with TNM stage and AFP level (Supplementary Table S1). Furthermore, Kaplan–Meier analysis showed that HCC patients with high LARP4B expression exhibited shorter disease-free survival (DFS) and overall survival (OS) than those with low LARP4B expression (Fig. 1E). Univariate and multivariate analyses showed that TNM stage and LARP4B expression were independent risk factors for survival in patients with HCC (Fig. 1F). Collectively, these results indicate that the expression of LARP4B at the mRNA and protein levels was upregulated in HCC and correlated with poor prognosis in patients with HCC.

### LARP4B expression is upregulated by METTL3-mediated m6A-IGF2BP3-dependent modification

**Fig. 2:**
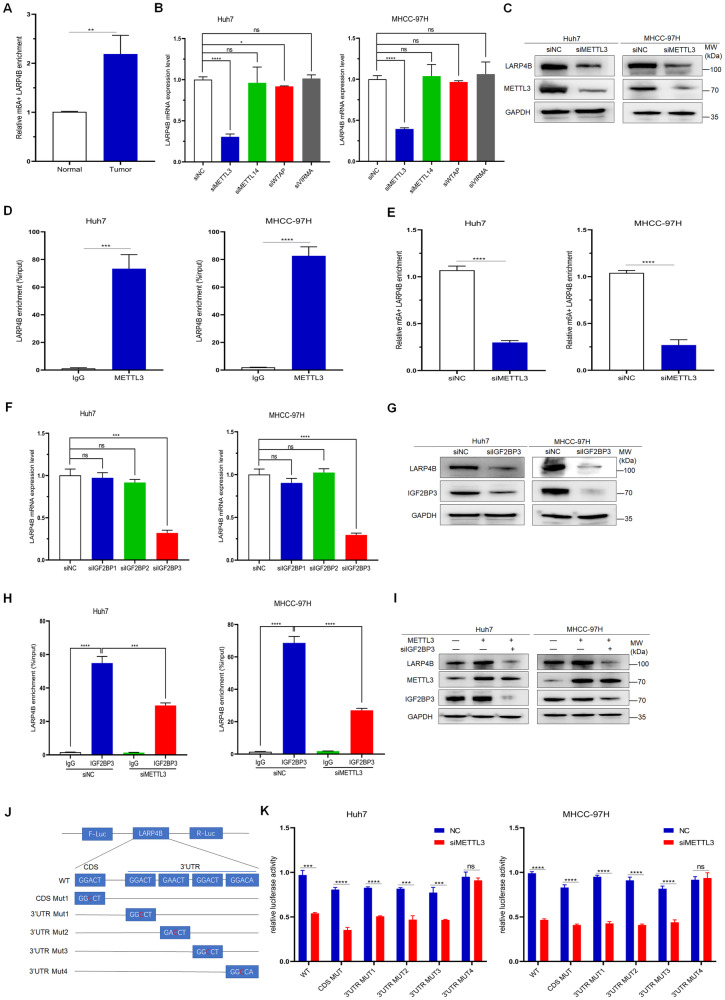
LARP4B expression is upregulated by METTL3-mediated m6A-IGF2BP3-dependent modification. **A** Methylated LARP4B mRNA levels in HCC tissues and adjacent normal tissues. **B** qPCR of LARP4B in Huh7 (left) and MHCC-97H (right) cells transfected with si“writers”. **C** Immunoblotting for LARP4B and METTL3 in Huh7 (left) and MHCC-97H (right) cells transfected with siNC or siMETTL3. **D** RIP-qPCR analysis of the enrichment of LARP4B pre-mRNA on METTL3 in Huh7 (left) and MHCC-97H (right) cells. **E** Levels of methylated LARP4B mRNA in Huh7 (left) and MHCC-97H (right) cells transfected with siNC or siMETTL3. **F** qPCR of LARP4B in Huh7 (left) and MHCC-97H (right) cells transfected with siIGF2BP1-3. **G** Immunoblotting for LARP4B and IGF2BP3 in Huh7 (left) and MHCC-97H (right) cells transfected with siNC or siIGF2BP3. **H** RIP-qPCR analysis of the enrichment of LARP4B pre-mRNA on IGF2BP3 in Huh7 (left) and MHCC-97H (right) cells transfected with siNC or siMETTL3. **I** Immunoblotting for LARP4B, METTL3, and IGF2BP3 in Huh7 (left) and MHCC-97H (right) cells transfected with METTL3 plasmid or siIGF2BP3. **J** Wild-type or mutant LARP4B was fused with a firefly luciferase reporter and a renilla luciferase reporter. **K** Relative luciferase activity of wild-type and mutant Huh7 (left) and MHCC-97H (right) cells transfected with siNC or siMETTL3. All results are presented as the mean ± SD. *P < 0.05, **P < 0.01, ***P < 0.001, ****P < 0.0001 and ns, no significance by a Mann–Whitney U test.

Epigenetic regulatory mechanisms, including N^6^-methyladenosine (m6A), have emerged as a focus of research in tumor physiopathology [17]. Given the elevated mRNA levels of LARP4B in HCC, we investigated whether m6A modifications play a crucial role in its dysregulation. A methylated RNA immunoprecipitation (MeRIP) assay revealed increased m6A levels in LARP4B mRNA in HCC tumor tissues compared to normal tissues (Fig. 2A). Therefore, we knocked down the main m6A methyltransferases (writers: METTL3, METTL14, WTAP, and VIRMA) and observed a reduction in LARP4B expression at both the mRNA and protein levels when METTL3 expression was silenced (Fig. 2B, C and Supplementary Fig. S2A). Similarly, METTL3 expression was higher in HCC tumor tissues than in adjacent normal tissues (Supplementary Fig. S2B). The datasets indicated a positive correlation between LARP4B and METTL3 mRNA expression (Supplementary Fig. S2C). RIP assays showed that METTL3 binds to LARP4B mRNA in HCC cells (Fig. 2D). MeRIP assays demonstrated that METTL3 knockdown reduced m6A levels in LARP4B mRNA (Fig. 2E). These data suggested that METTL3 binds to LARP4B mRNA and enhances its m6A levels.

Additionally, m6A readers, such as the YTH and IGF2BP families, can regulate RNA stability and translation by directly binding to m6A-modified RNA [18]. Western blot and quantitative polymerase chain reaction (qPCR) assays revealed that IGF2BP3 knockdown markedly suppressed LARP4B expression (Supplementary Fig. S2D and Fig. 2F, G). The datasets showed that LARP4B mRNA levels were positively associated with IGF2BP3 expression (Supplementary Fig. S2E). RIP assays indicated that the IGF2BP3 protein binds to LARP4B mRNA directly, and the amount of IGF2BP3 binding to LARP4B mRNA was reduced by METTL3 knockdown (Fig. 2H). Furthermore, METTL3 overexpression increased LARP4B expression, which was offset by IGF2BP3 knockdown (Fig. 2I).

To further explain the mechanism underlying the m6A modification of LARP4B mRNA, the online tool SRAMP (http://www.cuilab.cn/sramp) was used to predict m6A sites in LARP4B mRNA. We constructed wild-type and five mutant (CDS Mut, 3’UTR Mut1, 2, 3, 4) plasmids of the specific m6A motif (Fig. 2J), and the luciferase reporter assay revealed that METTL3 knockdown significantly decreased the relative luciferase activity of CDS Mut, 3’UTR Mut1, 2, and 3 but not 3’UTR Mut4 (Fig. 2K). These results indicate that METTL3 primarily increases the m6A level of LARP4B mRNA at the Mut4 site in the 3’UTR. In summary, these results suggest that METTL3 upregulates the expression of LARP4B via a m6A-IGF2BP3-dependent mechanism in HCC.

### LARP4B promotes stemness in HCC

**Fig. 3:**
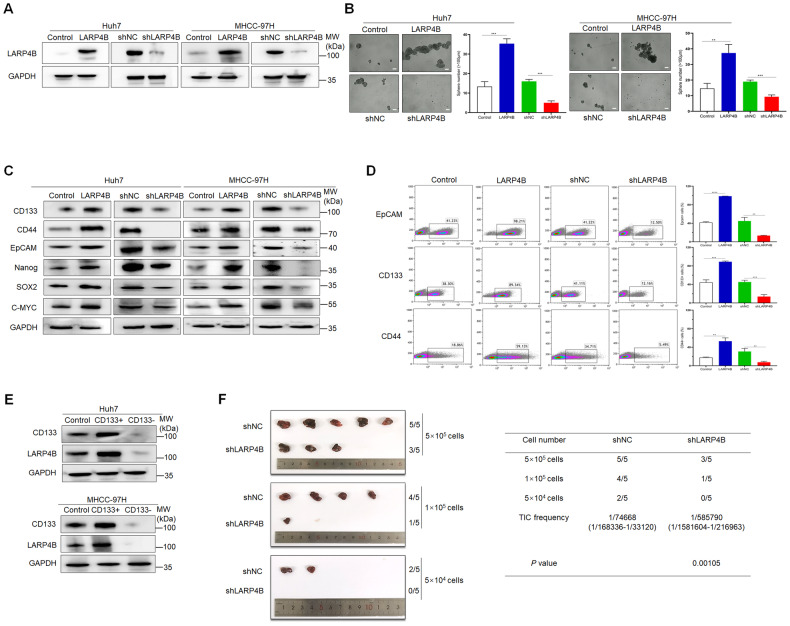
LARP4B promotes stemness in HCC. **A** Overexpression and knockdown of LARP4B in Huh7 (left) and MHCC-97H (right) cells were examined by western blotting. **B** Sphere formation of different groups of Huh7 (left) and MHCC-97H (right) cells. Scale bars: 500 µm. **C** Immunoblotting of stemness-related proteins in different groups of Huh7 (left) and MHCC-97H (right) cells. **D** Flow cytometric analysis of the EpCAM^+^, CD133^+^, and CD44^+^ cell populations in different groups of Huh7 cells. **E** Immunoblotting for LARP4B and CD133 in CD133^+^ and CD133^−^ cell populations sorted from Huh7 (upper) and MHCC-97H (bottom) cells. **F** Limiting dilution xenograft formation of Huh7 cells transfected with shNC or shLARP4B. All results are presented as the mean ± SD. **P < 0.01, ***P < 0.001 and ****P < 0.0001 by a two-tailed Student’s *t* test.

To investigate the role of LARP4B in HCC, three HCC cell lines (Huh7, MHCC-97H, and SNU-449) were established with stable overexpression or knockdown of LARP4B (Fig. 3A and Supplementary Fig. S3A). Mass spectrometry (MS) and RNA sequencing (RNA-seq) were used to analyze differences in the proteome and transcripts between shNC and shLARP4B cells. Gene Ontology (GO) and Kyoto Encyclopedia of Genes and Genomes (KEGG) analyses showed that cellular components, molecular signal transduction or MAPK signaling pathway reflected high-ranking downregulated pathways (Supplementary Fig. S3B–E). These sequencing data indicate that LARP4B is involved in cellular processes related to cancer and malignant transformation.

Activation of CSCs and maintenance of stemness are the initial stages of cancer progression. The GSE14520 dataset revealed a positive correlation between LARP4B and EpCAM expression, which was identified as a significant stemness marker (Supplementary Fig. S3F). Spheroid formation assays showed that LARP4B overexpression markably increased the number of spheres, whereas LARP4B knockdown decreased the number of spheres (Fig. 3B). Additionally, western blot analysis indicated that the expression of several biomarkers and transcription factors related to stemness was upregulated in cells overexpressing LARP4B, whereas LARP4B knockdown decreased their expression (Fig. 3C). Flow cytometric analysis demonstrated a higher positive proportion of EpCAM, CD133, and CD44 in cells overexpressing LARP4B and a decrease in the positive proportion in cells with LARP4B knockdown (Fig. 3D and Supplementary Fig. S3G). Western blot analysis showed that LARP4B expression was markedly higher in CD133^+^ cells than in CD133^−^ cells isolated from HCC cells by fluorescence-activated cell sorting (FACS) (Fig. 3E). Additionally, the limiting dilution assay showed that the probability of tumor formation was lower in the LARP4B knockdown group than in the control group (Fig. 3F), suggesting that LARP4B knockdown reduces the tumor initiation capacity of Huh7 cells. These findings demonstrate that LARP4B is closely related to and promotes stemness in HCC.

### LARP4B promotes HCC progression and LARP4B inhibition optimizes sorafenib efficacy

**Fig. 4:**
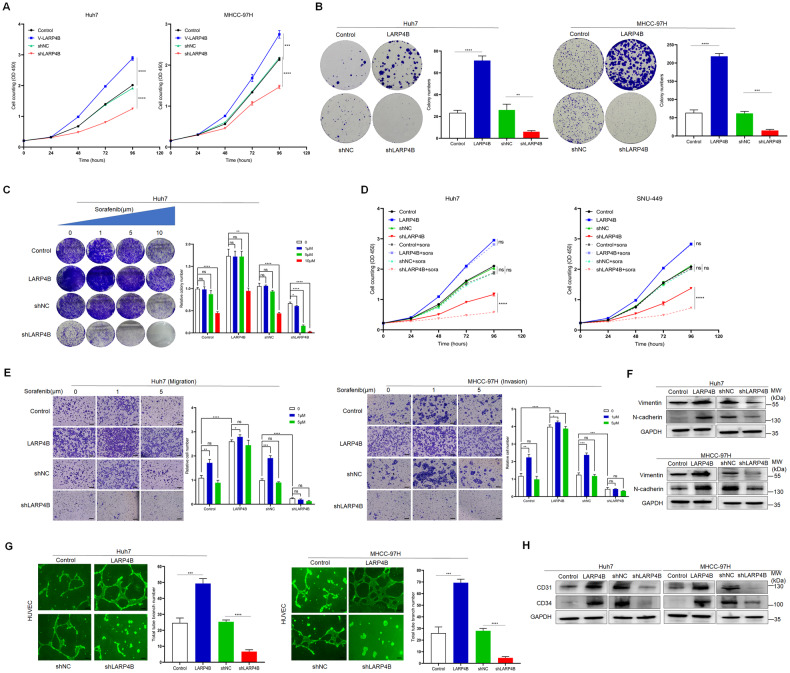
LARP4B promotes HCC progression and LARP4B inhibition optimizes sorafenib efficacy in vitro. Proliferation (**A**) and colony formation (**B**) of different groups of Huh7 (left) and MHCC-97H (right) cells. **C** Colony formation of different groups of Huh7 cells treated with 0, 1, 5, or 10 µm sorafenib. **D** Proliferation of different groups of Huh7 (left) and SNU-449 (right) cells treated with 0 or 5 µm sorafenib. **E** Migration (left) and invasion (right) of different groups of Huh7 or MHCC-97H cells treated with 0, 1, or 5 µm sorafenib. Scale bars: 100 µm. **F** Immunoblotting of Vimentin and N-cadherin in different groups of Huh7 (upper) and MHCC-97H (bottom) cells. **G** Tube formation of different groups of Huh7 (left) and MHCC-97H (right) cells. Scale bars: 200 µm. **H** Immunoblotting for CD31 and CD34 in different groups of Huh7 (left) and MHCC-97H (right) cells. All results are presented as the mean ± SD. *P < 0.05, **P < 0.01, ***P < 0.001 ****P < 0.0001 and ns, no significance by a Mann–Whitney U test for (**A**, **D**), a two-tailed Student’s *t-*test for (**B**, **C**, **E**, **G**).

Subsequently, we investigated whether LARP4B plays a role in malignancy and drug sensitivity. LARP4B overexpression significantly facilitated the proliferation and colony formation of HCC cells, whereas LARP4B knockdown weakened their proliferation and colony formation abilities (Fig. 4A, B). The colony formation assay also showed that LARP4B knockdown augmented the blockade of clonogenic proliferation by a lower concentration of sorafenib in Huh7 (1 or 5 µm) and SNU-449 (5 µm) cells (Fig. 4C and Supplementary Fig. S4A). CCK8 assays demonstrated the consistent result that LARP4B depletion sensitized HCC cells to a medium concentration of sorafenib (5 µm) (Fig. 4D). Additionally, transwell assays indicated that LARP4B overexpression promoted the migration and invasion of HCC cells, whereas LARP4B knockdown diminished the migration and invasion of HCC cells (Fig. 4E). Notably, the migration and invasion abilities of HCC cells were enhanced after treatment with a low concentration of sorafenib (1 µm); however, no enhancement was observed in cells with LARP4B knockdown (Fig. 4E). Western blot analysis showed that LARP4B overexpression upregulated the protein expression of Vimentin and N-cadherin. Conversely, LARP4B knockdown decreased the expression of these proteins, which are associated with epithelial-mesenchymal transition (EMT) (Fig. 4F). These findings suggest that LARP4B may be involved in HCC metastasis and that LARP4B depletion counteracts the metastasis-enhancing effect of low sorafenib concentrations.

When analyzing the results of IHC staining for LARP4B in HCC tissues, we noticed strong positivity in the capillary tubes (Supplementary Fig. S4B). RNA-seq data indicated a close relationship between LARP4B and the positive regulation of the angiogenesis pathway (Supplementary Fig. S3E). Subsequently, tube formation assays were performed in HUVECs and the results demonstrated that HUVECs cocultured with conditioned media (CM) from the LARP4B overexpression group exhibited significantly stronger tube formation capability, whereas the LARP4B knockdown group exhibited weakened tube formation capability (Fig. 4G). The vascular lineage markers CD31 and CD34 were also regulated by LARP4B (Fig. 4H). These results indicate that LARP4B promotes angiogenesis during HCC progression.

**Fig. 5:**
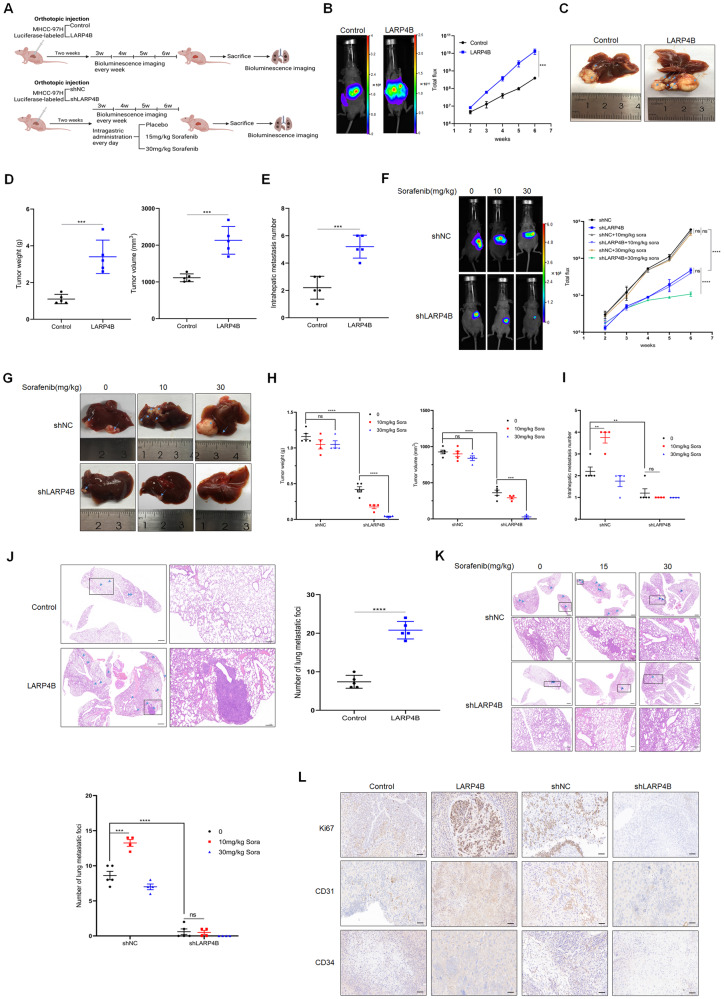
LARP4B promotes HCC progression and LARP4B inhibition optimizes sorafenib efficacy in vivo. **A** Study design for the liver orthotopic tumor model in BALB/c nude mice. **B**, **F** Bioluminescence imaging of mice at 2, 3, 4, 5, and 6 weeks after implantation. n = 5 for the 0 sorafenib group, and n = 4 for the 10 and 30 mg/kg sorafenib group. **C**, **G** Representative images of livers. **D**, **H** Tumor weight and volume of different groups. **E**, **I** Intrahepatic metastasis number of different groups. H&E staining of lung tissues in different groups and metastatic nodules were counted. Scale bars: 1000 µm (left) and 200 µm (right) for (**J**), 1000 µm (upper) and 200 µm (bottom) for (**K**). **L** IHC staining of ki67, CD31, and CD34 in liver tissues from different groups. Scale bars: 50 µm. All results are presented as the mean ± SD. **P < 0.01, ***P < 0.001, ****P < 0.0001 and ns, no significance by a Mann–Whitney U test for (**B**, **D**, **F**, **H**), a two-tailed Student’s *t-*test for (**E**, **I**, **J**, **K**).

An orthotopic HCC mouse model was constructed by the intrahepatic injection of different groups of stable luciferase-labeled MHCC-97H cells into the largest liver lobe of BALB/c nude mice. Subsequently, the mice were treated daily with different doses of sorafenib in the second week after injection (Fig. 5A). The results showed that LARP4B overexpression accelerated the growth of orthotopic tumors (Fig. 5B–D). Furthermore, LARP4B inhibition resulted in reduced tumor growth and enhanced tumor sensitivity during treatment with a medium dose of sorafenib (30 mg/kg) (Fig. 5F–H). Additionally, the number of IHMs increased in mice injected with cells overexpressing LARP4B (Fig. 5C, E). Conversely, LARP4B knockdown reduced the number of IHMs and neutralized the promotional effect of a low dose of sorafenib (10 mg/kg) (Fig. 5G, I). In vivo metastatic analysis showed that LARP4B facilitated lung metastasis, and the incidence of lung metastasis in the LARP4B overexpression group was 100% (Supplementary Fig. S4C and Fig. 5J). LARP4B knockdown hindered lung metastasis in mice and reduced the number of metastatic foci in the lungs (Supplementary Fig. S4D and Fig. 5K). However, a low dose of sorafenib (10 mg/kg) facilitated distant lung metastasis, which was deactivated by LARP4B knockdown (Supplementary Fig. S4D and Fig. 5K). In addition, IHC staining showed that LARP4B overexpression increased Ki67, CD31, and CD34 expression in tumor tissues, whereas LARP4B knockdown decreased its expression (Fig. 5L). In conclusion, LARP4B promoted tumorigenicity and progression in HCC, whereas LARP4B knockdown provided beneficial effects of sorafenib treatment, suggesting a potential therapeutic target for the treatment of HCC.

### LARP4B upregulates SPINK1 by binding to SPINK1 mRNA and maintaining its mRNA stability

**Fig. 6:**
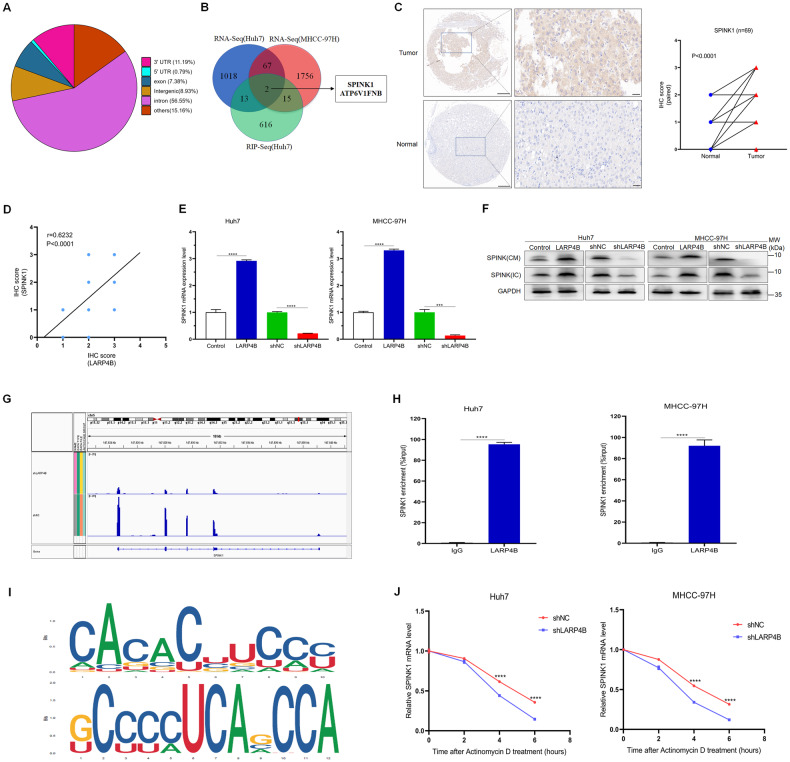
LARP4B upregulates SPINK1 by binding to SPINK1 mRNA and maintaining its mRNA stability. **A** Distribution of LARP4B-binding sites within different gene regions. **B** Venn diagram illustrating the overlapping targets of LARP4B identified by RNA-seq (Huh7 and MHCC-97H cells) and RIP-seq (Huh7 cells). **C** IHC staining of SPINK1 (left) and IHC staining scores (right) in a TMA. Scale bars: 200 µm (left) and 50 µm (right). **D** Correlation between IHC staining scores for LARP4B and SPINK1 in a TMA. **E** qPCR of SPINK1 in different groups of Huh7 (left) and MHCC-97H (right) cells. **F** Immunoblotting for SPINK1 in different groups of Huh7 (left) and MHCC-97H (right) cells. IC, intracellular samples; CM, conditioned media. **G** Integrative Genomics Viewer was used to visualize the distribution of reads on the SPINK1 gene. **H** RIP-qPCR analysis of the enrichment of SPINK1 pre-mRNA on LARP4B in Huh7 (left) and MHCC-97H (right) cells. **I** The LARP4B binding motifs were discovered. **J** qPCR of SPINK1 in Huh7 (left) and MHCC-97H (right) cells transfected with shNC or shLARP4B at 1–6 h after actinomycin D treatment. All results are presented as the mean ± SD. ***P < 0.001, ****P < 0.0001 and ns, no significance by a two-tailed Wilcoxon signed rank test for (**C**), Pearson’s correlation analysis for (**D**), Mann–Whitney U test for (**E**, **H**, **J**).

RNA-seq revealed that 1101 transcripts were significantly downregulated in Huh7 cells and 1844 in MHCC-97H cells (Supplementary Fig. S5A). To identify the downstream targets of LARP4B in HCC, RIP-sequencing (RIP-seq) was performed on Huh7 cells with or without stable LARP4B knockdown. The RIP-seq results showed that most reads were enriched in the intron and 3’UTR regions (Fig. 6A). Intersectional analysis of RNA- and RIP-seq data showed that two transcripts overlapped, namely, SPINK1 and ATP6V1FNB (Fig. 6B). ATP6V1FNB was expressed at extremely low levels in the liver, and its mRNA was not consistently upregulated when LARP4B was overexpressed (Supplementary Fig. S5B). Therefore, SPINK1 was identified as the primary candidate gene and was subsequently validated.

SPINK1, serine protease inhibitor Kazal type ɪ, is an autocrine or paracrine protein that is linked to the senescence-associated secretory phenotype (SASP) and cancer progression [19]. The GSE14520 dataset showed that SPINK1 mRNA expression was upregulated in HCC tissues (Supplementary Fig. S5C). Additionally, Western blot analysis and IHC staining demonstrated that SPINK1 protein expression was markedly higher in HCC tissues than in normal tissues, which also displayed a positive correlation with LARP4B protein (Supplementary Fig. S5D, E and Fig. 6C, D). Consistent with the RNA-seq results, LARP4B overexpression led to a marked increase in SPINK1 expression at both the mRNA and protein levels, and LARP4B knockdown resulted in a significant reduction in its expression, including the extracellular protein level (Fig. 6E, F and Supplementary Fig. S5F). Peak calling analysis by RIP-seq revealed that the enrichment of reads in the peaks was diminished upon LARP4B knockdown (Fig. 6G). RIP-qPCR analysis demonstrated that LARP4B binds to SPINK1 mRNA in HCC cells (Fig. 6H). Additionally, RIP-seq results showed several LARP4B binding motifs that had relatively higher scores (Fig. 6I). Furthermore, RNA decay assays demonstrated that LARP4B knockdown reduced the half-life of the SPINK1 transcript following treatment with actinomycin D (Fig. 6J). Taken together, these results suggest that LARP4B directly interacts with SPINK1 and upregulates SPINK1 expression by maintaining its mRNA stability.

### LARP4B upregulates SPINK1 expression and promotes HCC progression in a La motif-dependent manner

To further investigate whether LARP4B binding to SPINK1 mRNA is dependent on the La module, an HA-tagged mutant plasmid (ΔLM) that overexpressed the LARP4B gene but had a deletion of the La motif sequence was constructed. Compared to LARP4B-WT overexpression, LARP4B-ΔLM overexpression failed to increase SPINK1 expression at the mRNA and protein levels (Supplementary Fig. S6A–C). RIP-qPCR suggested that the binding efficacy between LARP4B and SPINK1 mRNA was intensified by LARP4B-WT overexpression, but not by LARP4B-ΔLM overexpression (Supplementary Fig. S6D). However, in vitro functional analyses revealed that LARP4B-ΔLM overexpression did not promote cell abilities related to sphere formation (Supplementary Fig. S6E), proliferation (Supplementary Fig. S6F), migration, invasion (Supplementary Fig. S6G), or tube formation (Supplementary Fig. S6H). Therefore, it can be concluded that LARP4B regulates SPINK1 expression and promotes stemness and tumorigenicity in HCC in a manner highly dependent on its specific La motif structure.

### LARP4B promotes HCC progression and impairs sorafenib efficacy by activating SPINK1-mediated EGFR pathways

**Fig. 7:**
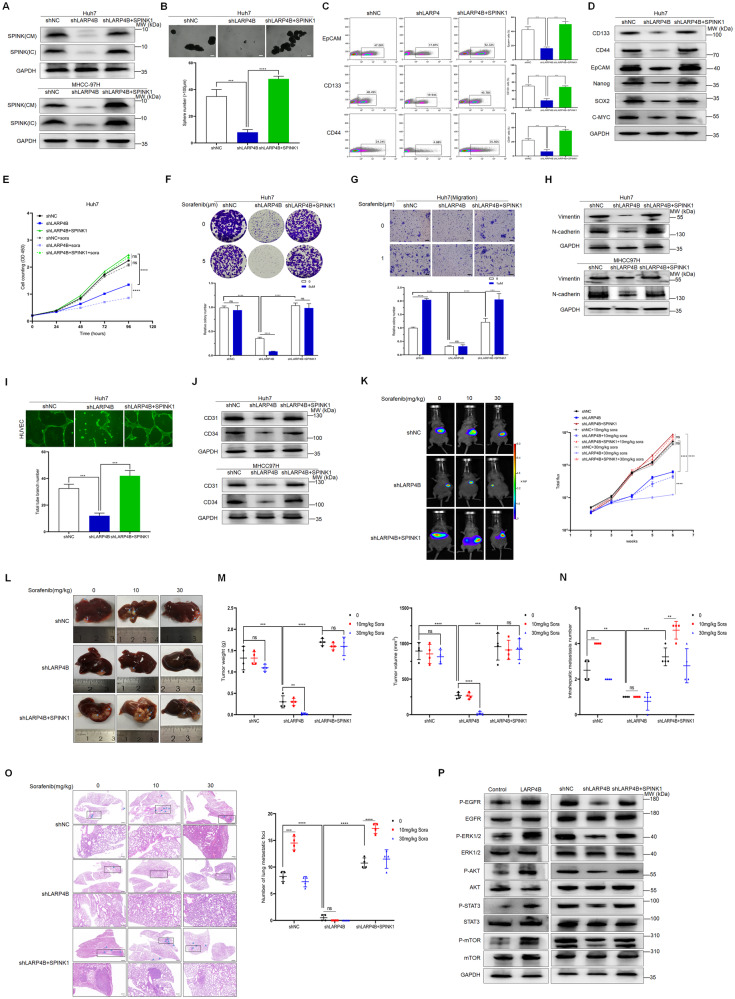
LARP4B promotes HCC progression and impairs sorafenib efficacy by activating SPINK1-mediated EGFR pathways. **A** Immunoblotting for SPINK1 in different groups of Huh7 (upper) and MHCC-97H (bottom) cells. IC, intracellular samples; CM, conditioned media. **B** Sphere formation of different groups of Huh7 cells. Scale bars: 500 µm. **C** Flow cytometric analysis of the EpCAM^+^, CD133^+^, and CD44^+^ cell populations in different groups of Huh7 cells. **D** Immunoblotting of stemness-related proteins in different groups of Huh7 cells. **E** Proliferation of different groups of Huh7 cells treated with 0 or 5 µm sorafenib. **F** Colony formation of different groups of Huh7 cells treated with 0 or 5 µm sorafenib. **G** Migration of different groups of Huh7 cells treated with 0 or 1 µm sorafenib. Scale bars: 100 µm. **H** Immunoblotting of Vimentin and N-cadherin in different groups of Huh7 (upper) and MHCC-97H (bottom) cells. **I** Tube formation of different groups of Huh7 cells. Scale bars: 200 µm. **J** Immunoblotting for CD31 and CD34 in different groups of Huh7 (upper) and MHCC-97H (bottom) cells. **K** Bioluminescence imaging of mice at 2, 3, 4, 5, and 6 weeks after implantation. n = 4 for each group. **L** Representative images of livers. **M** Tumor volume and weight of different groups. **N** Intrahepatic metastasis number of different groups. **O** H&E staining of lung tissues in different groups and metastatic nodules were counted. Scale bars: 1000 µm (upper) and 200 µm (bottom). **P** Immunoblotting of the indicated proteins and phosphorylation levels in different groups of Huh7 cells. All results are presented as the mean ± SD. ***P < 0.001, ****P < 0.0001 and ns, no significance by a two-tailed Student’s *t*-test for (**B**, **C**, **F**, **G**, **I**, **N**, **O**), Mann–Whitney U test for (**E**, **K**, **M**).

To determine whether LARP4B exerts its vital oncogenic function by upregulating SPINK1 in HCC, Huh7 and MHCC-97H cells overexpressing SPINK1 were used (Fig. 7A and Supplementary Fig. S7A). As expected, SPINK1 overexpression markedly reversed the inhibitory effects of LARP4B knockdown on the stemness of HCC cells, including sphere formation (Fig. 7B and Supplementary Fig. S7B), the proportion of EpCAM-, CD133-, and CD44- positive cells (Fig. 7C and Supplementary Fig. S7C) and the expression of stemness-related proteins (Fig. 7D and Supplementary Fig. S7D). Additionally, SPINK1 overexpression in LARP4B-knockdown cells rescued their proliferation and colony formation capabilities and reduced the sensitivity of cells to sorafenib treatment (5 µm) (Fig. 7E, F and Supplementary Fig. S7E, F). Moreover, SPINK1 overexpression reversed the reduction in migration and invasion abilities, and the expression of EMT-related proteins caused by LARP4B knockdown (Fig. 7G, H and Supplementary Fig. S7G). Notably, treatment with a low concentration of sorafenib (1 µm) promoted migration and invasion upon SPINK1 overexpression (Fig. 7G, H and Supplementary Fig. S7G). For angiogenesis, SPINK1 overexpression restored tube formation and increased the expression of CD31 and CD34 (Fig. 7I, J and Supplementary Fig. S7H). The data obtained from the in vivo experiments demonstrated that SPINK1 overexpression significantly rescued tumor growth and promoted the occurrence of IHM and lung metastasis (Fig. 7K–O and Supplementary Fig. S7I).

Notably, SPINK1, an EGF-like growth factor, shares approximately 50% sequence homology with EGF [19]. We found that LARP4B overexpression induced the phosphorylation of EGFR, ERK1/2, AKT, STAT3, and mTOR, whereas LARP4B knockdown inhibited the phosphorylation of these proteins (Fig. 7P and Supplementary Fig. S7J). However, this inhibitory effect was counteracted by elevated SPINK1 expression (Fig. 7P and Supplementary Fig. S7J). These results demonstrate that LARP4B imparts tumorigenesis traits and impairs sorafenib efficacy via EGFR downstream signaling induced by SPINK1.

### LARP4B is a potential target for sorafenib treatment of HCC by a positive feedback loop with the LARP4B/SPINK1/p-AKT/C/EBP-β axis

**Fig. 8:**
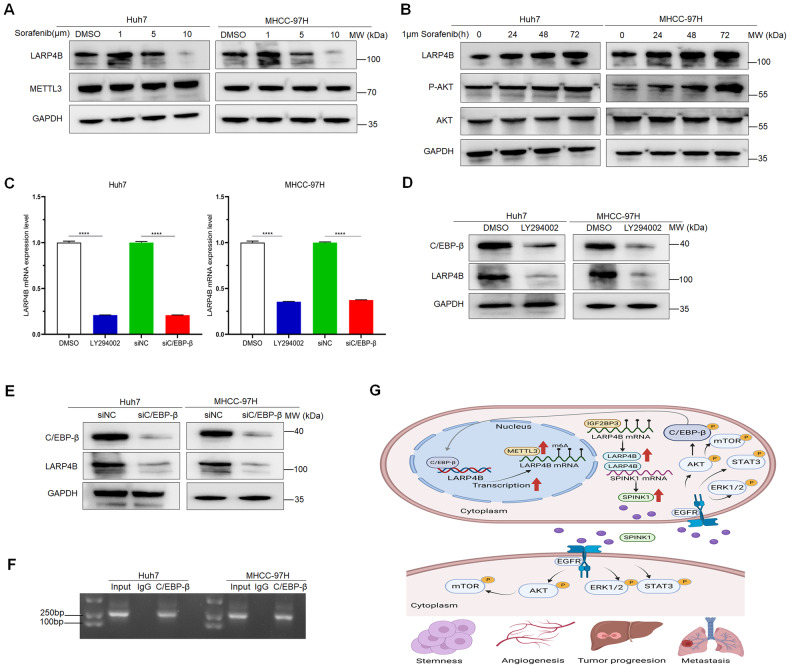
LARP4B is a potential target for sorafenib treatment of HCC by a positive feedback loop with the LARP4B/SPINK1/p-AKT/C/EBP-β axis. **A** Immunoblotting of LARP4B and METTL3 in Huh7 (left) and MHCC-97H (right) cells treated with DMSO, 1, 5, or 10 µm sorafenib. **B** Immunoblotting for LARP4B, p-AKT, and AKT in Huh7 (left) and MHCC-97H (right) cells at 0, 24, 48, and 72 h after treatment with 1 µm sorafenib. **C** qPCR of LARP4B in Huh7 (left) and MHCC-97H (right) cells treated with DMSO or LY294002 or transfected with siNC or siC/EBP-β. Immunoblotting for C/EBP-β and LARP4B in Huh7 (left) and MHCC-97H (right) cells treated with DMSO or LY294002 (**D**), or transfected with siNC or siC/EBP-β (**E**). **F** PCR gel showing amplification of C/EBP-β-binding sites after CHIP in Huh7 (left) and MHCC-97H (right) cells. **G** Graphical summary of the main finding. Schematic representation showing that LARP4B expression is upregulated by METTL3 via m6A-IGF2BP3-dependent modification during HCC progression. LARP4B upregulates SPINK1 expression by binding to SPINK1 mRNA and maintaining its mRNA stability. Overexpressed SPINK1 is secreted out of the cell and induces the activation of EGFR signaling pathways to stimulate stemness, angiogenesis, tumor progression, and metastasis in HCC. Additionally, a positive feedback loop of the LARP4B/SPINK1/p-AKT/C/EBP-β axis was identified and was responsible for the sorafenib-therapeutic benefit of LARP4B depletion. All results are presented as the mean ± SD. ****P < 0.0001 by a Mann–Whitney U test.

In this study, we found that LARP4B knockdown boosted the sensitivity of HCC cells to sorafenib and offset the metastasis-promoting effect of low doses of sorafenib; therefore, we explored the underlying mechanism. LARP4B expression was significantly reduced after treatment with 10 µm sorafenib, and slightly increased after treatment with 1 µm sorafenib (Fig. 8A). However, METTL3 expression was not affected by sorafenib treatment (Fig. 8A). To explain how sorafenib influences LARP4B expression, the cells were treated with four inhibitors of the signal transduction pathways that were inhibited by sorafenib: Raf/MEK/ERK, PI3K/Akt, NF-κB, and JAK/STAT3. The results showed that LY294002, an inhibitor of the PI3K/AKT pathway significantly inhibited LARP4B expression (Supplementary Fig. S8A). Similarly, high concentrations of sorafenib reduced AKT expression and phosphorylation, whereas low concentrations stimulated AKT phosphorylation (Supplementary Fig. S8B). Furthermore, exposure to 1 µm sorafenib promoted LARP4B expression and AKT phosphorylation in a time-dependent manner (Fig. 8B). Therefore, the PI3K/AKT pathway may mediate the interaction between sorafenib and LARP4B. We used JASPAR (http://jaspar.genereg.net) to explore the transcriptional regulatory mechanism of LARP4B expression and found that C/EBP-β, which is a downstream regulator of the PI3K/AKT pathway, regulates the expression of LARP4B by regulating its transcription. qPCR and western blot analyses showed that LY294002 reduced the expression of LARP4B and C/EBP-β (Fig. 8C, D), and C/EBP-β knockdown significantly reduced LARP4B expression (Fig. 8C, E). A chromatin immunoprecipitation (ChIP) assay further indicated that C/EBP-β bound to the promoter of the LARP4B gene in HCC cells (Fig. 8F). Taken together, these results demonstrate that sorafenib regulates LARP4B expression via the PI3K/AKT pathway. A positive feedback loop involving the LARP4B/ SPINK1/p-AKT/C/EBP-β axis could be the mechanism by which LARP4B knockdown confers benefits to sorafenib therapy.

## Discussion

m6A is the most abundant epigenetic modification in eukaryotic mRNA, and dysregulation of m6A contributes to the initiation, stemness, progression, and drug resistance of cancers [20]. Emerging studies have confirmed that METTL3 contributes to HCC progression [21, 22]. In this study, we found that METTL3 accomplishes m6A modification of LARP4B mRNA in HCC and that IGF2BP3 promotes the stability of LARP4B mRNA by binding to it. LARP4B is upregulated by this epigenetic process in HCC and is closely related to the poor prognosis of patients with HCC. Thus, LARP4B might be an oncogenic candidate for HCC development. In contrast, Koso et al. reported that LARP4B is a tumor suppressor in glioma that strongly inhibits cell proliferation by inducing mitotic arrest and apoptosis [23]. However, this difference in results could be explained by the heterogeneity of cancer.

Accumulating evidence has demonstrated that CSCs initiate tumor development, facilitate tumor progression, and induce acquired chemotherapy resistance in HCC [4]. Numerous intrinsic regulators are linked to stem cell markers, and diverse canonical signaling pathways are critical for maintaining the stemness of CSCs [24]. For instance, AKT, NFκB, and MAPK signaling are crucial for CD133^+^, CD24^+^, and EpCAM^+^ cells, respectively [24, 25]. Notably, EGFR pathways are now recognized as vital for the promotion and acquisition of stemness in cancer [6, 26]. In this study, gain- and loss-of-functional analyses showed that LARP4B promotes stemness and tumorigenicity in HCC. Furthermore, EGFR pathways are crucial for the LARP4B-mediated maintenance of stemness and tumorigenicity in HCC cells.

LARP4B overexpression promotes the maintenance of CSCs and expression of stemness-related proteins. It also triggered the emergence of EMT, increased the expression of vascularization-related markers, and reduced the sensitivity of HCC cells to sorafenib. The manifestation followed by LARP4B overexpression in HCC remarkably resembles the mechanism underlying the SASP in the TME. SASP refers to the secretion of cytokines, chemokines, and growth factors that favor the maintenance of stemness, tumorigenicity, malignancy, and drug resistance in cancer cells [19, 27]. However, the mechanism by which LARP4B overexpression results in a manifestation resembling that of the SASP deserves great attention. RIP- and RNA-seq analyses were used to identify SPINK1 as a key regulator of the crucial function of LARP4B in HCC. SPINK1, a SASP that exhibits EGF-like traits, is pivotal in regulating cancer progression, including stemness, proliferation, metastasis, and drug resistance [19, 28, 29]. In this study, we found that LARP4B promotes the stability of SPINK1 mRNA in HCC by directly binding to it through its specific La module. Upregulation of SPINK1 activates the EGFR signaling pathway, which further enhances the progression and malignancy of HCC.

Sorafenib, a multikinase inhibitor that blocks multiple growth factor pathways, is an approved first-line treatment for HCC [30]. However, a considerable number of patients with HCC are responseless to sorafenib and have an unfavorable prognosis owing to their rapid tumor progression [31]. Additionally, sorafenib stimulates the invasiveness and metastasis of HCC, ultimately leading to treatment failure. Compensatory activation of survival pathways, such as the EGFR pathway, upon sorafenib treatment is responsible for drug resistance and enhanced metastasis [32, 33]. In this study, we discovered that a low dose of sorafenib enhanced the migratory and invasive capabilities of HCC cells and their potential for tumor metastasis. Notably, LARP4B knockdown disrupts the metastasis-promoting role of a low dose of sorafenib and sensitizes HCC cells to a medium dose of sorafenib by inhibiting the activation of the EGFR pathway. Interestingly, different concentrations of sorafenib had varying effects on LARP4B expression. To explain the findings of our study, a positive feedback loop of the LARP4B/SPINK1/p-AKT/C/EBP-β axis was identified. LARP4B inhibition blocks this feedback loop, providing a promising therapeutic approach for treating HCC. For clinical applications, the detection of LARP4B expression in HCC tissues could predict the efficacy of sorafenib treatment, and HCC patients with higher expression of LARP4B should receive an increased dosage of sorafenib. Furthermore, the combination of delivering LARP4B siRNA by nanoparticles and reduced dosage of sorafenib could improve therapeutic effects and mitigate drug toxicity.

In conclusion, our findings demonstrate that LARP4B, which is upregulated by m6A modification, promotes stemness and tumorigenesis by activating the SPINK1-induced EGFR signaling pathway in HCC. LARP4B inhibition enhances the antitumor effect of sorafenib and blocks the metastasis-enhancing effect of low concentrations of sorafenib by a positive feedback loop of the LARP4B/SPINK1/p-AKT/C/EBP-β axis. Therefore, LARP4B may be a potentially effective therapeutic target for HCC, and combination therapy with LARP4B depletion and sorafenib may provide benefits for patients with HCC (Fig. 8G).

## Materials and methods

### Antibodies and primers

Details of the antibodies used for immunohistochemistry (IHC), western blotting, IP, and flow cytometry (FC) are shown in Supplementary Table S2. Details of the primers used for qPCR and CHIP are shown in Supplementary Table S3.

### Patient specimens and tissue microarray construction

Eighty-one pairs of tissues were obtained from patients with HCC who underwent surgery at Xinqiao Hospital. Patients who underwent chemotherapy or radiotherapy were excluded. The protocols used in this study were approved by the Ethical Review Committees of Army Medical University, and written informed consent was obtained from all patients. The clinicopathological data of all patients was collected and analyzed. Tissues were fixed in formalin, embedded in paraffin, extracted, and fixed in a paraffin block using an automated tissue array instrument (Minicore3, Alphelys, Plaisir, France). Subsequently, the tissue microarray (TMA) blocks were sectioned into 3 µm-thick slides for immunohistochemistry analysis.

### Immunohistochemistry (IHC) and hematoxylin-eosin (H&E) staining

Tissue sections and embedded TMAs were deparaffinized, rehydrated, blocked, and subjected to antigen retrieval. Subsequently, sections or TMAs were incubated with primary antibodies overnight at 4 °C and with secondary antibodies for 1 h at room temperature. Images were taken using an Olympus microscope and scored by the percentage of positive cells and the staining intensity. Mouse liver and lung tissues were fixed in 4% paraformaldehyde overnight at 4 °C for subsequent paraffin-embedding and sections were prepared for H&E staining and IHC.

### Cell lines and cell culture

The Huh7, MHCC-97, and SNU-449 cell lines were obtained from the Shanghai Cell Bank Type Culture Collection Committee (Shanghai, China). Huh7 and MHCC-97H cells were cultured in Dulbecco’s modified Eagle’s medium (DMEM) with 10% fetal bovine serum (FBS, Gibco, USA), and SNU-449 cells were maintained in RPMI-1640 supplemented with 10% FBS. All cells were cultured at 37 °C with 5% CO_2_.

### Cell transfection

To establish stable LARP4B- overexpressing and LARP4B- knockdown cells and SPINK1- overexpressing cells, lentiviruses expressing LARP4B or empty vector, shLARP4Bor shNC, and SPINK1 or empty vector were obtained from GENE (Shanghai, China). Small interfering RNAs targeting METTL3, METTL14, WTAP, VIRMA, IGF2BP1-3, YTHDF1-3, and C/EBP-β or negative controls were synthesized and obtained from RiboBio (Guangzhou, China). The plasmid with full-length complementary cDNA of human METTL3 and the LARP4B-mutant plasmid with a depleted La module sequence were obtained from GENE. For the luciferase reporter assays, pmirGLO-LARP4B-WT and pmirGLO-LARP4B-MUT were designed by OBiO (Shanghai, China). The siRNAs and plasmids were transfected into cells using Lipofectamine 3000 (Invitrogen).

### Preparation of conditioned medium (CM)

Cells were cultured on six-well plates for 24 h. The medium was removed, and the cells were cultured in serum-free medium at 37 °C for 24 h. The CM was collected and purified using PES with a 3 K molecular weight cutoff (Thermo Fisher, MA, USA).

### Western blot analysis

Tissues and cells were lysed, electrophoresed on 4–20% SDS-PAGE gels, and transferred onto polyvinylidene fluoride (PVDF) membranes. After blocking with 5% milk for 1 h, the membranes were incubated with primary antibodies overnight at 4 °C and then incubated with secondary antibodies for 1 h at room temperature. The blots were imaged using a chemiluminescence system.

### RNA extraction and quantitative real-time PCR (qPCR) assay

Total RNA was extracted from cells and tissues using TRIzol reagent (Invitrogen). cDNA was generated using a cDNA Reverse Transcription Kit (TakaraBio, Beijing, China). qPCR was performed using SYBR Green Dye (TakaraBio). All results were analyzed using the 2-ΔΔCt method by normalizing to β-actin.

### Mass spectrometry (MS), RNA sequencing (RNA-seq), and bioinformatics analysis

Huh7 cells transfected with shLARP4B and shNC were lysed in RIPA buffer and subjected to MS analysis on Q Exactive HFX performed by BGI (Shenzhen, China). Total RNA from Huh7 and MHCC-97H cells was isolated using TRIzol reagent (Invitrogen). Next-generation sequencing was performed by DIATRE Inc. (Shanghai, China). Genes with threshold values of |log2FC| > 1 and *P* value < 0.05 were selected and subjected to KEGG and GEO analyses.

### Sphere formation assay

A total of 3 × 10^3^ cells were plated in 6-well ultralow adherence polystyrene plates (Corning, NY, USA) and cultured in DMEM/F12 with B27 (1:50, Gibco, NY, USA), 20 ng/ml EGF (R&D Systems, Minneapolis, MN, USA), and 10 ng/ml bFGF (R&D Systems). After 10 days in a 37 °C with 5% CO_2_, photomicrographs of spheres were taken.

### Flow cytometry (FC) and cell sorting

The cells were digested, counted, stained with antibodies, and analyzed using a Navier Flow Cytometer (Beckman Coulter, Miami, FL, USA). To sort CD133^+^ cells, Huh7 and MHCC-97H cells were incubated with a phycoerythrin-conjugated anti-human CD133 antibody (MiltenyiBiotec, Bergisch Gladbach, Germany) and sorted by FACS.

### Cell proliferation assay

A total of 3 × 10^3^ cells were plated in 96-well plates in medium with or without different concentrations of sorafenib. Cell proliferation was measured using the Cell Counting Kit 8 (CCK8) (Dojindo, Tokyo, Japan).

### Colony formation assay

A total of 1 × 10^3^ cells were plated in 6-well plates and cultured in media with or without different concentrations of sorafenib for 10–14 days. Cells were fixed with 4% formaldehyde, stained with 2% crystal violet, and photographed.

### Migration and invasion assay

Different groups of cells were seeded into 24-well culture inserts with 8 µm pores. The upper chamber was suspended in serum-free medium with or without different concentrations of sorafenib, and 600 µl of DMEM containing 10% FBS was added to the lower chamber. For the invasion assay, inserts were first coated with Matrigel (BD, Biosciences) for 12 h. After 24–36 h, the cells were fixed in 4% paraformaldehyde and stained with 0.2% crystal violet. The number of cells passing through the basement membrane was counted under an optical microscope (Olympus, Tokyo, Japan), and representative images were collected.

### Tube formation assay

Culture supernatants from different groups of Huh7 and MHCC-97H cells were collected. HUVECs were cultured in 24-well plates that were precoated with 50 µl Geltrex (Thermo Fisher) with the collected culture supernatants for 12 h and then stained with calcein-AM (Beyotime, Shanghai, China). Tube junctions were observed using a fluorescence microscope.

### Animal studies

All animal experiments were performed in accordance with the Guidelines for the Care and Use of Laboratory Animals and approved by the Laboratory Animal Welfare and Ethics Committee of the Third Military Medical University. Six- to eight-week-old male BALB/c (nu/nu) mice were purchased from GemPharmatech (Chengdu, China) and randomly divided into indicated groups. For the in vivo limiting dilution assay, 5 × 10^4^, 1 × 10^5^, and 5 × 10^5^ Huh7 cells transfected with shLARP4B or shNC were subcutaneously injected into the right dorsal flank of the mice. After four weeks, the mice were sacrificed, tumor images were obtained, and tumor incidence rates were counted and analyzed. Extreme limiting dilution analysis (ELDA) was used to estimate stem cell frequency and the significance of differences between treatment groups at 95% confidence intervals. For orthotopic liver injection, 2 × 10^6^ luciferase-labeled different groups of MHCC-97H cells were mixed with Matrigel and injected into the right lobe of the liver under anesthesia. After two weeks, the mice were administered either a daily oral dose of 15 mg/kg or 30 mg/kg sorafenib or a daily oral dose of placebo solution. In vivo tumor formation was monitored by bioluminescence imaging using an IVIS 100 Imaging System (Perkin Elmer, Waltham, MA, USA). At six weeks, the mice were sacrificed, and metastasis was reflected by detecting the intensity of bioluminescence signals in the lungs. Finally, the liver and lungs were collected and subjected to histological examination. The size and weight of the tumors in the liver were measured, and tumor volumes were calculated using the formula V = (length × width^2^)/2.

### RNA immunoprecipitation (RIP)

The RIP assay was performed using a Magna RIP Kit (Millipore, MA, USA) according to the manufacturer’s instructions. Huh7 and MHCC-97H cells were lysed with RIP lysis buffer and incubated with 5 µg of METTL3, IGF2BP3, or LARP4B antibodies on beads overnight at 4 °C. Subsequently, the RNA-protein complexes were washed and incubated with proteinase K digestion buffer. RNA was extracted by the phenol-chloroform RNA extraction method. The relative interactions between mRNA and protein were determined by qPCR and normalized to the input.

### Methylated RNA immunoprecipitation (MeRIP)

The m6A methylation level of LARP4B mRNA was determined using the Magna MeRIP m6A kit (Millipore). Total RNA was extracted from the tissues and cells, and mRNA was purified using the Dynabeads mRNA Purification Kit (Invitrogen). Subsequently, purified mRNA was digested by DNase I, fragmented into ~100 nt fragments, and incubated at 94 °C. The anti-m6A antibody was preincubated with 5 µl of beads in IP buffer at room temperature for 1 h. Fragment mRNAs were incubated with an antibody-bead mixture at 4 °C for 4 h on a rotator. The immunoprecipitated mixture was digested with proteinase K and washed. mRNA was extracted and purified using a RNeasy MinElute Cleanup Kit (QIAGEN, Germany). Relative m^6^A levels of mRNA were determined using qPCR and normalized to the input.

### Dual-luciferase reporter assay

A luciferase reporter assay was performed using the Dual-Luciferase Reporter Assay System (Promega, Madison, WI, USA). The Wild-type pmirGLO-LARP4B-WT reporter plasmid was cloned by inserting the CDS and 3’UTR of the LARP4B transcript after the Fluc coding sequence. The mutant pmirGLO-LARP4B-CDS or 3’UTR reporter plasmids were made by replacing the adenosine bases within the m6A consensus sequences with cytosine. Huh7 and MHCC-97H cells transfected with siMETTL3 or siNC were seeded in 24-well plates and transfected with wild-type or mutated-LARP4B plasmids. After 24 h, the cells were harvested, and luciferase activity was detected using a Synergy Mx Multi-Mode Microplate Reader (BioTek, Winooski, VT, USA). The relative luciferase activity was examined by firefly luciferase activity normalized to renilla luciferase activity.

### RIP sequencing

Stable Huh7 cells transfected with shLARP4B and shNC were subjected to RIP with the anti-LARP4B antibody as previously described. RNAs bound to LARP4B were extracted, and rRNAs were removed using the NEBNext rRNA depletion kit (NEB, Ipswich, MA, USA). rRNA-depleted RNAs were constructed from RNA sequencing libraries using the NEBNext UltraII Directional RNA Library Prep Kit (NEB), performed by DIATRE (Shanghai).

### Actinomycin D assay

The cells were treated with 100 ng/ml actinomycin D (Merck, Darmstadt, Germany) for 0, 4, 8, 12, and 24 h. Total RNA was extracted from cells at the indicated times. SPINK1 mRNA expression was analyzed using qPCR.

### Enzyme-linked immunosorbent assay (ELISA)

Culture supernatants from different groups of Huh7 and MHCC-97H cells were collected. The concentration of SPINK1 in the culture supernatant was measured by a Human SPINK1 Elisa kit (R&D systems, DY7496) according to the manufacturer’s instructions. Firstly, 96-well plates were incubated with diluted capture antibody overnight at room temperature and then blocked with reagent diluent at room temperature for 1 h. The plates were then incubated with samples and antibody for 2 h at room temperature. Streptavidin-HRP and substrate solution were added to each well followed by incubating for 20 min at room temperature. Finally, the plates were added with stop solution and detected using a microplate reader set to 450 nm.

### Chromatin immunoprecipitation (ChIP) assay

The ChIP assay was performed using a ChIP assay kit (Millipore). Huh7 and MHCC-97H cells were fixed with 1% formaldehyde to covalently crosslink proteins to DNA, and chromatin was harvested. Then, DNA was fragmented to 200–1000 base pairs in length by sonication and incubated with anti-C/EBP-β antibody. Finally, PCR was performed to enrich the DNA fragments in the LARP4B promoter using specific primers. Control immunoprecipitations were performed using a mouse IgG antibody.

### Statistical analysis

All in vitro experiments were performed in triplicate, and the data in the figures are presented as the mean ± SD. The chi-squared test was used to analyze the relationships between LARP4B expression and clinicopathological variables. Cox proportional hazards regression model and multivariate Cox proportional hazards model analyses were performed with SPSS statistical software. Statistical significance was determined by two-tailed Student’s *t*-test, log-rank test, two-tailed Wilcoxon signed rank test, Pearson’s correlation test, or Mann–Whitney U test. For all statistical tests, a *P* value < 0.05 was considered significant.

### Supplementary information

Supplementary Figure S1

Supplementary Figure S2

Supplementary Figure S3

Supplementary Figure S4

Supplementary Figure S5

Supplementary Figure S6

Supplementary Figure S7

Supplementary Figure S8

Supplementary file 1

Supplementary file 2

## Data Availability

The raw gene expression data in HCC were downloaded from the Gene Expression Omnibus (GEO) database as follows: GSE14520. GSE77314. Any other data are available upon reasonable request to the corresponding author.
