# Supplementary figures and images for: LARP4B promotes hepatocellular carcinoma progression and impairs sorafenib efficacy by activating SPINK1-mediated EGFR pathway
Source: Cell Death Discov. 2024 May 1;10:208. doi: 10.1038/s41420-024-01985-6 (PMC11063073; doi:10.1038/s41420-024-01985-6)

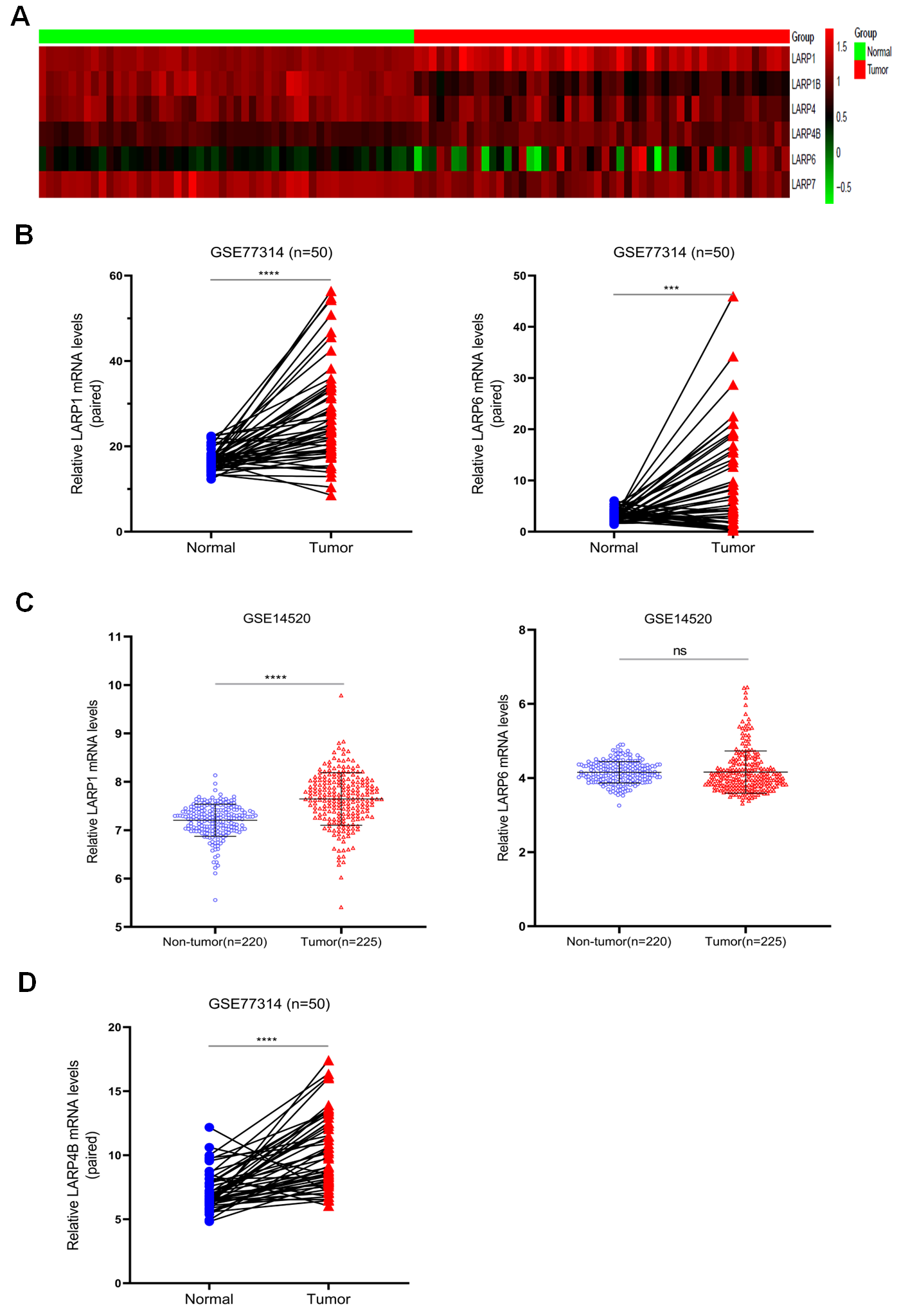

Supplement: Supplementary file 1 — Supplementary Figure S1 [file 41420_2024_1985_MOESM1_ESM.tif]

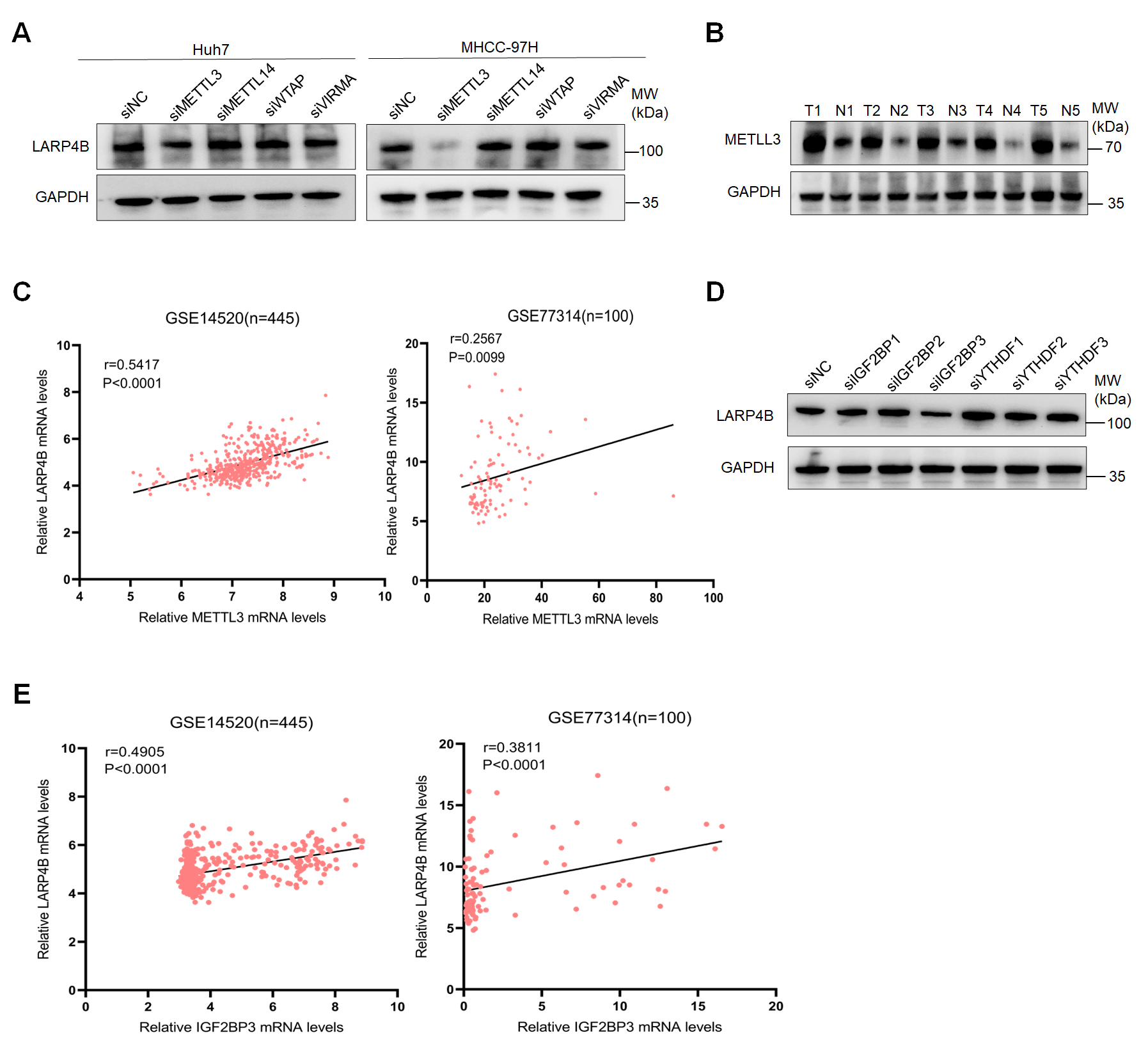

Supplement: Supplementary file 2 — Supplementary Figure S2 [file 41420_2024_1985_MOESM2_ESM.tif]

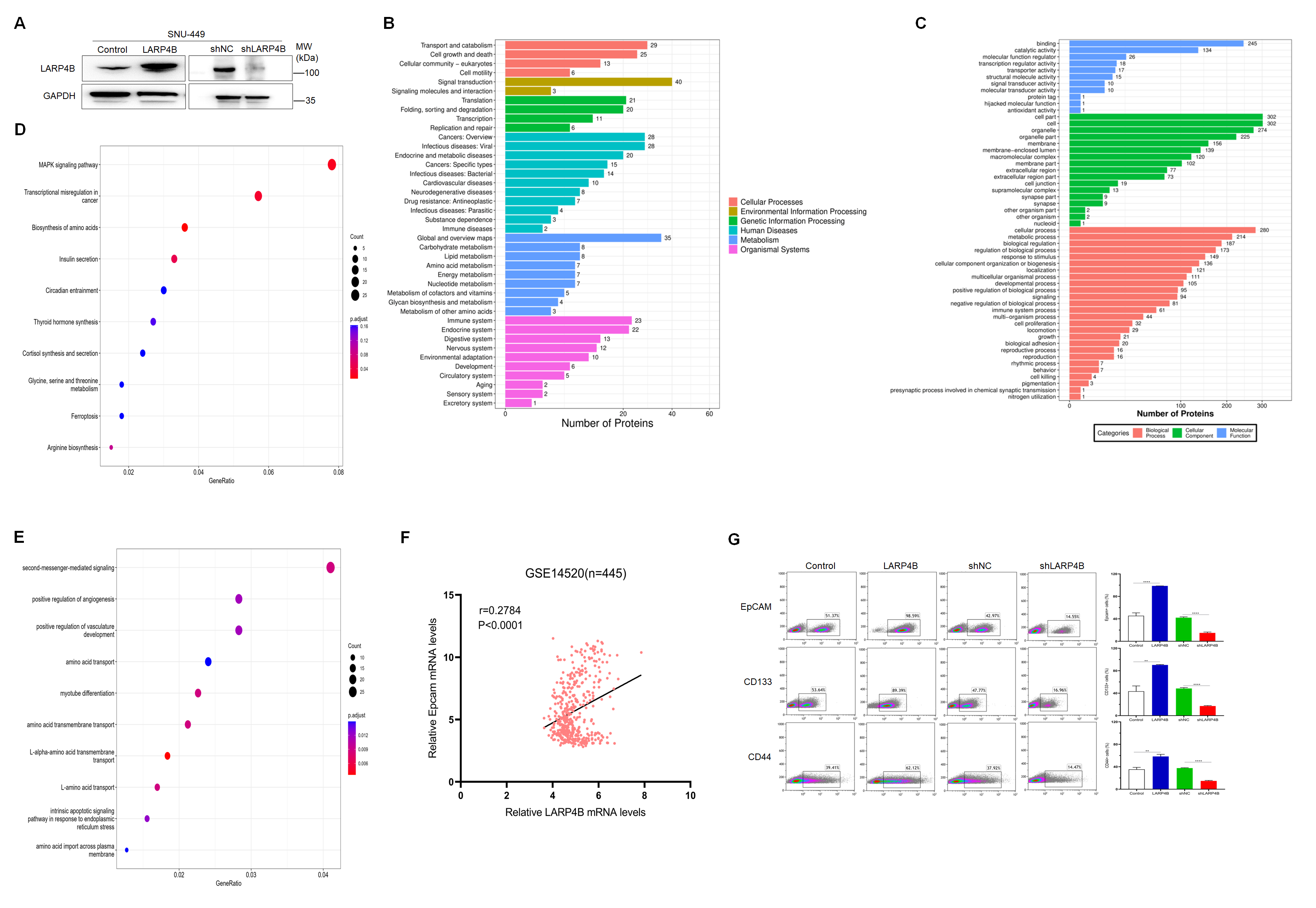

Supplement: Supplementary file 3 — Supplementary Figure S3 [file 41420_2024_1985_MOESM3_ESM.tif]

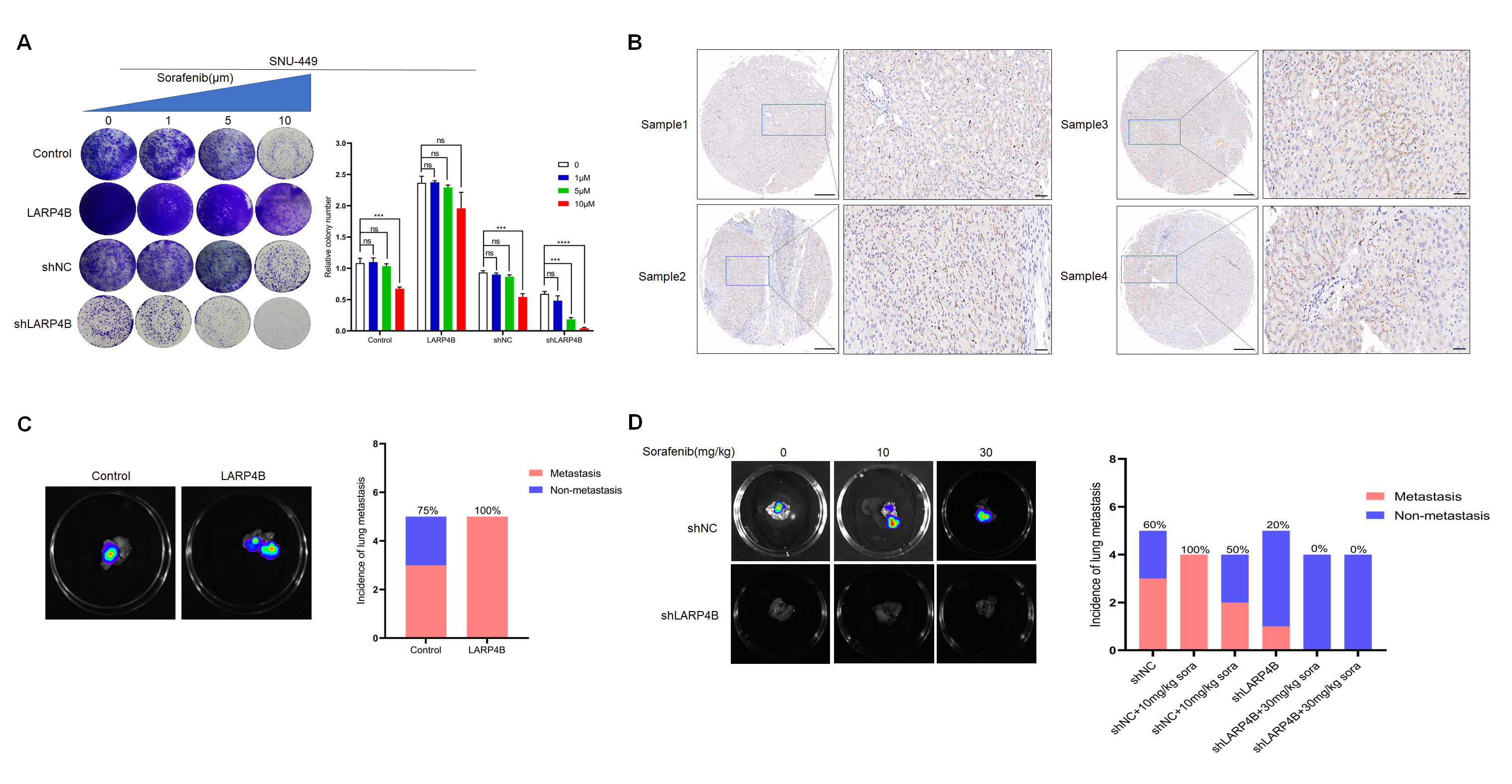

Supplement: Supplementary file 4 — Supplementary Figure S4 [file 41420_2024_1985_MOESM4_ESM.tif]

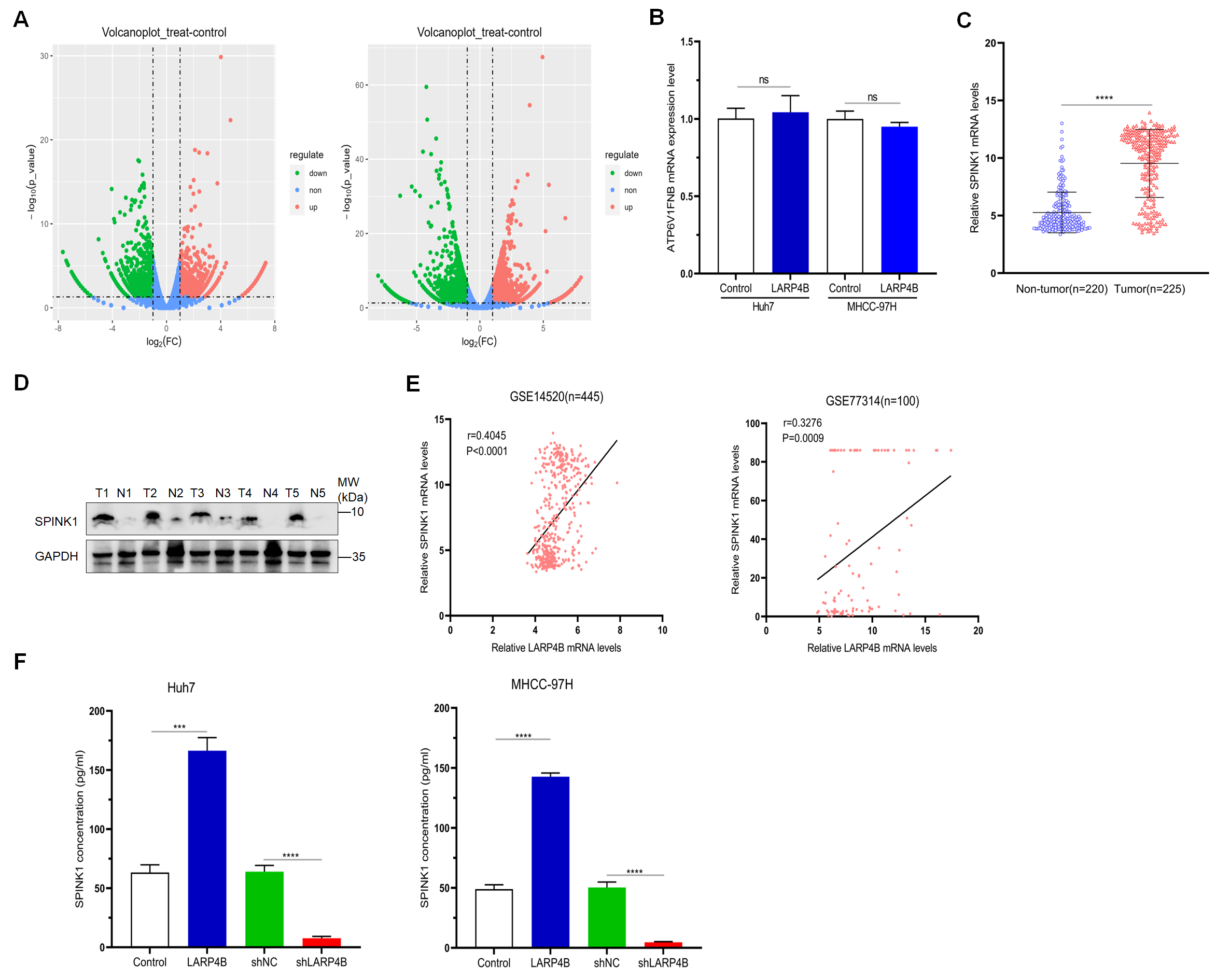

Supplement: Supplementary file 5 — Supplementary Figure S5 [file 41420_2024_1985_MOESM5_ESM.tif]

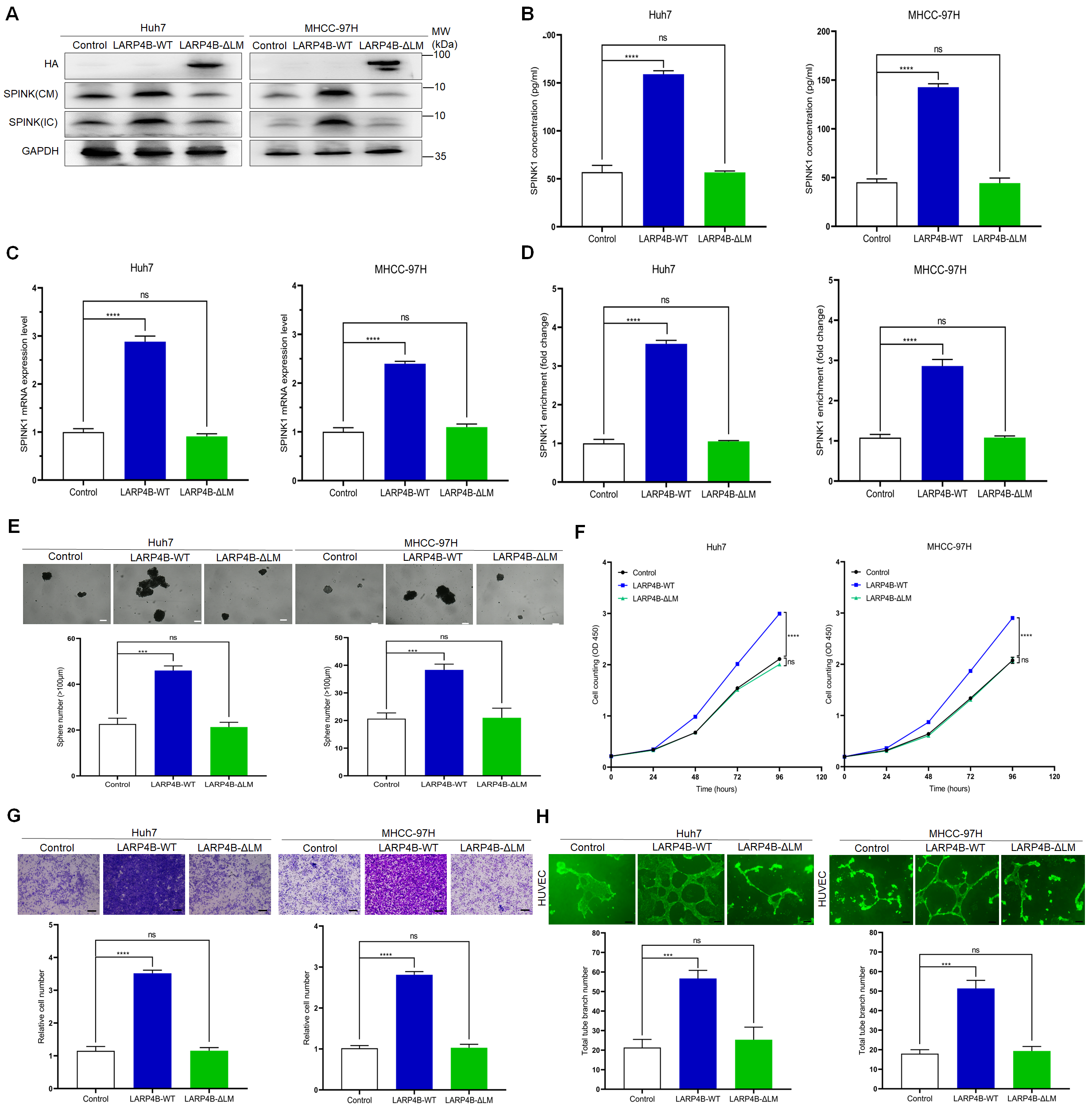

Supplement: Supplementary file 6 — Supplementary Figure S6 [file 41420_2024_1985_MOESM6_ESM.tif]

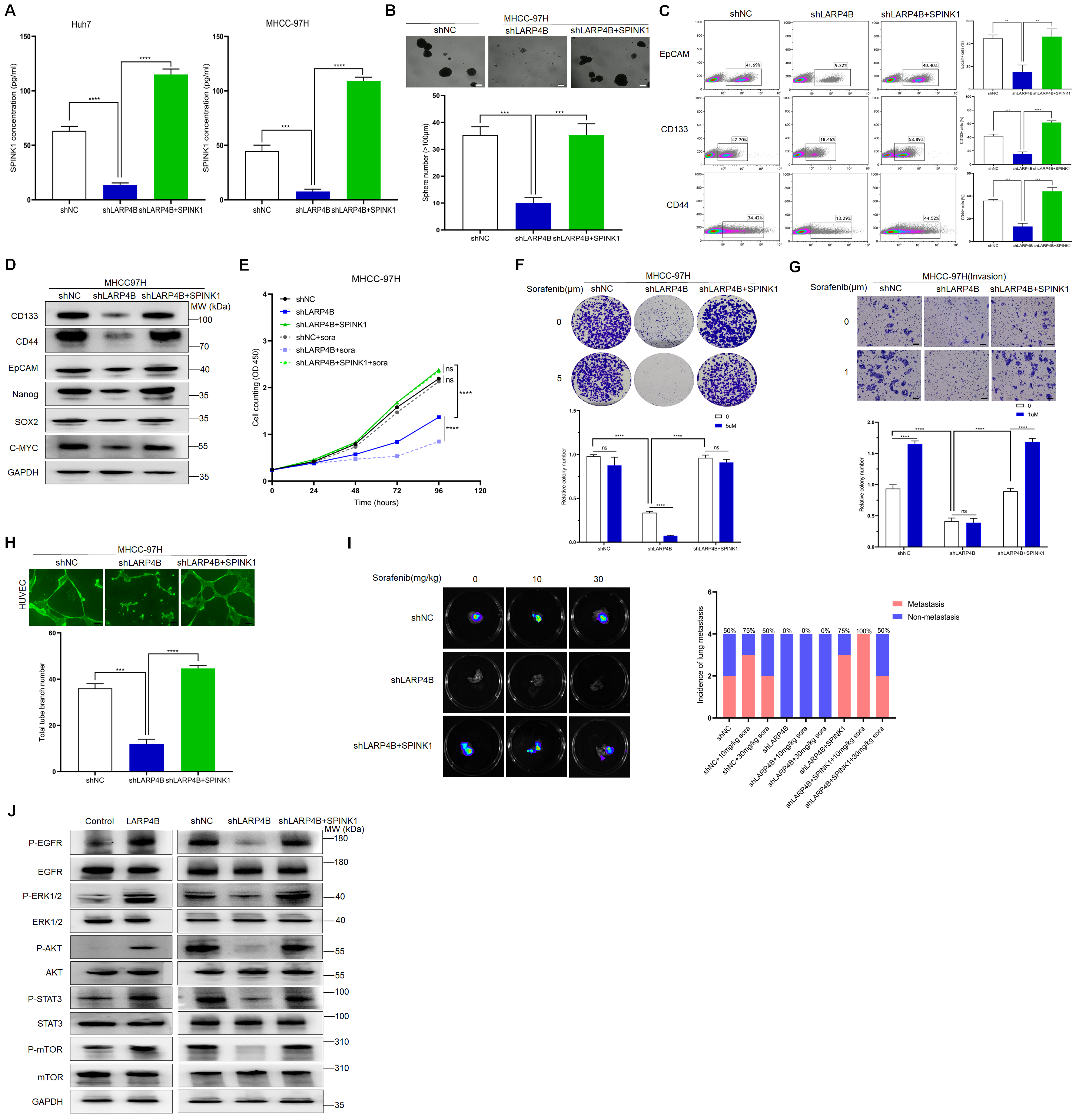

Supplement: Supplementary file 7 — Supplementary Figure S7 [file 41420_2024_1985_MOESM7_ESM.tif]

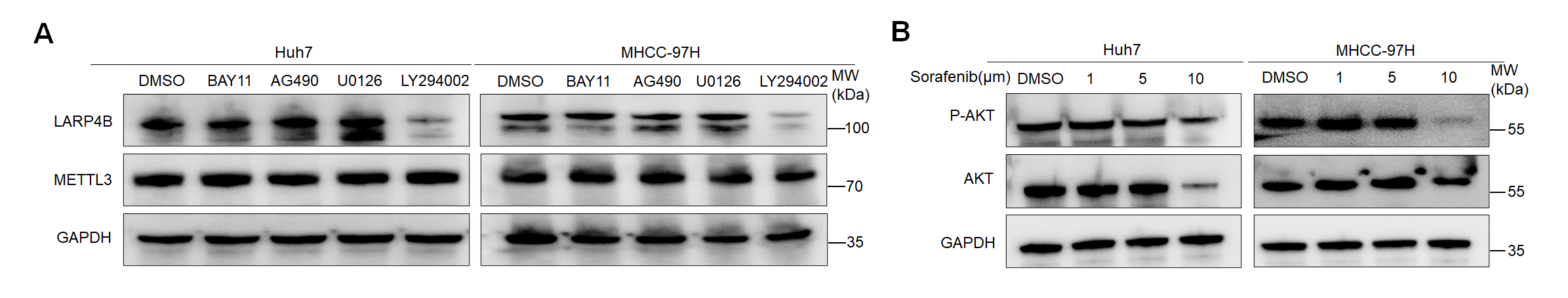

Supplement: Supplementary file 8 — Supplementary Figure S8 [file 41420_2024_1985_MOESM8_ESM.tif]
