# Supplementary material for: LARP4B promotes hepatocellular carcinoma progression and impairs sorafenib efficacy by activating SPINK1-mediated EGFR pathway
Source: Cell Death Discov. 2024 May 1;10:208. doi: 10.1038/s41420-024-01985-6 (PMC11063073; doi:10.1038/s41420-024-01985-6)
Supplement: Supplementary file 9 — Supplementary file 1 [file 41420_2024_1985_MOESM9_ESM.docx]

**Supplementary file 1**

**LARP4B promotes hepatocellular carcinoma progression and impairs sorafenib efficacy by activating SPINK1-mediated EGFR pathway**

Chuanxu Wang^1^, Rui Dong^2, 3^, Feicheng Yang^4^, Lu Zheng^1^, Yingling Liu^1^, Yue Yan^1^, Mengjie Zhang^2^, Bing Ni^2^, and Jing Li^1^

Chuanxu Wang and Rui Dong contributed equally to this article.

^1^Department of Hepatobiliary Surgery, Xinqiao Hospital, Third Military Medical University (Army Medical University), Chongqing, China; ^2^Department of Pathophysiology, Third Military Medical University (Army Medical University), Chongqing, China. ^3^Chongqing International Institute for Immunology, Chongqing, China; ^4^Department of Pathology, the First Affiliated Hospital of Hunan Normal University (Hunan Provincial People’s Hospital), Changsha, China.

**Correspondence:**

Jing Li, Department of Hepatobiliary Surgery, Xinqiao Hospital, Third Military Medical University (Army Medical University), 83 Xinqiao Centre street, 400037 Chongqing, China. Phone: +8602368774506; E-mail: [xqyylj@163.com](mailto:xqyylj@163.com) and Bing Ni, Department of Pathophysiology, Third Military Medical University (Army Medical University), 30 Gaotanyan Centre street, 400037 Chongqing, China. [nibing@tmmu.edu.cn](mailto:nibing@tmmu.edu.cn).

**Supplementary figure legends**

**Fig. S1** The expression of LARPs in HCC. **A** Heatmap profiling of LARPs expression in GEO databases. **B, C** LARP1 (left) and LARP6 (right) mRNA expression in HCC tissues and normal tissues in GEO databases. **D** LARP4B mRNA expression in HCC tissues and normal tissues in GEO databases. All results are presented as the mean ± SD. ****P*<0.001, *****P*<0.0001 and ns, no significance by a two-tailed Wilcoxon signed rank test for (B, D), a Mann-Whitney U test for (C).

**Fig. S2** LARP4B expression is upregulated by METTL3-mediated m^6^A-IGF2BP3-dependent modification. **A** Immunoblotting for LARP4B in Huh7 (left) and MHCC-97H (right) cells transfected with si“writers”. **B** Immunoblotting for METTL3 in HCC tissues and adjacent normal tissues. **C** Correlation between METTL3 and LARP4B mRNA levels in HCC tissues in GEO databases. **D** Immunoblotting for LARP4B in Huh7 cells transfected with si“readers”. **E** Correlation between IGF2BP3 and LARP4B mRNA levels in HCC tissues in GEO databases. All results are presented as the mean ± SD, and statistical significance was assessed using Pearson’s correlation analysis for (C, E).

**Fig. S3** LARP4B promotes stemness in HCC. **A** Overexpression and knockdown of LARP4B in SNU-449 cells were examined by western blot. **B, C** KEGG enrichment analyses of downexpressed proteins in Huh7 cells transfected with shNC or shLARP4B from MS data. **D, E** KEGG and GO enrichment analyses of downexpressed genes in Huh7 (D) or MHCC-97H (E) cells transfected with shNC or shLARP4B from RNA-seq data. **F** Correlation between LARP4B and EpCAM mRNA levels in HCC tissues in GEO database. **G** Flow cytometric analysis of EpCAM^+^, CD133^+^, and CD44^+^ cell populations in different groups of MHCC-97H cells. All results are presented as the mean ± SD. ***P*<0.01 and *****P*<0.0001 by a Pearson’s correlation analysis for (F), a two-tailed Student’s *t* test for (G).

**Fig. S4** LARP4B promotes HCC progression and LARP4B inhibition optimizes sorafenib efficacy. **A** Colony formation of different groups of SNU-449 cells treated with 0, 1, 5, or 10 µm sorafenib. **B** IHC staining of LARP4B in HCC tissues. Scale bars: 200 µm (left) and 50 µm (right). **C, D** Ex vivo bioluminescence imaging of lungs (left) and incidence of lung metastasis (right) in control vector and LARP4B group (C) and shNC and shLARP4B group (D). All results are presented as the mean ± SD. ****P*<0.001, *****P*<0.0001 and ns, no significance by a two-tailed Student’s *t* test.

**Fig. S5** LARP4B upregulates SPINK1 by binding to SPINK1 mRNA and maintaining its mRNA stability. **A** Volcanoplot shows the statistically upregulated and downregulated genes in Huh7 (left) and MHCC-97H (right) cells transfected with shNC or shLARP4B. **B** qPCR of ATP6V1FNB in Huh7 and MHCC-97H cells transfected with control vector or LARP4B. **C** SPINK1 mRNA expression in HCC tissues and normal liver tissues in GEO database. **D** Immunoblotting for SPINK1 in HCC tissues and adjacent normal tissues. **E** Correlation between LARP4B and SPINK1 mRNA levels in HCC tissues in GEO databases. **F** Elisa for SPINK1 in the conditioned media (CM) from different groups of Huh7 (left) and MHCC-97H (right) cells. All results are presented as the mean ± SD. ****P*<0.001, *****P*<0.0001 and ns, no significance by a Mann-Whitney U test for (B, C, F), a Pearson’s correlation analysis for (E).

**Fig. S6** LARP4B upregulates SPINK1 expression and promotes HCC progression in a La motif-dependent manner**. A** Immunoblotting for HA and SPINK1 in Huh7 (left) and MHCC-97H (right) cells transfected with control vector, LARP4B-WT, or LARP4B-ΔLM. IC, intracellular samples; CM, conditioned media. **B** Elisa for SPINK1 in the CM from different groups of Huh7 (left) and MHCC-97H (right) cells. **C** qPCR of SPINK1 in Huh7 (left) and MHCC-97H (right) cells transfected with control vector, LARP4B-WT, or LARP4B-ΔLM. **D** RIP-qPCR analysis of the enrichment of SPINK1 pre-mRNA on LARP4B in Huh7 (left) and MHCC-97H (right) cells transfected with control vector, LARP4B-WT, or LARP4B-ΔLM. **E**-**H** Sphere formation (E), proliferation (F), migration, invasion (G), and tube formation (H) of Huh7 (left) and MHCC-97H (right) cells transfected with control vector, LARP4B-WT, or LARP4B-ΔLM. Scale bars: 500 µm for (E), 200 µm for (G, H). All results are presented as the mean ± SD. ****P*<0.001, *****P*<0.0001 and ns, no significance by a Mann-Whitney U test for (B-D, F), a two-tailed Student’s *t* test for (E, G, H).

**Fig. S7** LARP4B promotes HCC progression and impairs sorafenib efficacy by activating SPINK1-mediated EGFR pathways. **A** Elisa for SPINK1 in the CM from different groups of Huh7 (left) and MHCC-97H (right) cells. **B** Sphere formation of different groups of MHCC-97H cells. Scale bars: 500 µm. **C** Flow cytometric analysis of EpCAM^+^, CD133^+^, and CD44^+^ cell populations in different groups of MHCC-97H cells. **D** Immunoblotting of the stemness-related proteins in different groups of different groups of MHCC-97H cells. **E** Proliferation of different groups of MHCC-97H cells treated with 0 or 5 µm sorafenib. **F** Colony formation of different groups of MHCC-97H cells treated with 0 or 5 µm sorafenib. **G** Invasion of different groups of MHCC-97H cells treated with 0 or 1 µm sorafenib. Scale bars: 100 µm. **H** Tube formation of different groups of and MHCC-97H cells. Scale bars: 200 µm. **I** Ex vivo bioluminescence imaging of lungs (left) and incidence of lung metastasis (right) in different groups treated with 0, 10 or 30 mg/kg sorafenib. **J** Immunoblotting of indicated proteins and phosphorylation levels in different groups of MHCC-97H cells. All results are presented as the mean ± SD. ***P*<0.01, ****P*<0.001, *****P*<0.0001 and ns, no significance by a two-tailed Student’s *t* test for (B, C, F-H), a Mann-Whitney U test for (A, E).

**Fig. S8** A positive feedback loop with the LARP4B/SPINK1/p-AKT/C/EBP-β axis was identified. **A** Immunoblotting for LARP4B and METTL3 in Huh7 (left) and MHCC-97H (right) cells treated with DMSO, BAY11, AG490, U0126, or LY294002. **B** Immunoblotting for p-AKT and AKT in Huh7 (upper) and MHCC-97H (bottom) cells treated with DMSO, 1, 5, or 10 µm sorafenib.

**Table S1.** Correlation between LARP4B expression and clinicopathological features of HCC

| Variables |  | LARP4B expression | | P value |
| --- | --- | --- | --- | --- |
|  |  | Low (n=32) | High (n=37) |  |
| Gender | Male | 26 | 32 | 0.553 |
|  | female | 6 | 5 |  |
| Age | > 50 | 12 | 15 | 0.165 |
|  | ≤ 50 | 20 | 12 |  |
| HBV | Positive | 26 | 29 | 0.767 |
|  | negative | 6 | 8 |  |
| Tumor size | > 5 cm | 18 | 18 | 0.528 |
|  | ≤ 5 cm | 14 | 19 |  |
| Microvascular involvement | positive | 10 | 14 | 0.567 |
|  | negative | 22 | 23 |  |
| Differentiation | poorly | 9 | 13 | 0.533 |
|  | well/ moderately | 23 | 24 |  |
| TNM stage | Ⅲ-Ⅳ | 3 | 10 | 0.021* |
|  | Ⅰ-Ⅱ | 29 | 20 |  |
| AFP | > 20 ng/ml | 10 | 21 | 0.034* |
|  | ≤ 20 ng/ml | 22 | 16 |  |

* *P*<0.05

**Table S2.** Antibodies used for immunohistochemistry (IHC) staining, Western blot (WB), IP, and FC

| Antigen name | Commercial source | Catalog number | Application | Dilution time |
| --- | --- | --- | --- | --- |
| LARP4B | Invitrogen | PA5-57721 | WB, IHC | 0.4µg/ml, 1:200 |
| LARP4B | LSBio | LS-C762142 | RIP | 5µg |
| METTL3 | Abcam | ab195352 | WB, RIP | 1:1000, 5µg |
| IGF2BP3 | Abcam | ab177477 | WB, RIP | 1:1000, 5µg |
| CD133 | Abcam | ab222782 | WB | 1:2000 |
| CD44 | CST | 37259S | WB | 1:1000 |
| EpCAM | Proteintech | 66316-1-Ig | WB | 1:2000 |
| Nanog | Abcam | ab109250 | WB | 1:1000 |
| SOX2 | Abcam | ab92494 | WB | 1:1000 |
| C-MYC | Abcam | ab32072 | WB | 1:1000 |
| Epcam | Invitrogen | 46-9326-42 | FC | 0.06µg/test |
| CD133 | Invitrogen | 62-1338-42 | FC | 0.25µg/test |
| CD44 | Invitrogen | 25-0441-82 | FC | 0.125µg/test |
| Vimentin | Proteintech | 10366-1-AP | WB | 1:2000 |
| N-cadherin | CST | 13116S | WB | 1:1000 |
| CD31 | CST | 3528S | WB, IHC | 1:1000, 1:800 |
| CD34 | Invitrogen | PA5-85917 | WB, IHC | 1:1000, 1:2000 |
| Ki67 | Abcam | Ab15580 | IHC | 1:500 |
| SPINK1 | Abnova | H00006690-M01 | WB, IHC | 1:500, 1:200 |
| HA | Abcam | Ab236632 | WB | 1:1000 |
| P-EGFR | CST | 3777S | WB | 1:1000 |
| EGFR | Proteintech | 66455-1-Ig | WB | 1:5000 |
| P-ERK1/2 | CST | 4370S | WB | 1:1000 |
| ERK1/2 | Proteintech | 11257-1-AP | WB | 1:2000 |
| P-AKT | CST | 4060S | WB | 1:1000 |
| AKT | Proteintech | 60203-2-Ig | WB | 1:5000 |
| P-STAT3 | CST | 9145S | WB | 1:1000 |
| STAT3 | Proteintech | 60199-1-Ig | WB | 1:2000 |
| P-mTOR | CST | 5536S | WB | 1:1000 |
| mTOR | Proteintech | 66888-1-Ig | WB | 1:5000 |
| C/EBP-β | Abcam | Ab32358 | WB, CHIP | 1:1000, 1:30 |
| GAPDH | Proteintech | 60004-1-Ig | WB | 1:10000 |

**Table S3.** Primers used for qPCR, and CHIP

| Target name | Forward (5’-3’) | Reverse (5’-3’) | Application |
| --- | --- | --- | --- |
| LARP4B | tcgggacgctaaggttgtgg | tcagatgagcgctgtccttg | qPCR |
| SPINK1 | gtgcggtgcagttttca | ttccatcagtcccacagac | qPCR |
| ATP6V1FNB | actgtccacttgctatgccc | ccttccagaagttttgccgc | qPCR |
| β-actin | catgtacgttgctatccaggc | ctccttaatgtcacgcacgat | qPCR |
| LARP4B | gttcagggcgcgaggc | cggggacggcgaggaga | CHIP |
