# Supplementary material for: LARP4B promotes hepatocellular carcinoma progression and impairs sorafenib efficacy by activating SPINK1-mediated EGFR pathway
Source: Cell Death Discov. 2024 May 1;10:208. doi: 10.1038/s41420-024-01985-6 (PMC11063073; doi:10.1038/s41420-024-01985-6)
Supplement: Supplementary file 10 — Supplementary file 2 [file 41420_2024_1985_MOESM10_ESM.pdf]

Supplementary file 2

LARP4B promotes hepatocellular carcinoma progression and impairs sorafenib efficacy by activating SPINK1-mediated EGFR pathway

The original, full-length gel and blot images

Fig. 1

C

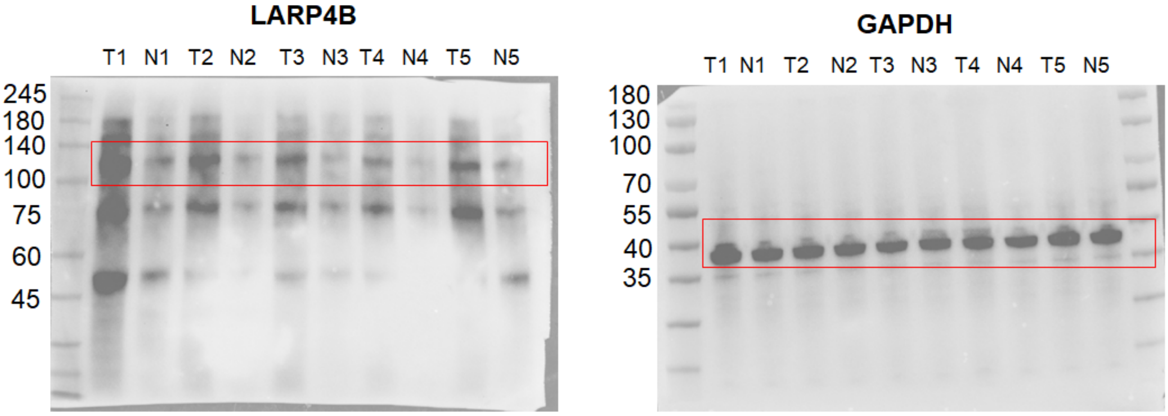

Fig. 2

C

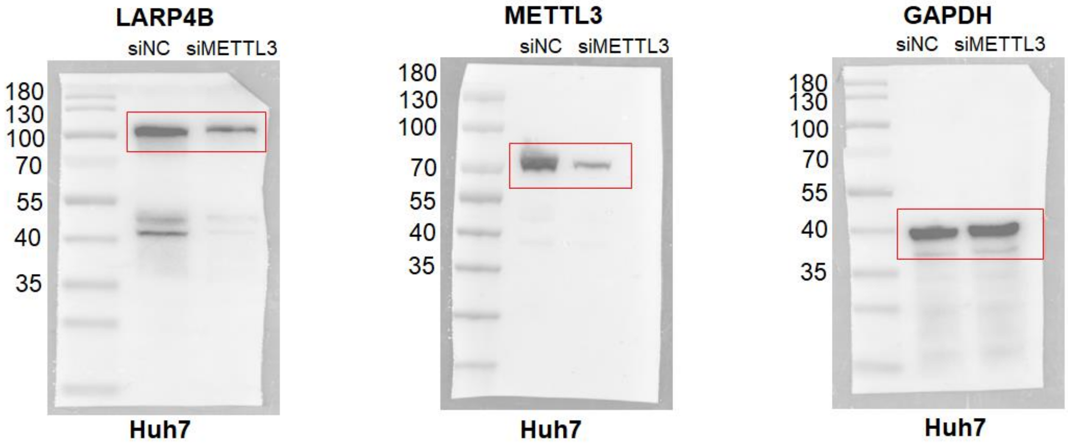

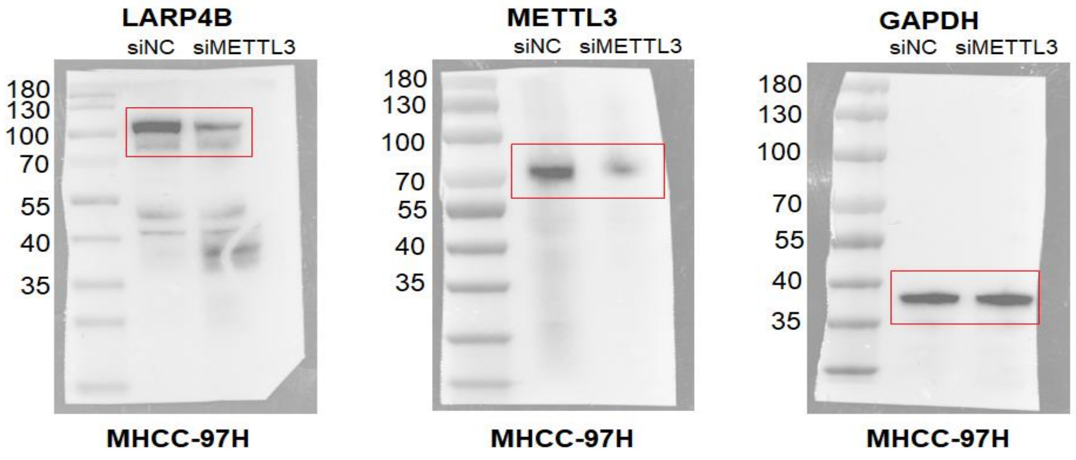
LARP4B
siNC siMETTL3
180
130
100
70
55
40
35
MHCC-97H
METTL3
siNC siMETTL3
180
130
100
70
55
40
35
MHCC-97H
GAPDH
siNC siMETTL3
180
130
100
70
55
40
35
MHCC-97H

G

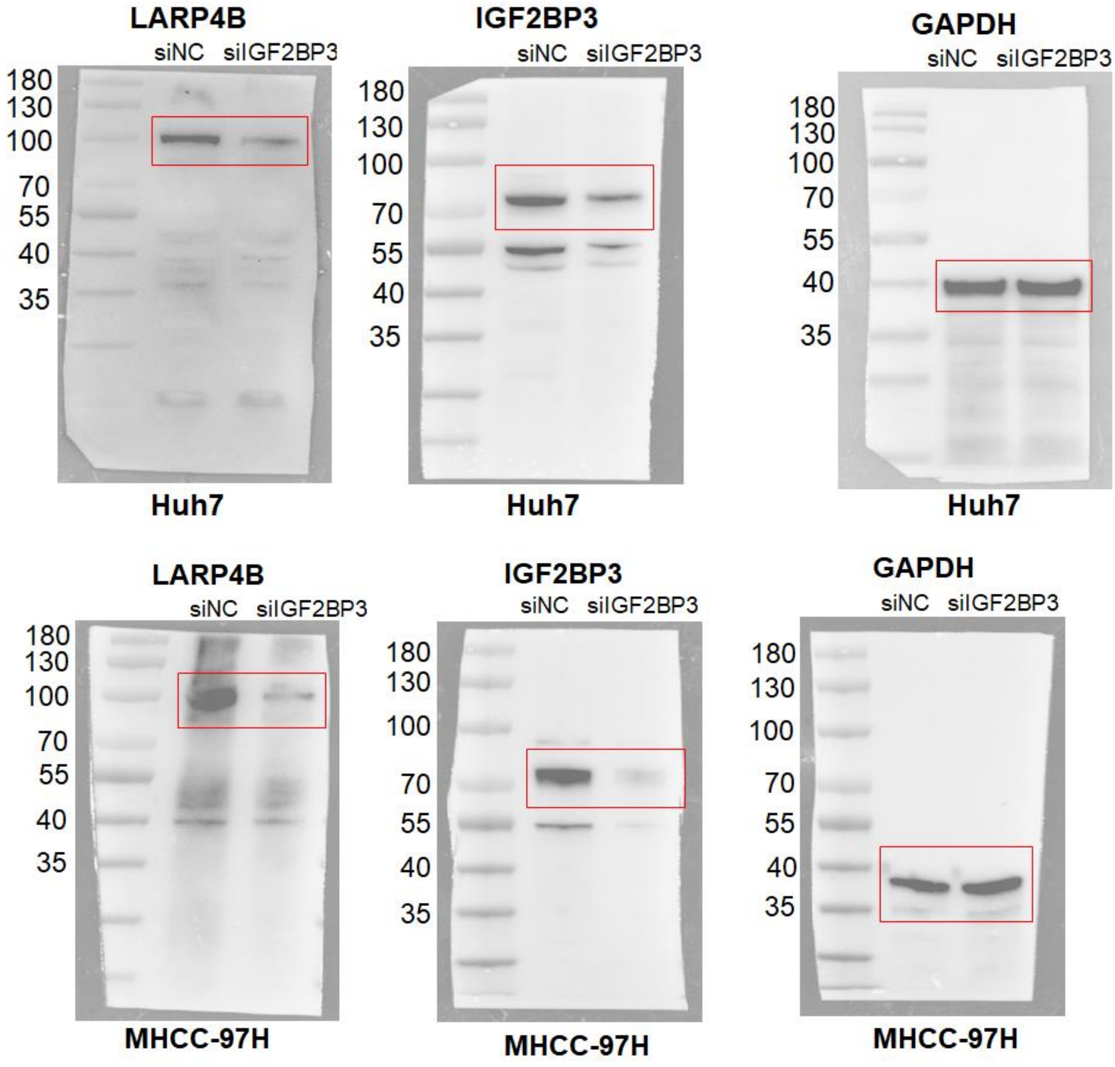
LARP4B
siNC siIGF2BP3
180
130
100
70
55
40
35
Huh7
IGF2BP3
siNC siIGF2BP3
180
130
100
70
55
40
35
Huh7
GAPDH
siNC siIGF2BP3
180
130
100
70
55
40
35
Huh7
LARP4B
siNC siIGF2BP3
180
130
100
70
55
40
35
MHCC-97H
IGF2BP3
siNC siIGF2BP3
180
130
100
70
55
40
35
MHCC-97H
GAPDH
siNC siIGF2BP3
180
130
100
70
55
40
35
MHCC-97H

I

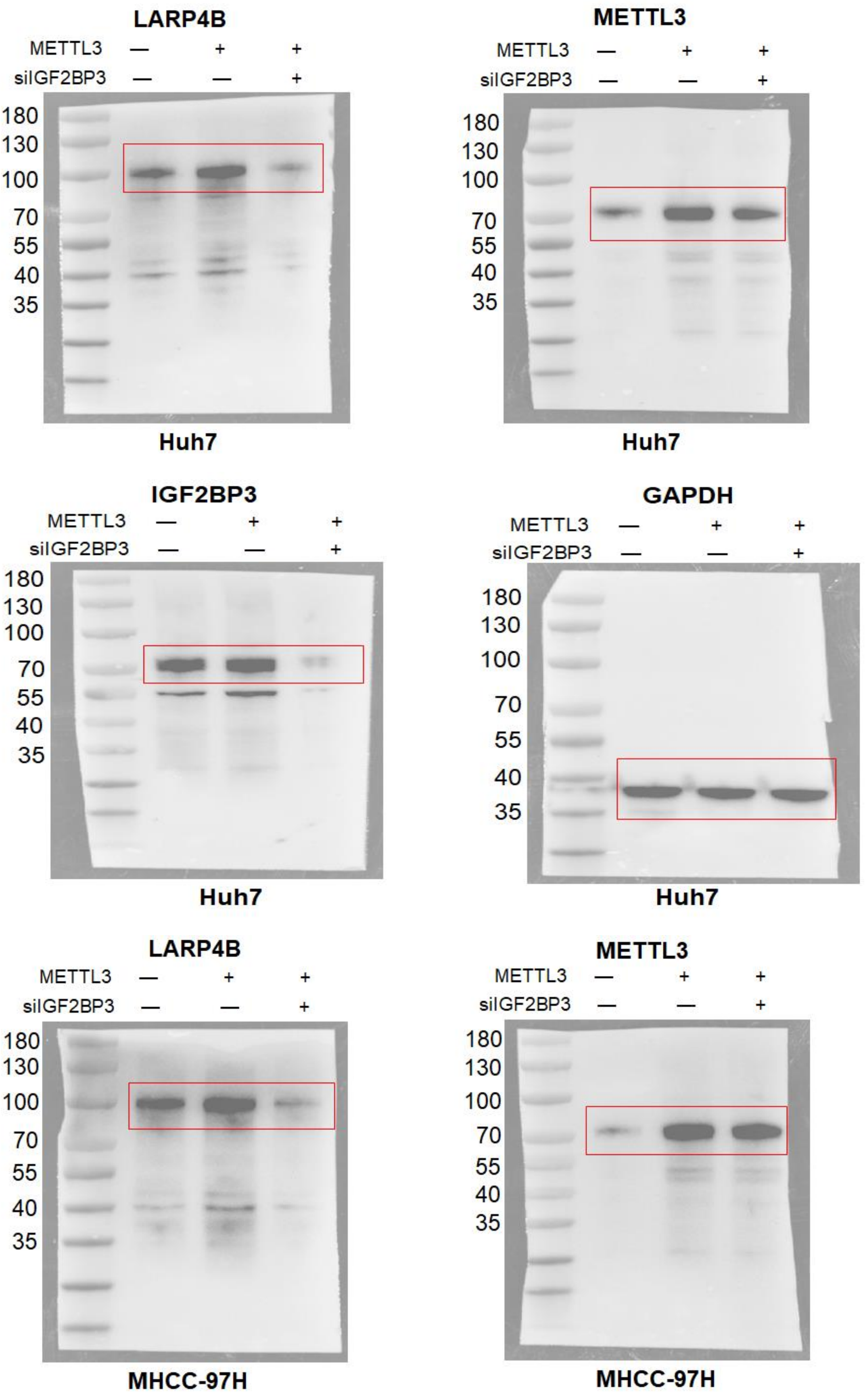

LARP4B
METTL3 — + +
siIGF2BP3 — — +
180
130
100
70
55
40
35
Huh7
METTL3
METTL3 — + +
siIGF2BP3 — — +
180
130
100
70
55
40
35
Huh7
IGF2BP3
METTL3 — + +
siIGF2BP3 — — +
180
130
100
70
55
40
35
Huh7
GAPDH
METTL3 — + +
siIGF2BP3 — — +
180
130
100
70
55
40
35
Huh7
LARP4B
METTL3 — + +
siIGF2BP3 — — +
180
130
100
70
55
40
35
MHCC-97H
METTL3
METTL3 — + +
siIGF2BP3 — — +
180
130
100
70
55
40
35
MHCC-97H

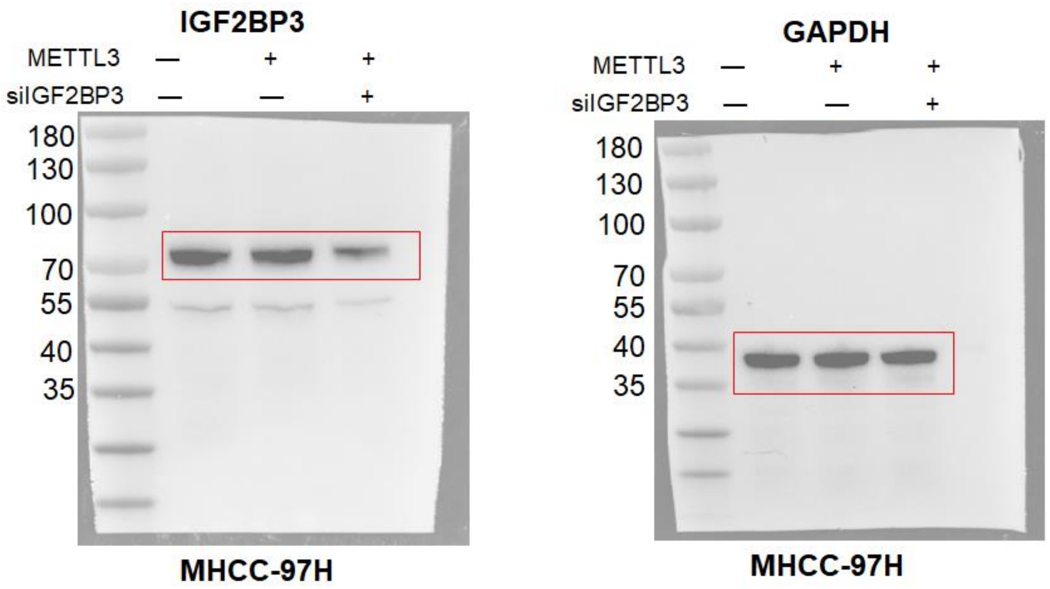
IGF2BP3
METTL3 — + +
siIGF2BP3 — — +
180
130
100
70
55
40
35
MHCC-97H
GAPDH
METTL3 — + +
siIGF2BP3 — — +
180
130
100
70
55
40
35
MHCC-97H

Fig. 3

A

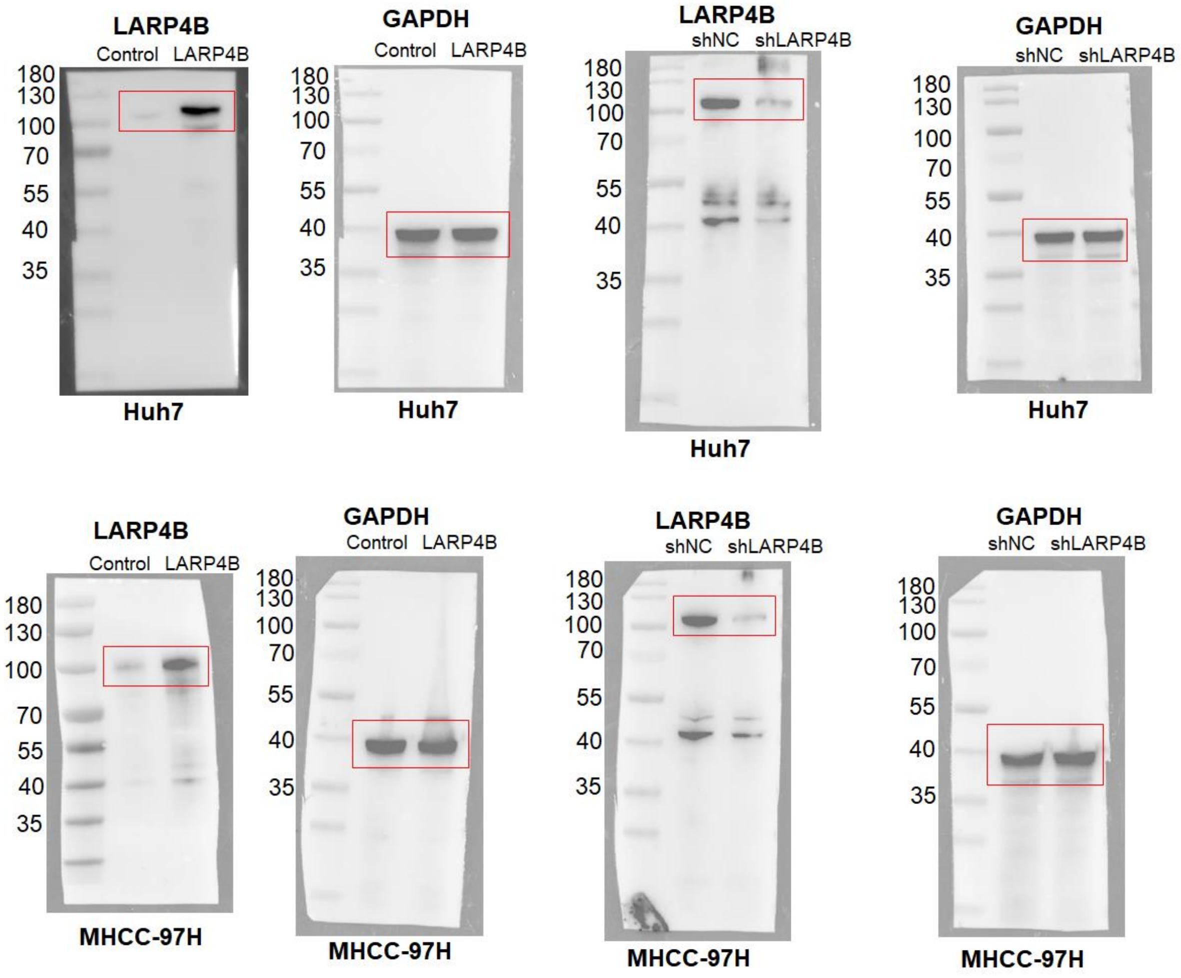
LARP4B
Control LARP4B
Huh7
GAPDH
Control LARP4B
Huh7
LARP4B
shNC shLARP4B
Huh7
GAPDH
shNC shLARP4B
Huh7
180
130
100
70
55
40
35
LARP4B
Control LARP4B
MHCC-97H
GAPDH
Control LARP4B
MHCC-97H
LARP4B
shNC shLARP4B
MHCC-97H
GAPDH
shNC shLARP4B
MHCC-97H

C

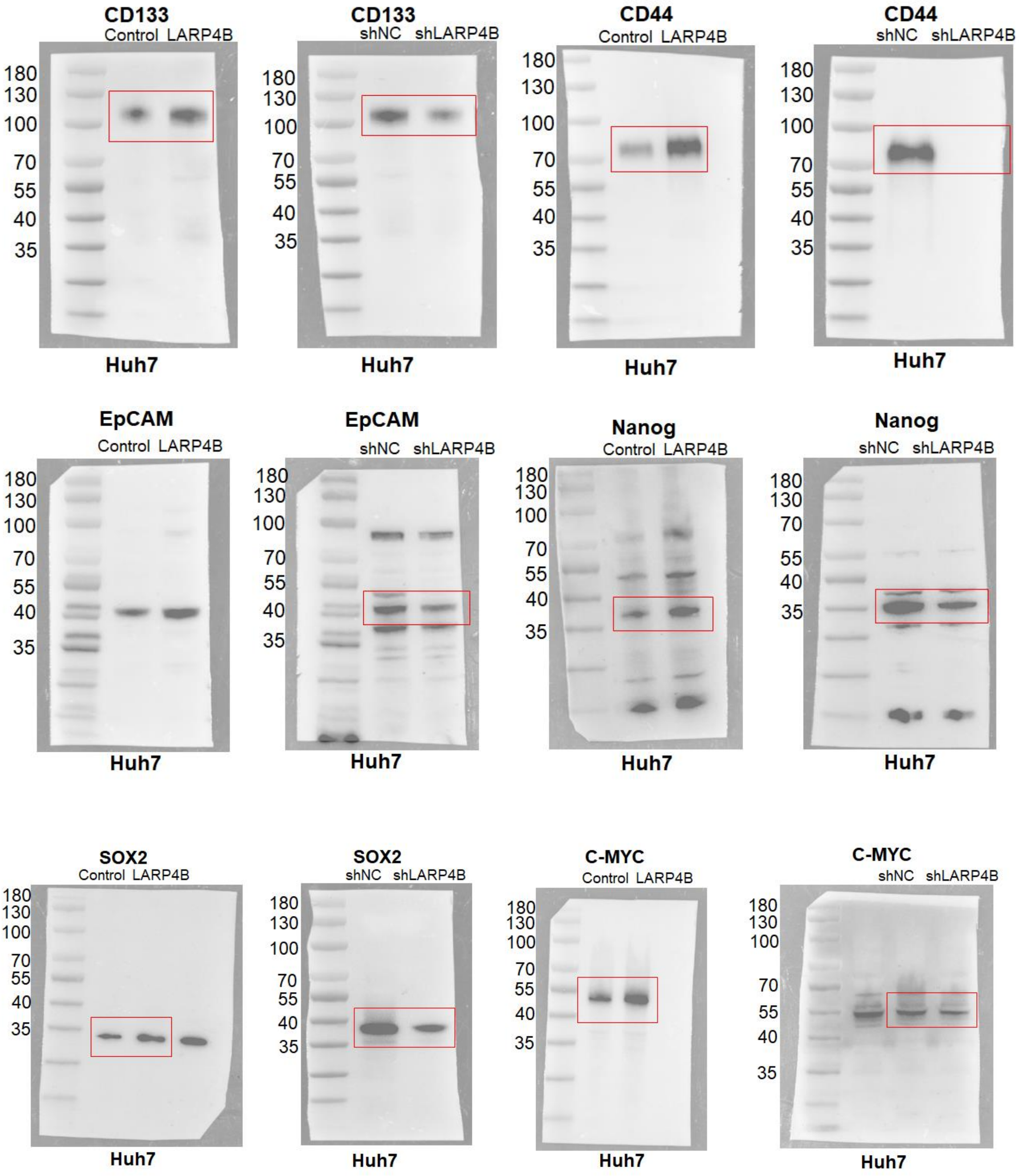
CD133
Control LARP4B
Huh7
CD133
shNC shLARP4B
Huh7
CD44
Control LARP4B
Huh7
CD44
shNC shLARP4B
Huh7
EpCAM
Control LARP4B
Huh7
EpCAM
shNC shLARP4B
Huh7
Nanog
Control LARP4B
Huh7
Nanog
shNC shLARP4B
Huh7
SOX2
Control LARP4B
Huh7
SOX2
shNC shLARP4B
Huh7
C-MYC
Control LARP4B
Huh7
C-MYC
shNC shLARP4B
Huh7
180
130
100
70
55
40
35

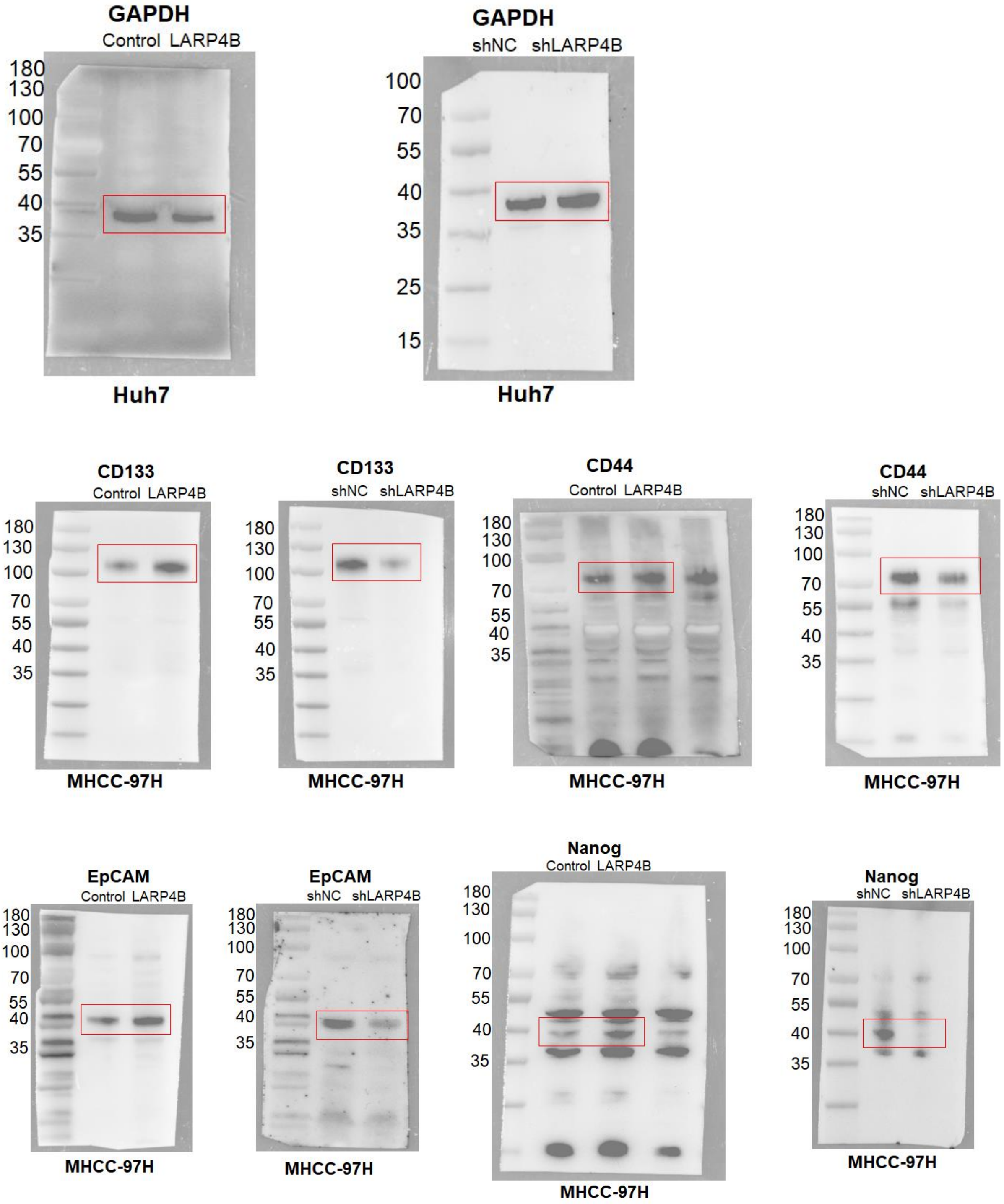

GAPDH
Control LARP4B
180 130 100 70 55 40 35
Huh7
GAPDH
shNC shLARP4B
100 70 55 40 35 25 15
Huh7
CD133
Control LARP4B
180 130 100 70 55 40 35
MHCC-97H
CD133
shNC shLARP4B
180 130 100 70 55 40 35
MHCC-97H
CD44
Control LARP4B
180 130 100 70 55 40 35
MHCC-97H
CD44
shNC shLARP4B
180 130 100 70 55 40 35
MHCC-97H
EpCAM
Control LARP4B
180 130 100 70 55 40 35
MHCC-97H
EpCAM
shNC shLARP4B
180 130 100 70 55 40 35
MHCC-97H
Nanog
Control LARP4B
180 130 100 70 55 40 35
MHCC-97H
Nanog
shNC shLARP4B
180 130 100 70 55 40 35
MHCC-97H

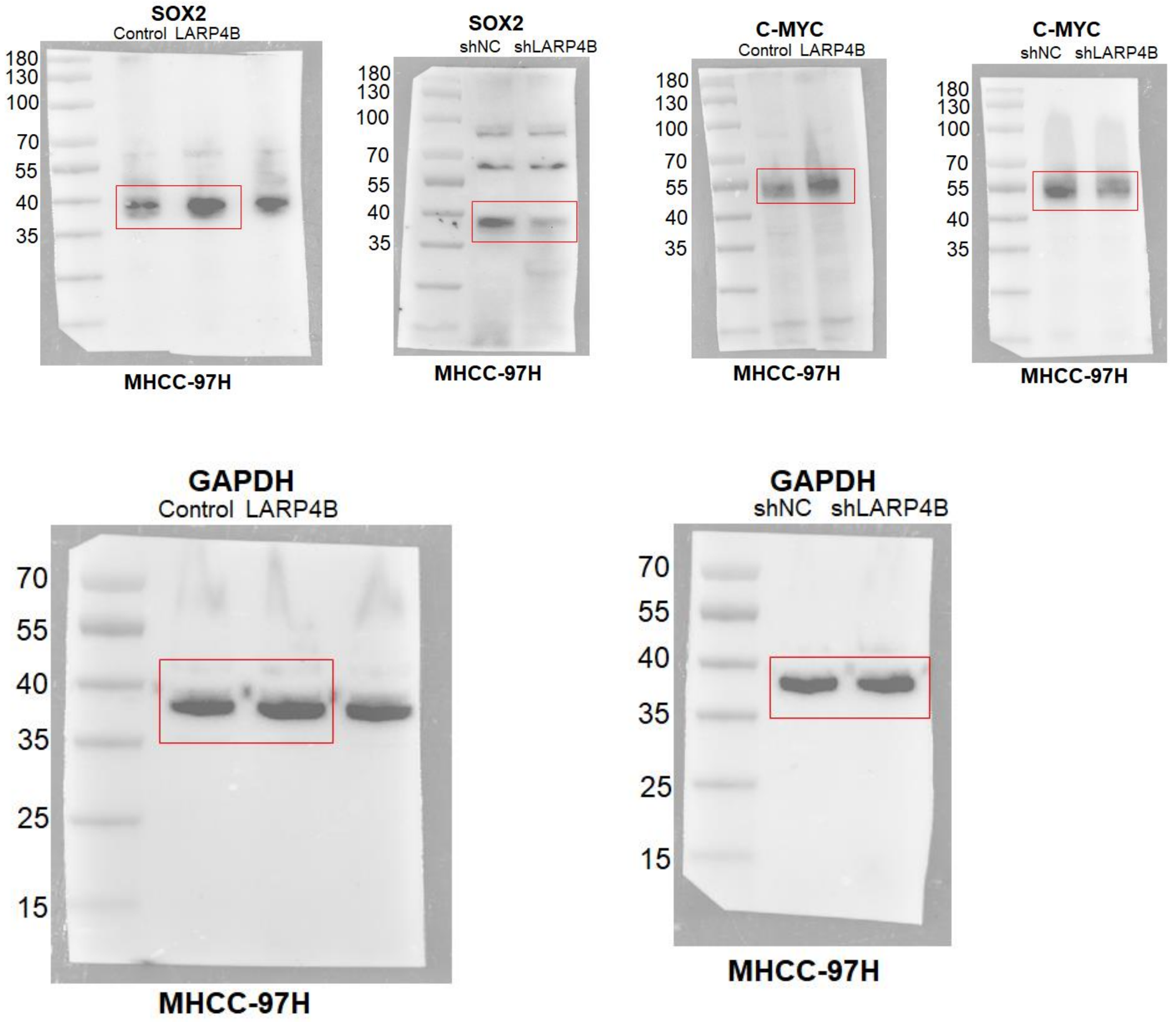
SOX2
Control LARP4B
180
130
100
70
55
40
35
MHCC-97H
SOX2
shNC shLARP4B
MHCC-97H
C-MYC
Control LARP4B
MHCC-97H
C-MYC
shNC shLARP4B
MHCC-97H
GAPDH
Control LARP4B
70
55
40
35
25
15
MHCC-97H
GAPDH
shNC shLARP4B
MHCC-97H

E

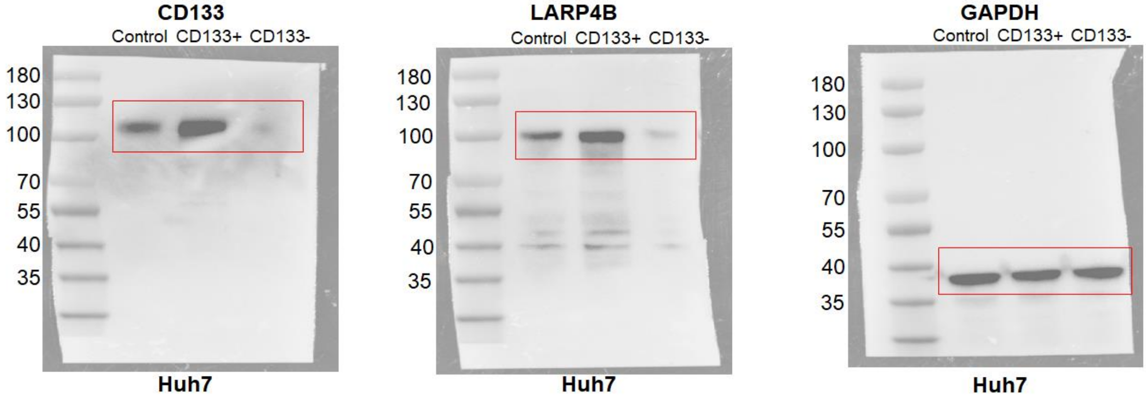
CD133
Control CD133+ CD133-
180
130
100
70
55
40
35
Huh7
LARP4B
Control CD133+ CD133-
Huh7
GAPDH
Control CD133+ CD133-
Huh7

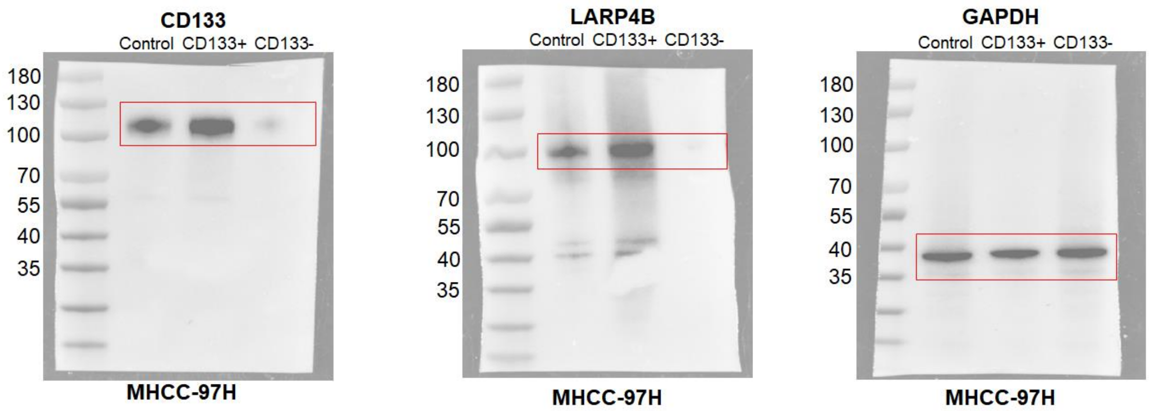
CD133
Control CD133+ CD133-
180
130
100
70
55
40
35
MHCC-97H
LARP4B
Control CD133+ CD133-
180
130
100
70
55
40
35
MHCC-97H
GAPDH
Control CD133+ CD133-
180
130
100
70
55
40
35
MHCC-97H

Fig. 4

F

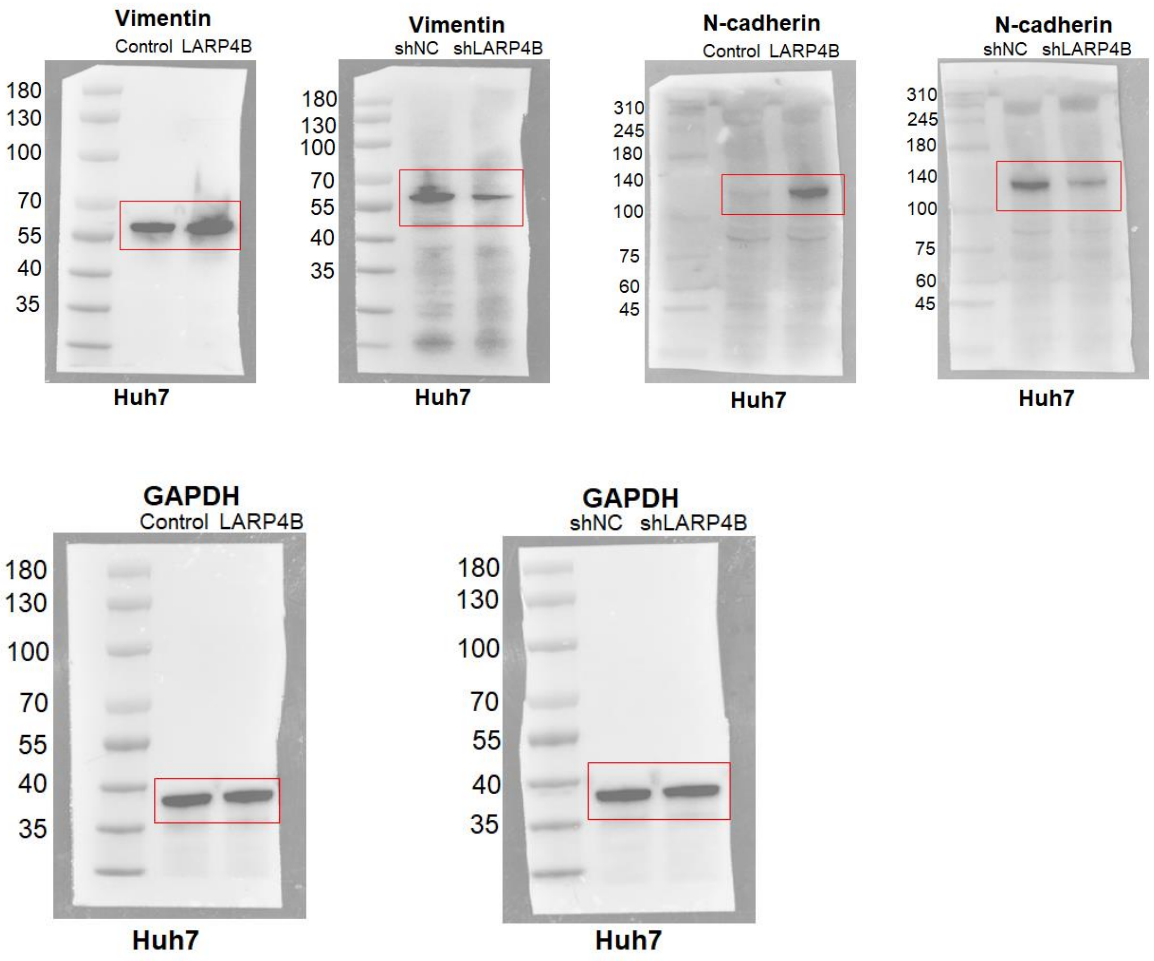
Vimentin
Control LARP4B
180
130
100
70
55
40
35
Huh7
Vimentin
shNC shLARP4B
180
130
100
70
55
40
35
Huh7
N-cadherin
Control LARP4B
310
245
180
140
100
75
60
45
Huh7
N-cadherin
shNC shLARP4B
310
245
180
140
100
75
60
45
Huh7
GAPDH
Control LARP4B
180
130
100
70
55
40
35
Huh7
GAPDH
shNC shLARP4B
180
130
100
70
55
40
35
Huh7

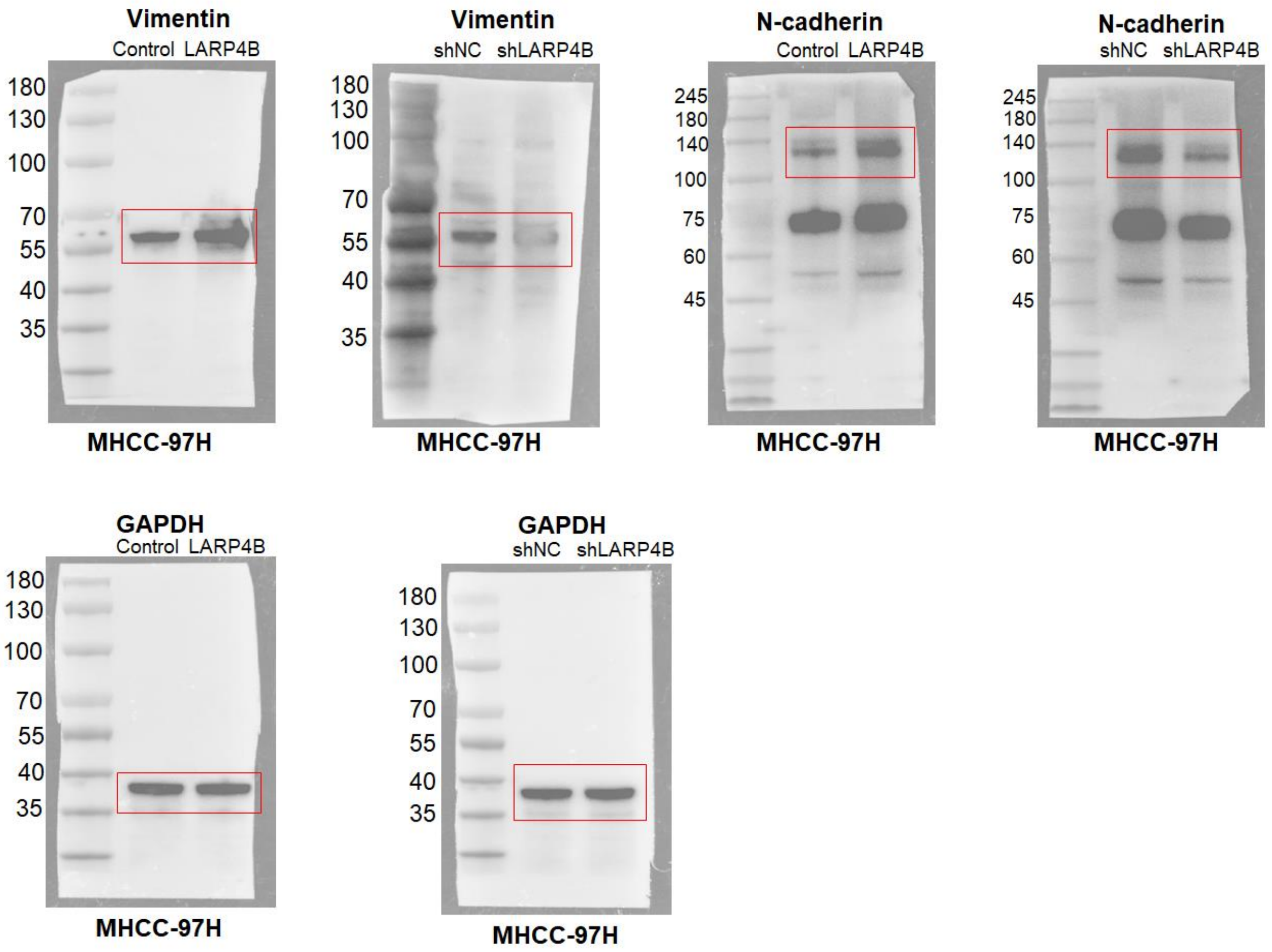
Vimentin
Control LARP4B
180
130
100
70
55
40
35
MHCC-97H
Vimentin
shNC shLARP4B
180
130
100
70
55
40
35
MHCC-97H
N-cadherin
Control LARP4B
245
180
140
100
75
60
45
MHCC-97H
N-cadherin
shNC shLARP4B
245
180
140
100
75
60
45
MHCC-97H
GAPDH
Control LARP4B
180
130
100
70
55
40
35
MHCC-97H
GAPDH
shNC shLARP4B
180
130
100
70
55
40
35
MHCC-97H

H

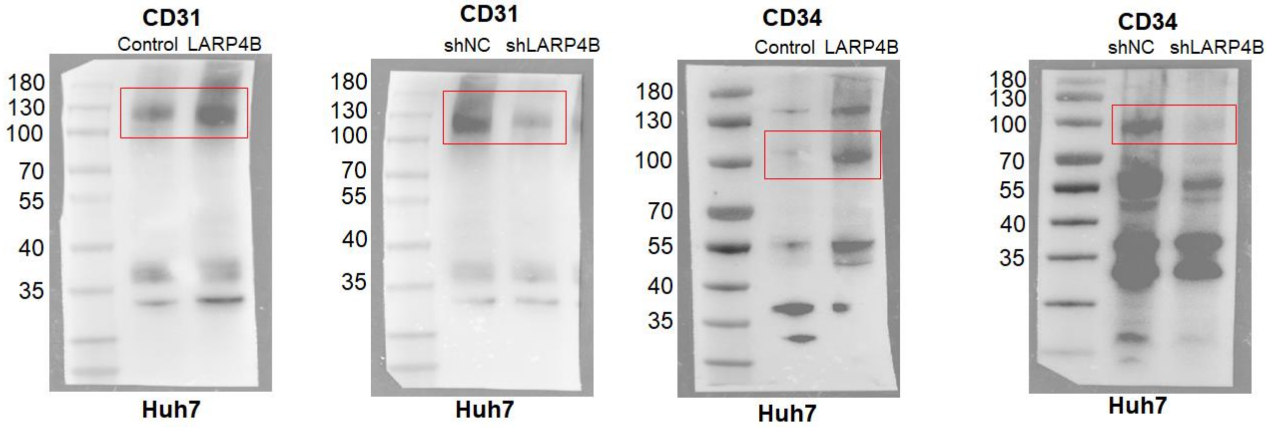
CD31
Control LARP4B
180
130
100
70
55
40
35
Huh7
CD31
shNC shLARP4B
180
130
100
70
55
40
35
Huh7
CD34
Control LARP4B
180
130
100
70
55
40
35
Huh7
CD34
shNC shLARP4B
180
130
100
70
55
40
35
Huh7

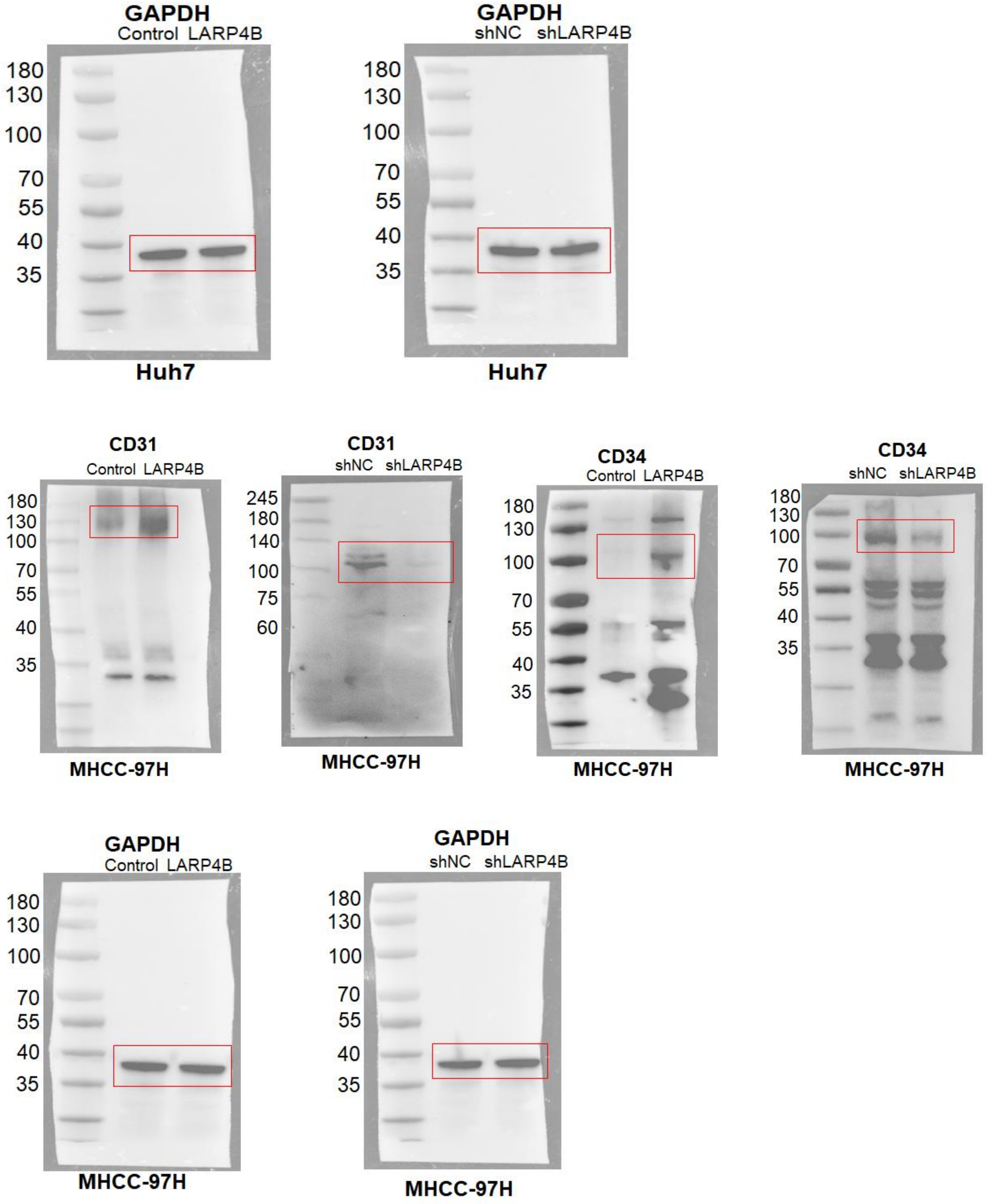
GAPDH
Control LARP4B
180
130
100
70
55
40
35
Huh7
GAPDH
shNC shLARP4B
180
130
100
70
55
40
35
Huh7
CD31
Control LARP4B
180
130
100
70
55
40
35
MHCC-97H
CD31
shNC shLARP4B
245
180
140
100
75
60
MHCC-97H
CD34
Control LARP4B
180
130
100
70
55
40
35
MHCC-97H
CD34
shNC shLARP4B
180
130
100
70
55
40
35
MHCC-97H
GAPDH
Control LARP4B
180
130
100
70
55
40
35
MHCC-97H
GAPDH
shNC shLARP4B
180
130
100
70
55
40
35
MHCC-97H

Fig. 6

F

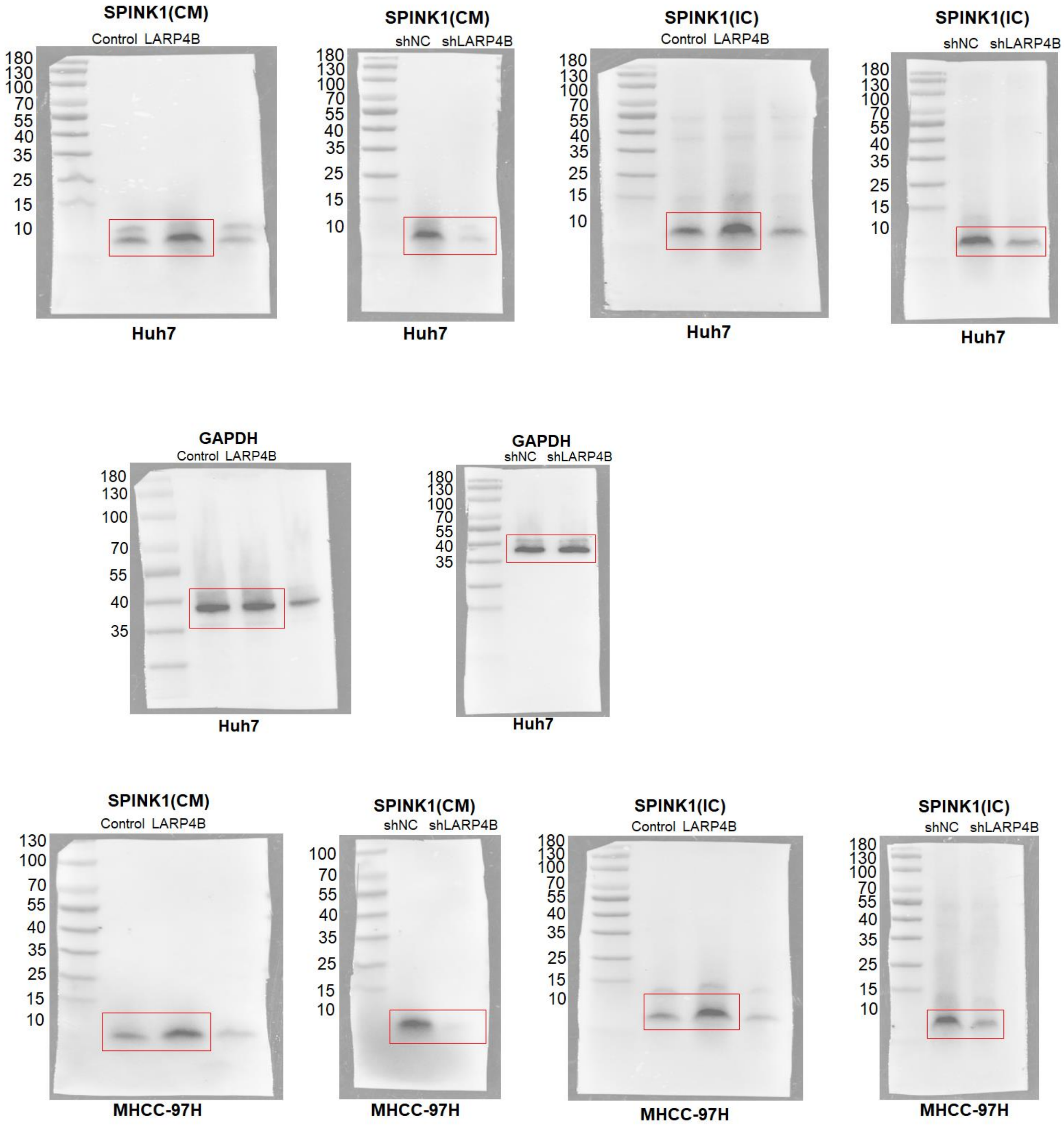

SPINK1(CM)
Control LARP4B
180
130
100
70
55
40
35
25
15
10
Huh7
SPINK1(CM)
shNC shLARP4B
180
130
100
70
55
40
35
25
15
10
Huh7
SPINK1(IC)
Control LARP4B
180
130
100
70
55
40
35
25
15
10
Huh7
SPINK1(IC)
shNC shLARP4B
180
130
100
70
55
40
35
25
15
10
Huh7
GAPDH
Control LARP4B
180
130
100
70
55
40
35
Huh7
GAPDH
shNC shLARP4B
180
130
100
70
55
40
35
Huh7
SPINK1(CM)
Control LARP4B
130
100
70
55
40
35
25
15
10
MHCC-97H
SPINK1(CM)
shNC shLARP4B
100
70
55
40
35
25
15
10
MHCC-97H
SPINK1(IC)
Control LARP4B
180
130
100
70
55
40
35
25
15
10
MHCC-97H
SPINK1(IC)
shNC shLARP4B
180
130
100
70
55
40
35
25
15
10
MHCC-97H

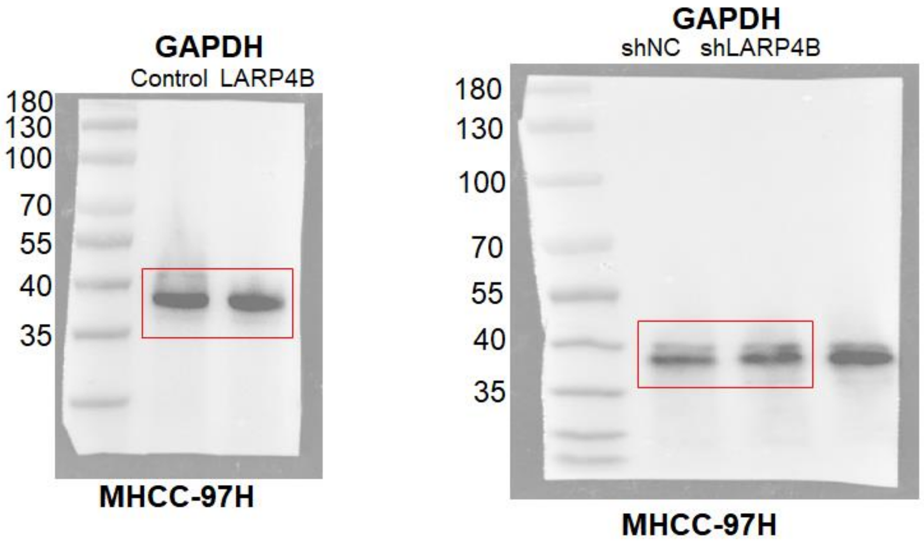
GAPDH
Control LARP4B
180
130
100
70
55
40
35
MHCC-97H
GAPDH
shNC shLARP4B
180
130
100
70
55
40
35
MHCC-97H

Fig. 7

A

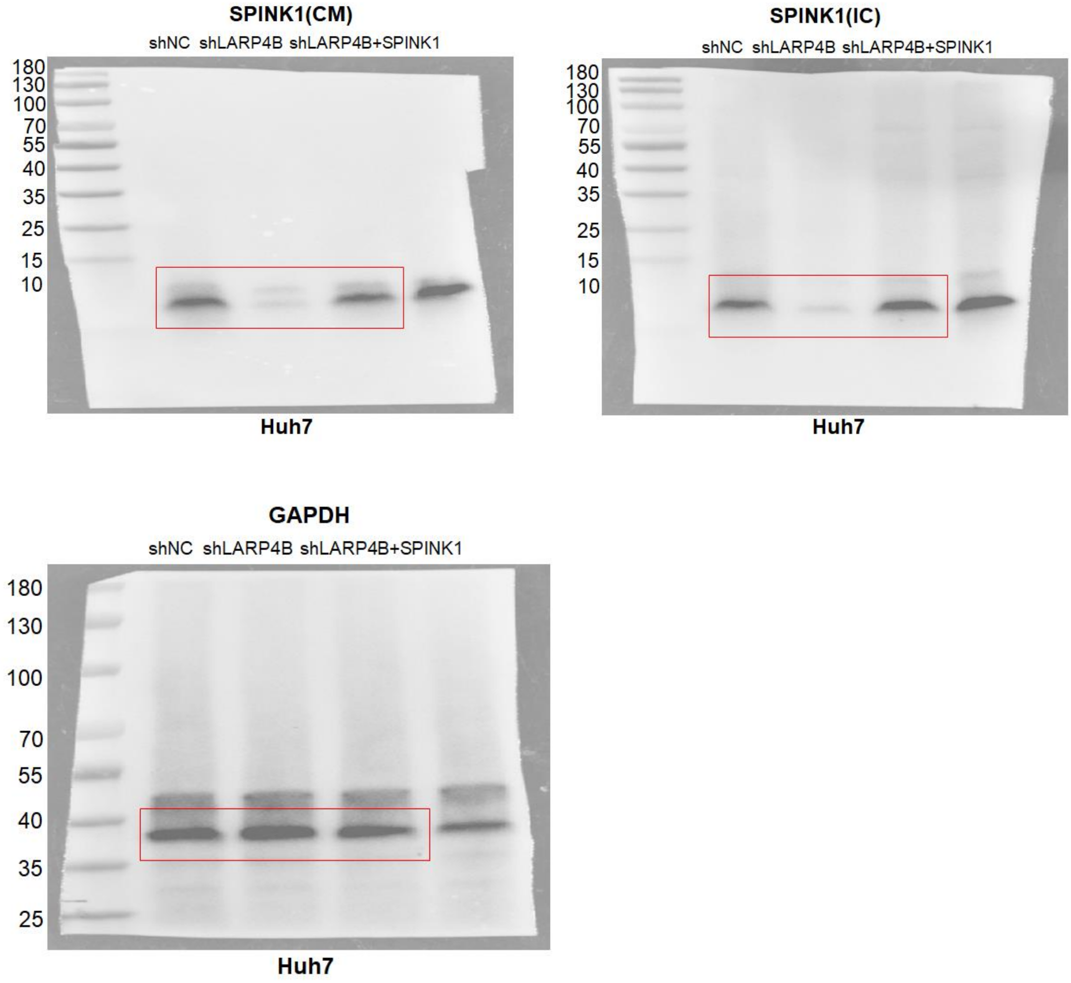
SPINK1(CM)
shNC shLARP4B shLARP4B+SPINK1
180
130
100
70
55
40
35
25
15
10
Huh7
SPINK1(IC)
shNC shLARP4B shLARP4B+SPINK1
180
130
100
70
55
40
35
25
15
10
Huh7
GAPDH
shNC shLARP4B shLARP4B+SPINK1
180
130
100
70
55
40
35
25
Huh7

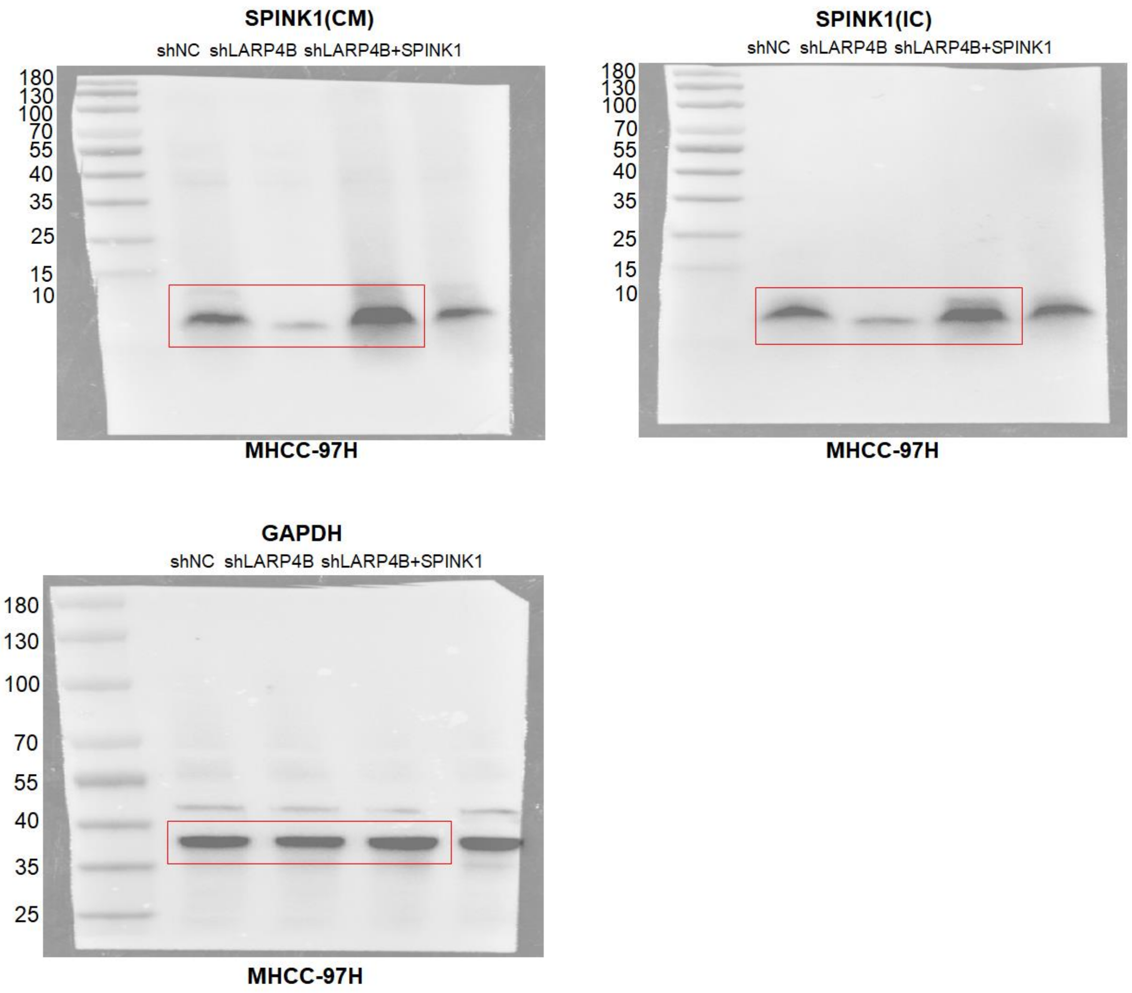
SPINK1(CM)
shNC shLARP4B shLARP4B+SPINK1
180 130 100 70 55 40 35 25 15 10
MHCC-97H
SPINK1(IC)
shNC shLARP4B shLARP4B+SPINK1
180 130 100 70 55 40 35 25 15 10
MHCC-97H
GAPDH
shNC shLARP4B shLARP4B+SPINK1
180 130 100 70 55 40 35 25
MHCC-97H

D

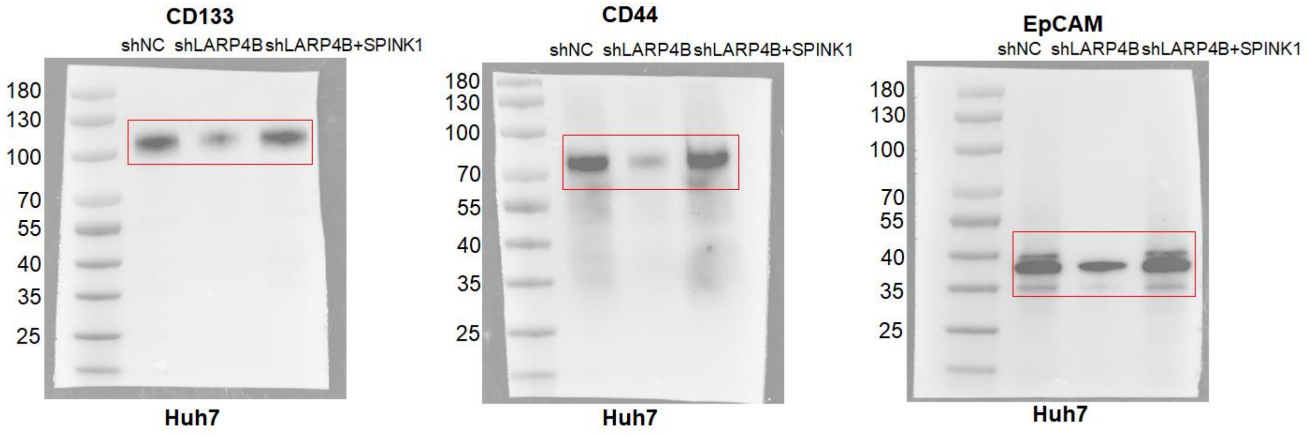
CD133
shNC shLARP4B shLARP4B+SPINK1
180 130 100 70 55 40 35 25
Huh7
CD44
shNC shLARP4B shLARP4B+SPINK1
180 130 100 70 55 40 35 25
Huh7
EpCAM
shNC shLARP4B shLARP4B+SPINK1
180 130 100 70 55 40 35 25
Huh7

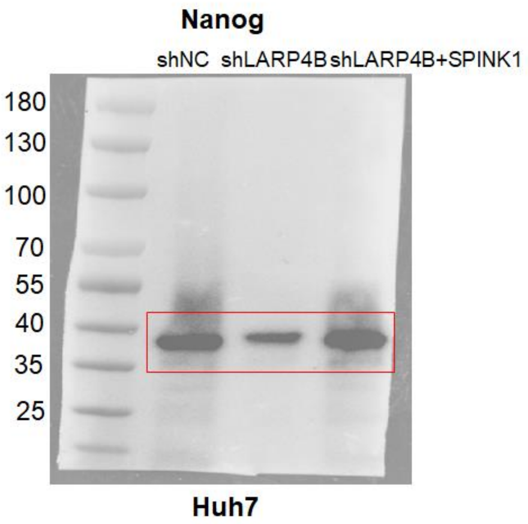
Nanog
shNC shLARP4B shLARP4B+SPINK1
180
130
100
70
55
40
35
25
Huh7

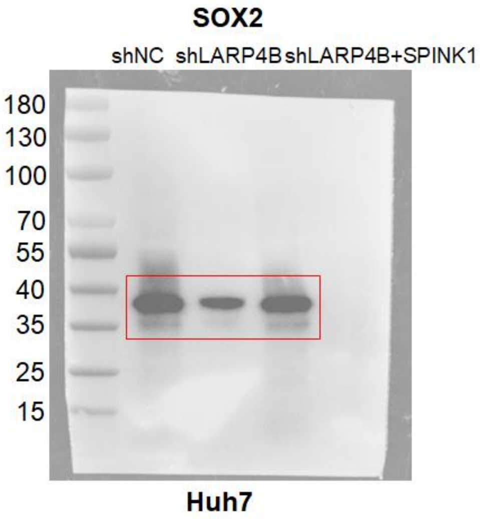
SOX2
shNC shLARP4B shLARP4B+SPINK1
180
130
100
70
55
40
35
25
15
Huh7

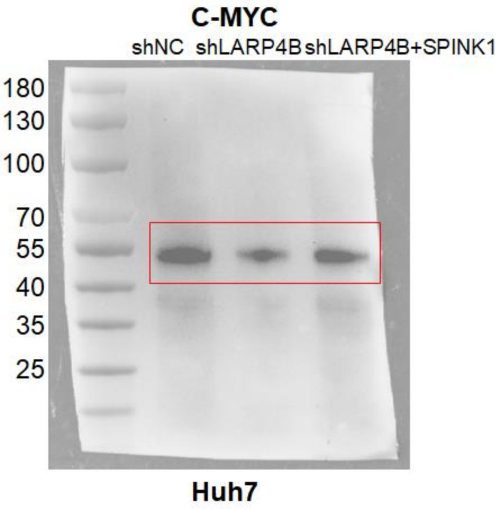
C-MYC
shNC shLARP4B shLARP4B+SPINK1
180
130
100
70
55
40
35
25
Huh7

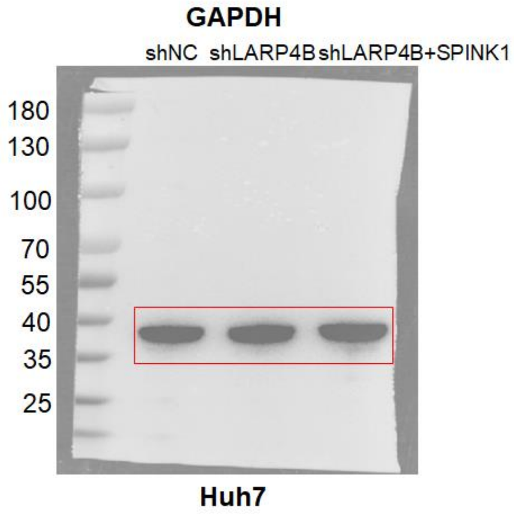
GAPDH
shNC shLARP4B shLARP4B+SPINK1
180
130
100
70
55
40
35
25
Huh7

H

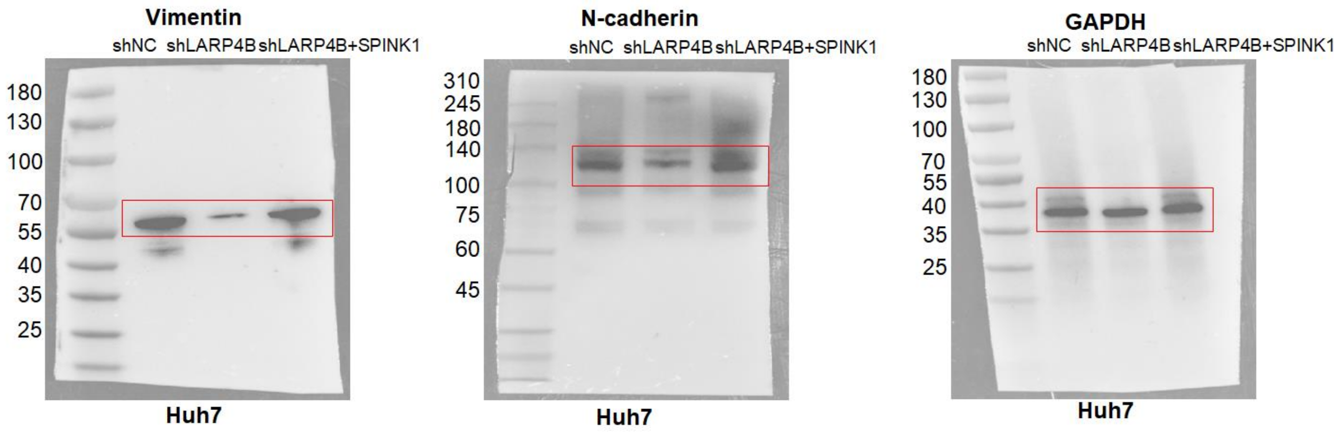
Vimentin
shNC shLARP4B shLARP4B+SPINK1
180
130
100
70
55
40
35
25
Huh7
N-cadherin
shNC shLARP4B shLARP4B+SPINK1
310
245
180
140
100
75
60
45
Huh7
GAPDH
shNC shLARP4B shLARP4B+SPINK1
180
130
100
70
55
40
35
25
Huh7

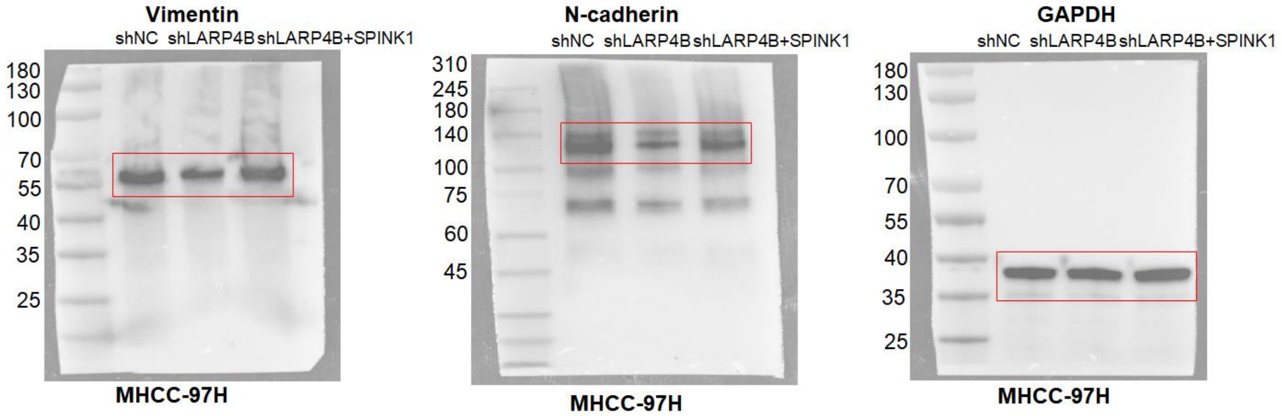
Vimentin
shNC shLARP4B shLARP4B+SPINK1
180 130 100 70 55 40 35 25
MHCC-97H
N-cadherin
shNC shLARP4B shLARP4B+SPINK1
310 245 180 140 100 75 60 45
MHCC-97H
GAPDH
shNC shLARP4B shLARP4B+SPINK1
180 130 100 70 55 40 35 25
MHCC-97H

J

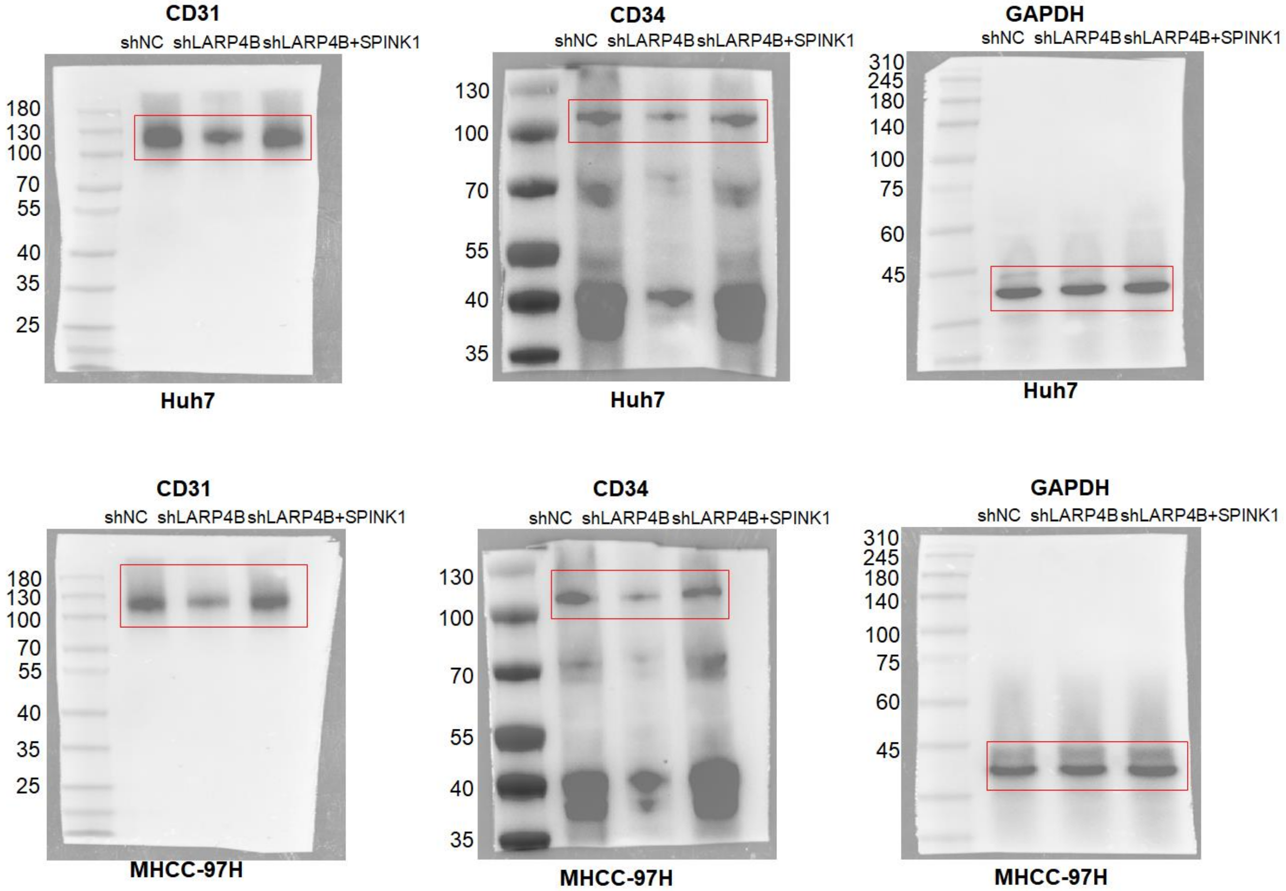
CD31
shNC shLARP4B shLARP4B+SPINK1
180 130 100 70 55 40 35 25
Huh7
CD34
shNC shLARP4B shLARP4B+SPINK1
130 100 70 55 40 35
Huh7
GAPDH
shNC shLARP4B shLARP4B+SPINK1
310 245 180 140 100 75 60 45
Huh7
CD31
shNC shLARP4B shLARP4B+SPINK1
180 130 100 70 55 40 35 25
MHCC-97H
CD34
shNC shLARP4B shLARP4B+SPINK1
130 100 70 55 40 35
MHCC-97H
GAPDH
shNC shLARP4B shLARP4B+SPINK1
310 245 180 140 100 75 60 45
MHCC-97H

P

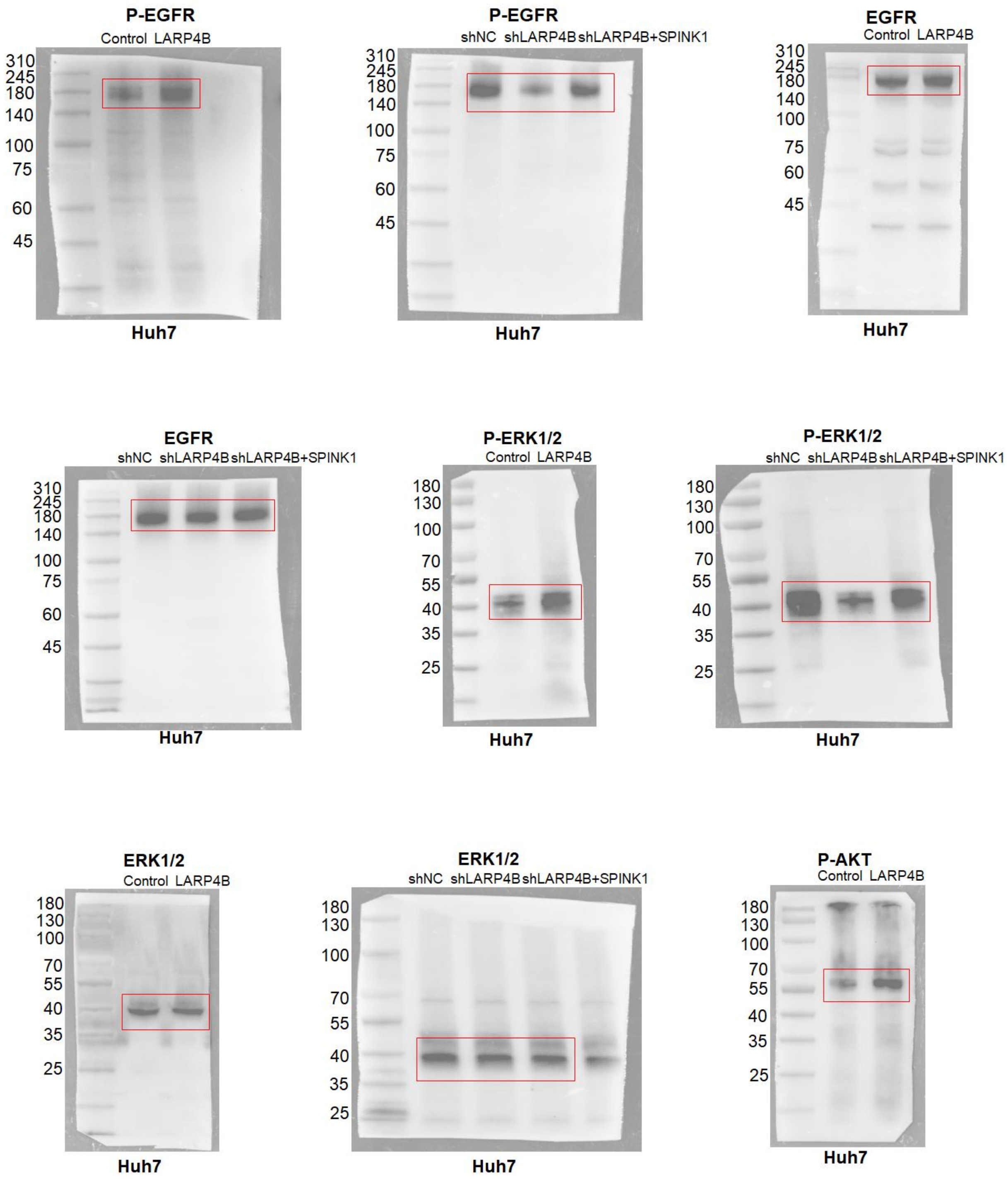

P-EGFR
Control LARP4B
310
245
180
140
100
75
60
45
Huh7
P-EGFR
shNC shLARP4B shLARP4B+SPINK1
310
245
180
140
100
75
60
45
Huh7
EGFR
Control LARP4B
310
245
180
140
100
75
60
45
Huh7
EGFR
shNC shLARP4B shLARP4B+SPINK1
310
245
180
140
100
75
60
45
Huh7
P-ERK1/2
Control LARP4B
180
130
100
70
55
40
35
25
Huh7
P-ERK1/2
shNC shLARP4B shLARP4B+SPINK1
180
130
100
70
55
40
35
25
Huh7
ERK1/2
Control LARP4B
180
130
100
70
55
40
35
25
Huh7
ERK1/2
shNC shLARP4B shLARP4B+SPINK1
180
130
100
70
55
40
35
25
Huh7
P-AKT
Control LARP4B
180
130
100
70
55
40
35
25
Huh7

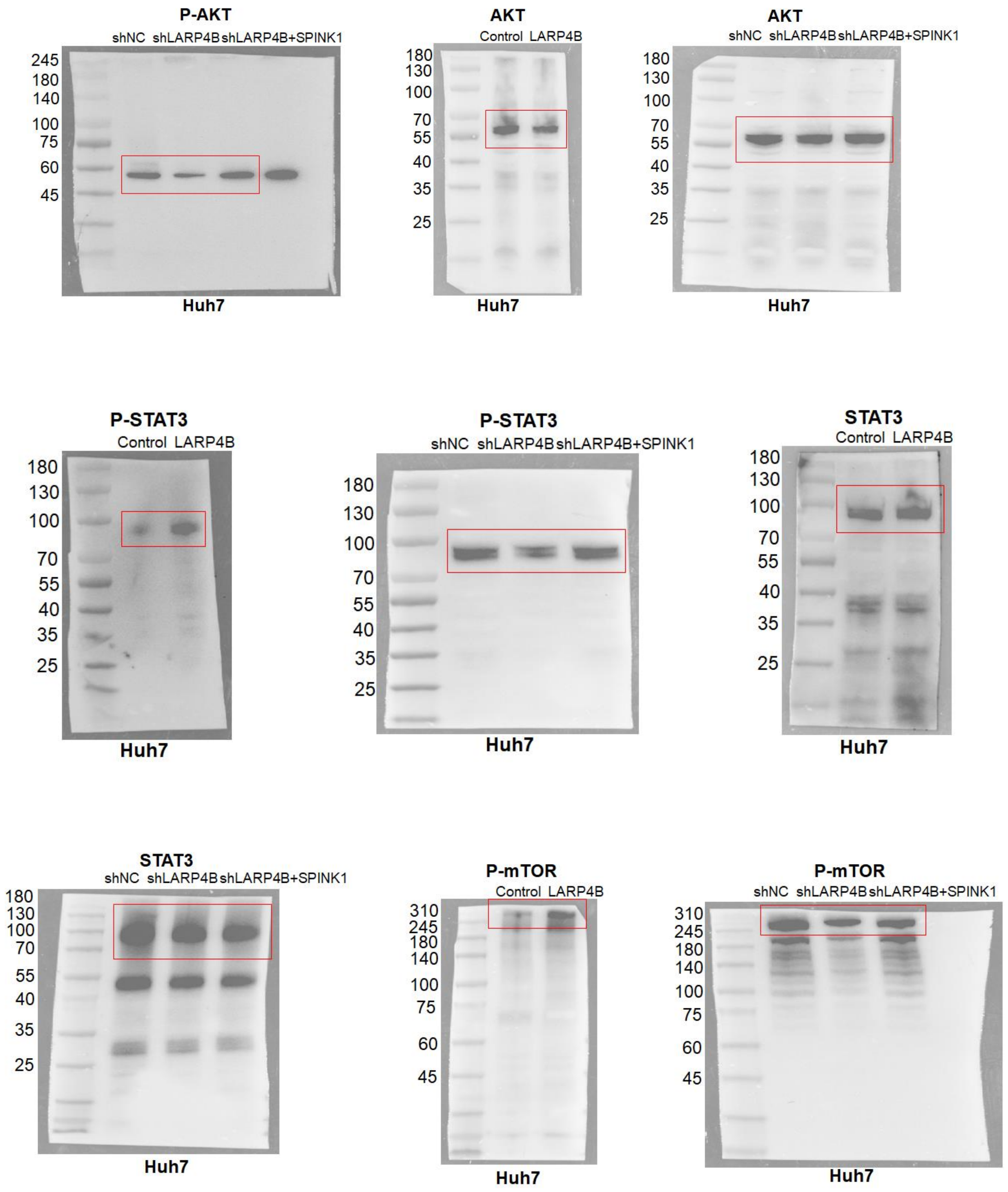
P-AKT
shNC shLARP4B shLARP4B+SPINK1
245 180 140 100 75 60 45
Huh7
AKT
Control LARP4B
180 130 100 70 55 40 35 25
Huh7
AKT
shNC shLARP4B shLARP4B+SPINK1
180 130 100 70 55 40 35 25
Huh7
P-STAT3
Control LARP4B
180 130 100 70 55 40 35 25
Huh7
P-STAT3
shNC shLARP4B shLARP4B+SPINK1
180 130 100 70 55 40 35 25
Huh7
STAT3
Control LARP4B
180 130 100 70 55 40 35 25
Huh7
STAT3
shNC shLARP4B shLARP4B+SPINK1
180 130 100 70 55 40 35 25
Huh7
P-mTOR
Control LARP4B
310 245 180 140 100 75 60 45
Huh7
P-mTOR
shNC shLARP4B shLARP4B+SPINK1
310 245 180 140 100 75 60 45
Huh7

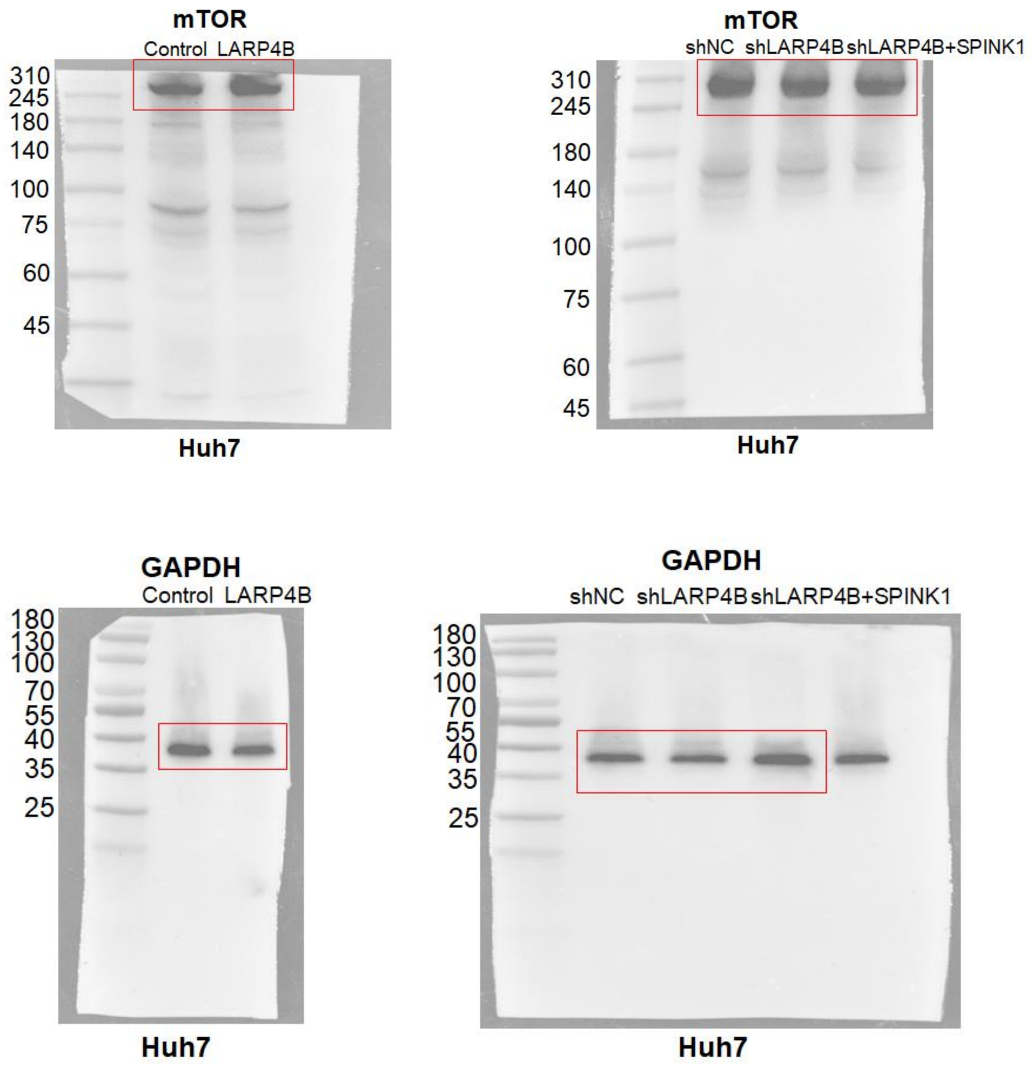
mTOR
Control LARP4B
310
245
180
140
100
75
60
45
Huh7
mTOR
shNC shLARP4B shLARP4B+SPINK1
310
245
180
140
100
75
60
45
Huh7
GAPDH
Control LARP4B
180
130
100
70
55
40
35
25
Huh7
GAPDH
shNC shLARP4B shLARP4B+SPINK1
180
130
100
70
55
40
35
25
Huh7

Fig. 8

A

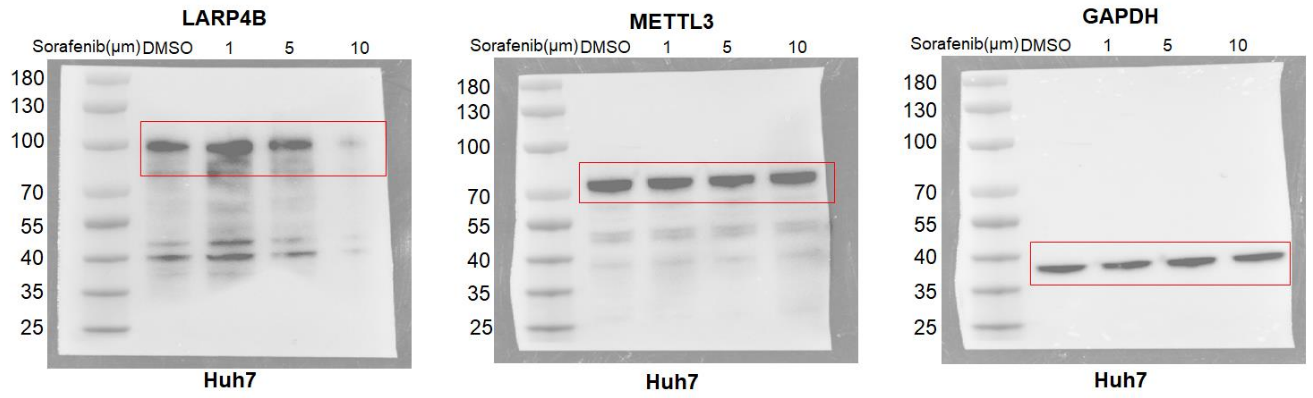
LARP4B
Sorafenib(μm) DMSO 1 5 10
180
130
100
70
55
40
35
25
Huh7
METTL3
Sorafenib(μm) DMSO 1 5 10
180
130
100
70
55
40
35
25
Huh7
GAPDH
Sorafenib(μm) DMSO 1 5 10
180
130
100
70
55
40
35
25
Huh7

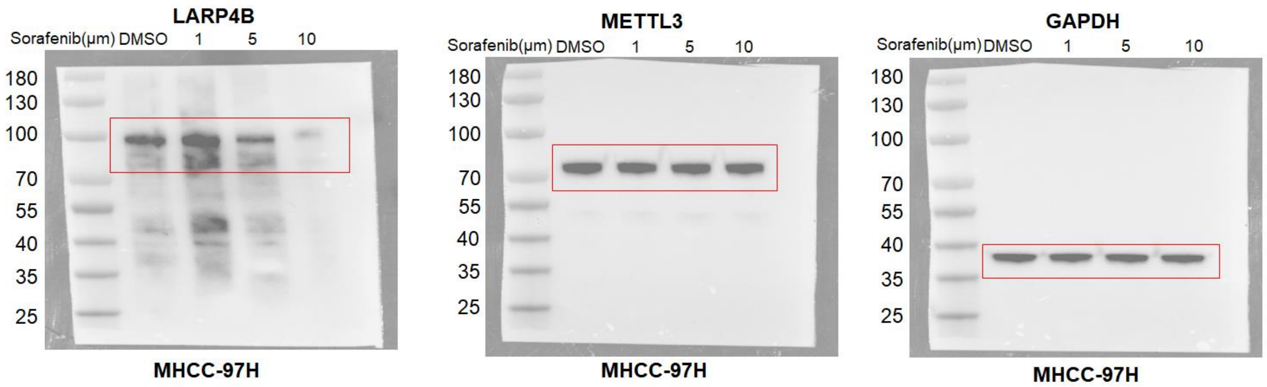
LARP4B
Sorafenib(μm) DMSO 1 5 10
180
130
100
70
55
40
35
25
MHCC-97H
METTL3
Sorafenib(μm) DMSO 1 5 10
180
130
100
70
55
40
35
25
MHCC-97H
GAPDH
Sorafenib(μm) DMSO 1 5 10
180
130
100
70
55
40
35
25
MHCC-97H

B

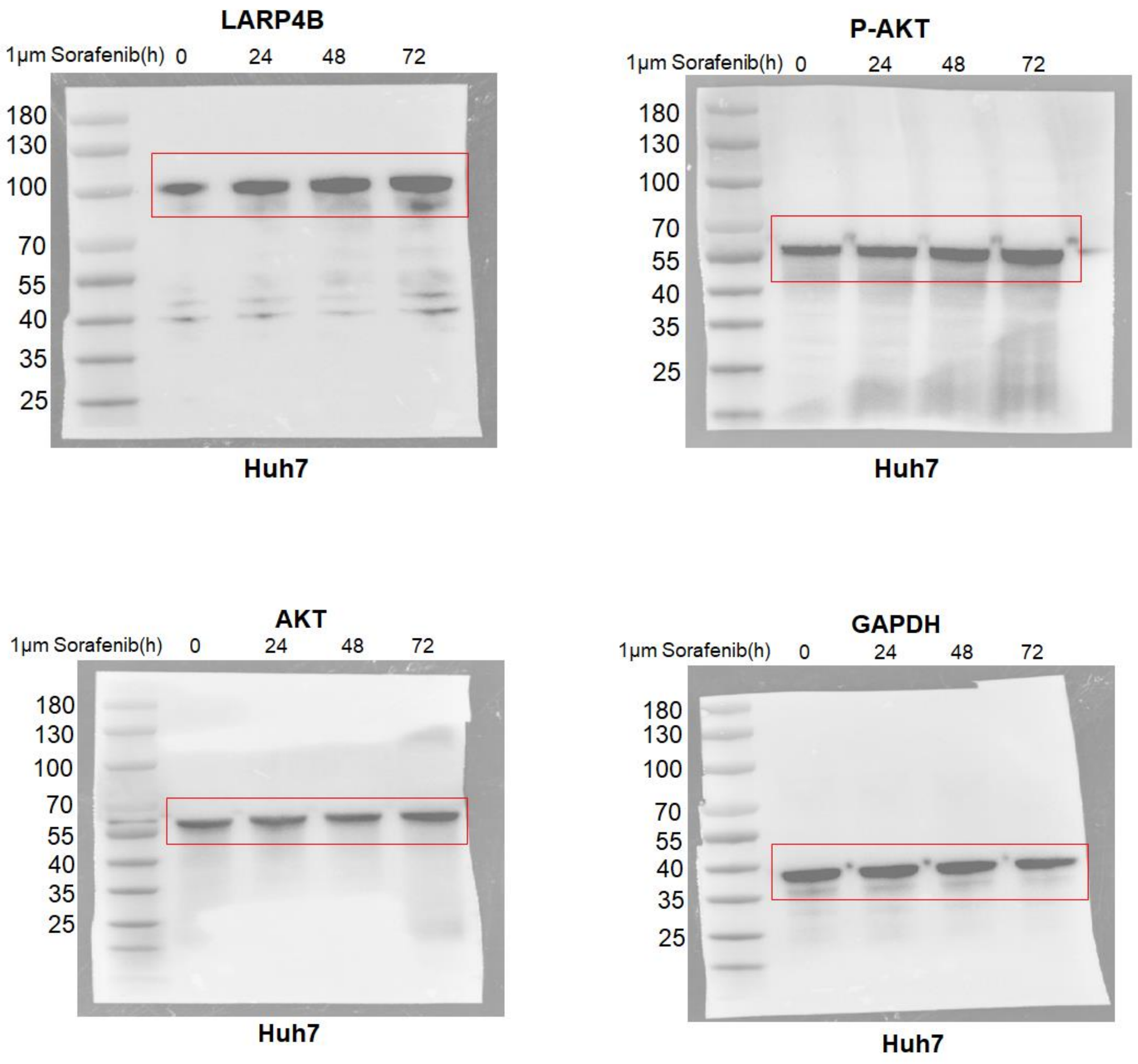
LARP4B
1μm Sorafenib(h) 0 24 48 72
180
130
100
70
55
40
35
25
Huh7
P-AKT
1μm Sorafenib(h) 0 24 48 72
180
130
100
70
55
40
35
25
Huh7
AKT
1μm Sorafenib(h) 0 24 48 72
180
130
100
70
55
40
35
25
Huh7
GAPDH
1μm Sorafenib(h) 0 24 48 72
180
130
100
70
55
40
35
25
Huh7

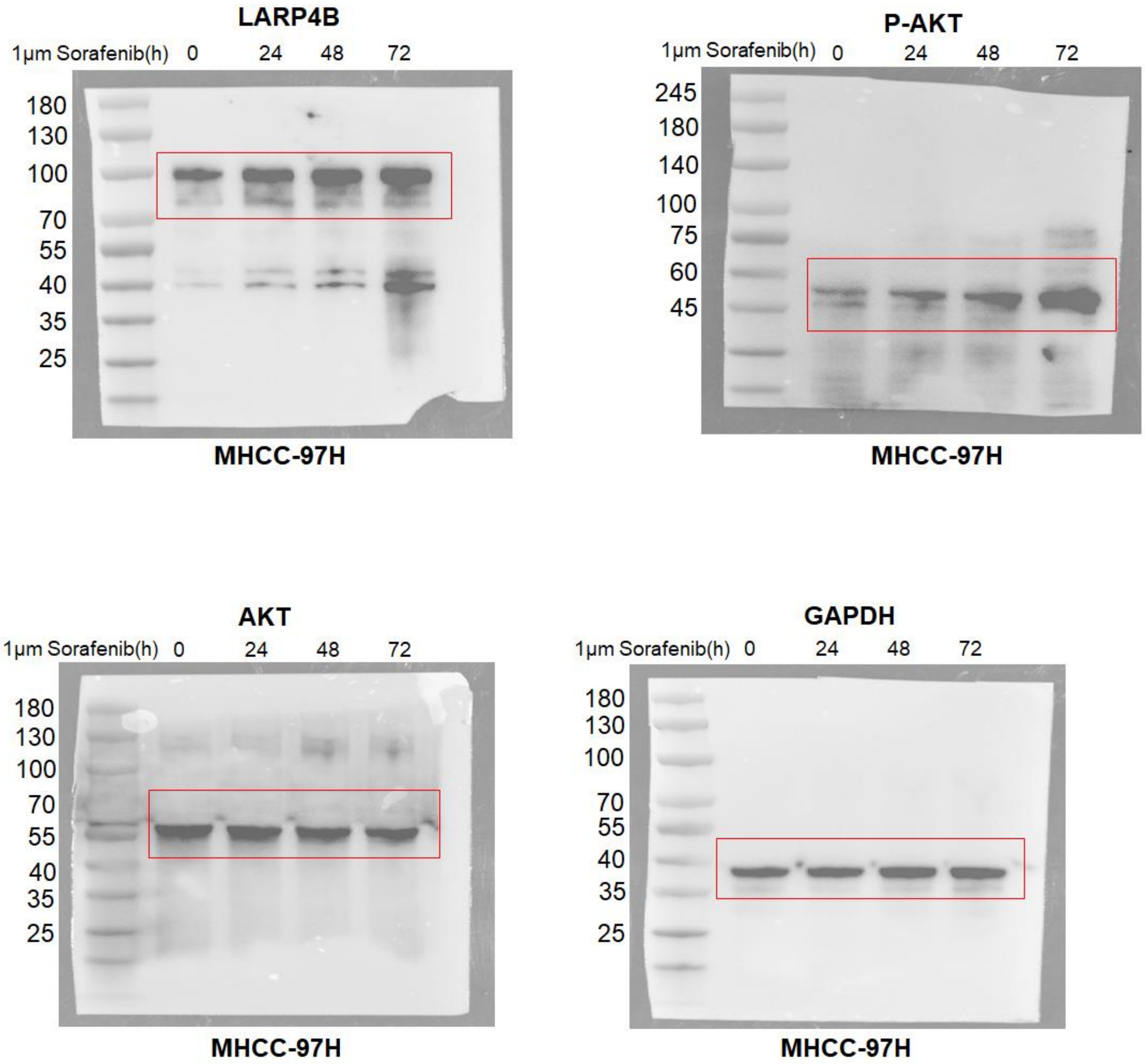
LARP4B
1μm Sorafenib(h) 0 24 48 72
180
130
100
70
55
40
35
25
MHCC-97H
P-AKT
1μm Sorafenib(h) 0 24 48 72
245
180
140
100
75
60
45
MHCC-97H
AKT
1μm Sorafenib(h) 0 24 48 72
180
130
100
70
55
40
35
25
MHCC-97H
GAPDH
1μm Sorafenib(h) 0 24 48 72
180
130
100
70
55
40
35
25
MHCC-97H

D

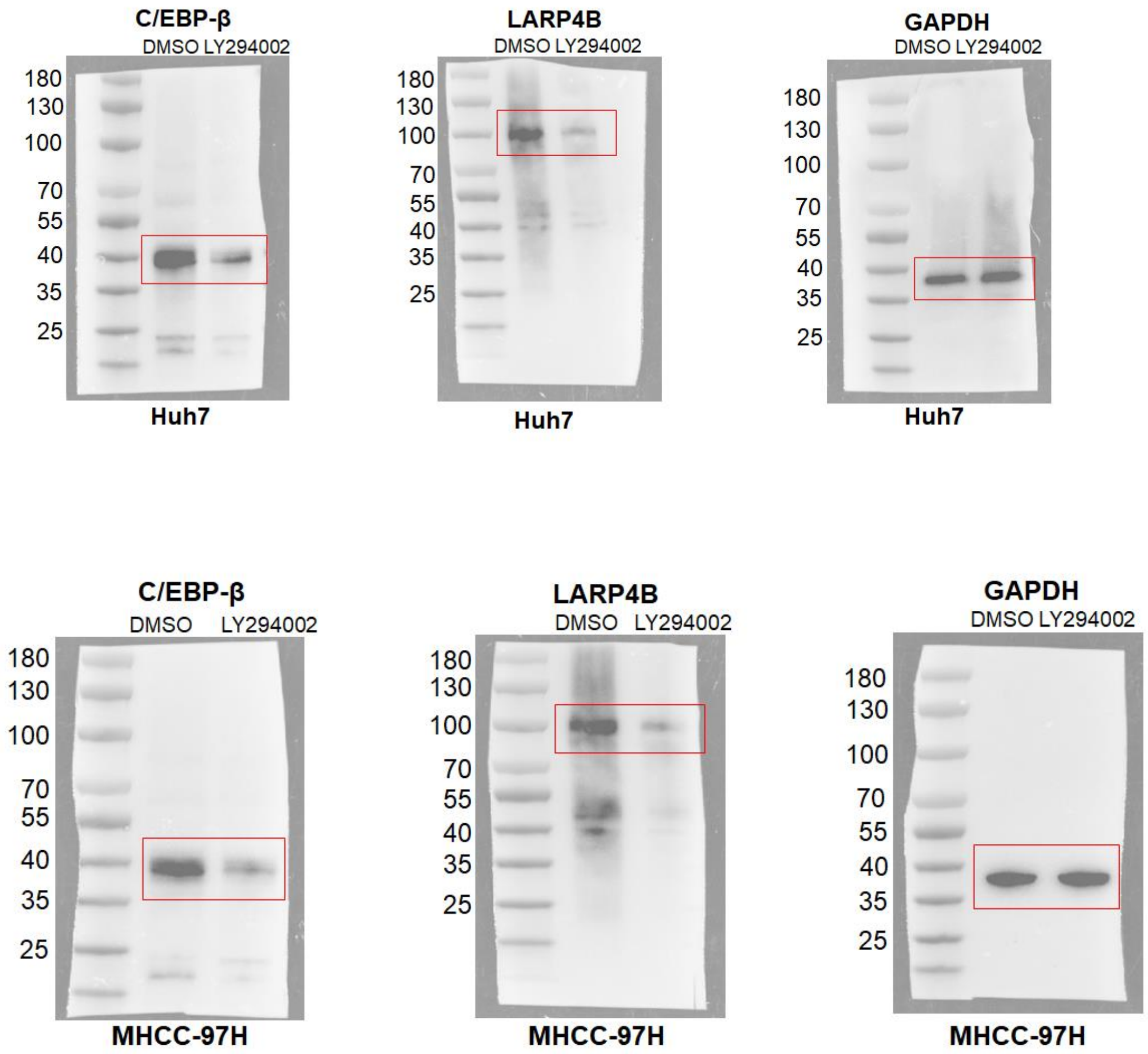
C/EBP-β
DMSO LY294002
180
130
100
70
55
40
35
25
Huh7
LARP4B
DMSO LY294002
180
130
100
70
55
40
35
25
Huh7
GAPDH
DMSO LY294002
180
130
100
70
55
40
35
25
Huh7
C/EBP-β
DMSO LY294002
180
130
100
70
55
40
35
25
MHCC-97H
LARP4B
DMSO LY294002
180
130
100
70
55
40
35
25
MHCC-97H
GAPDH
DMSO LY294002
180
130
100
70
55
40
35
25
MHCC-97H

E

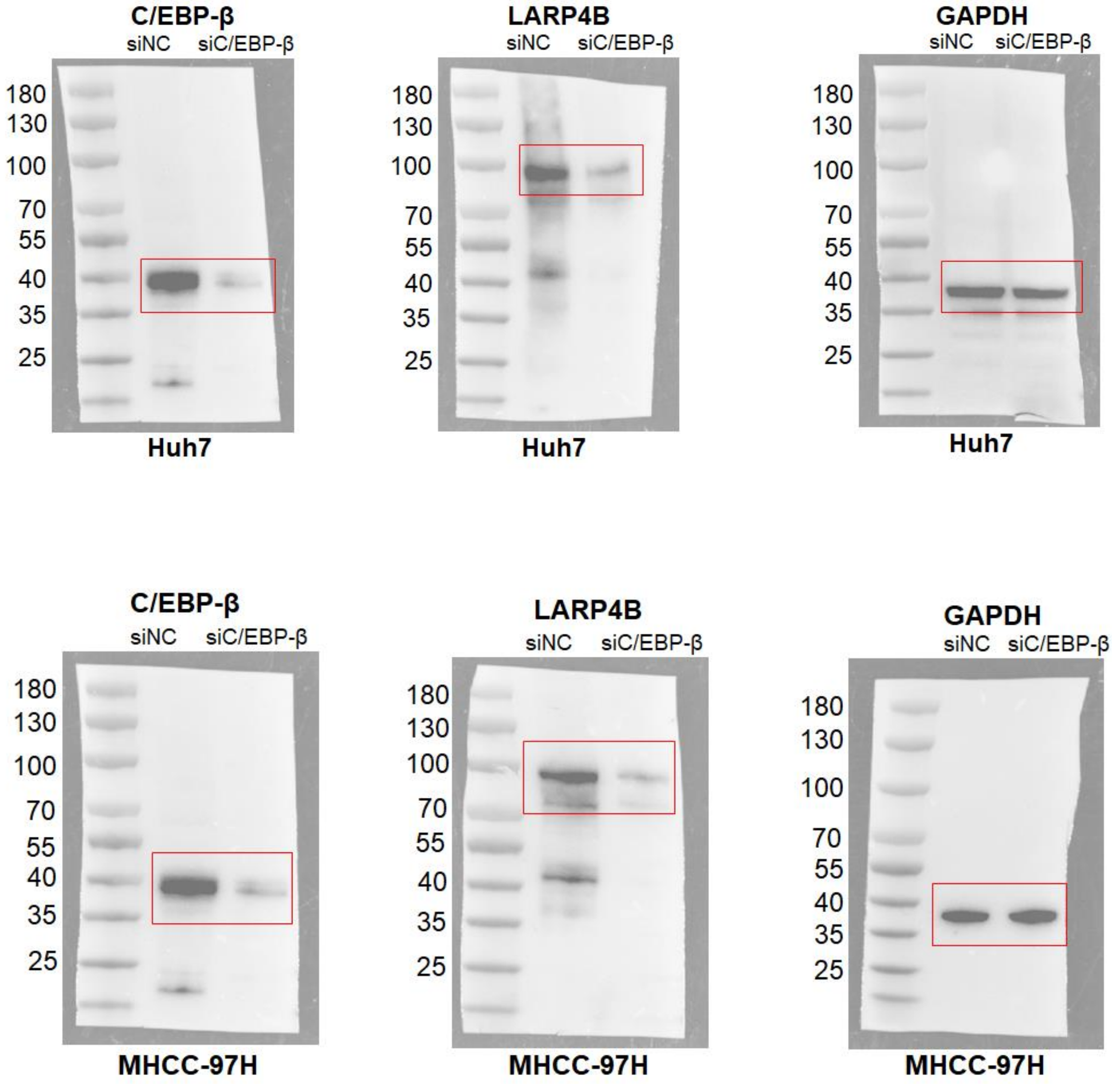
C/EBP-β
siNC
siC/EBP-β
180
130
100
70
55
40
35
25
Huh7
LARP4B
siNC
siC/EBP-β
180
130
100
70
55
40
35
25
Huh7
GAPDH
siNC
siC/EBP-β
180
130
100
70
55
40
35
25
Huh7
C/EBP-β
siNC
siC/EBP-β
180
130
100
70
55
40
35
25
MHCC-97H
LARP4B
siNC
siC/EBP-β
180
130
100
70
55
40
35
25
MHCC-97H
GAPDH
siNC
siC/EBP-β
180
130
100
70
55
40
35
25
MHCC-97H

ChIP

Fig. 9

F

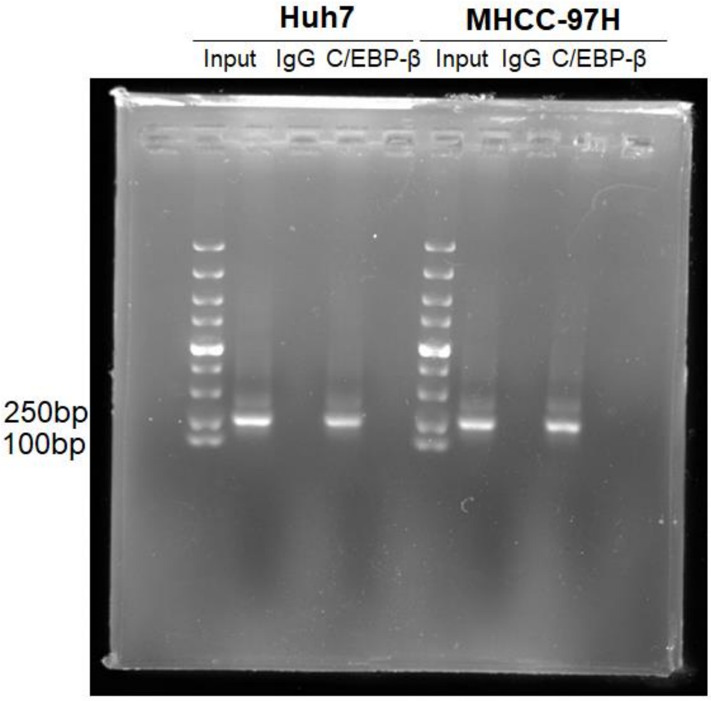

Supplementary Fig. S2

A

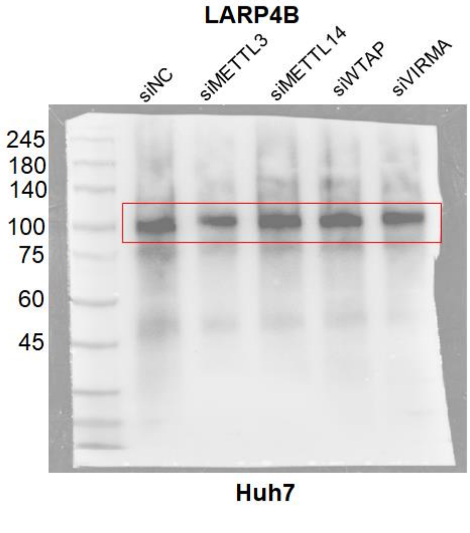

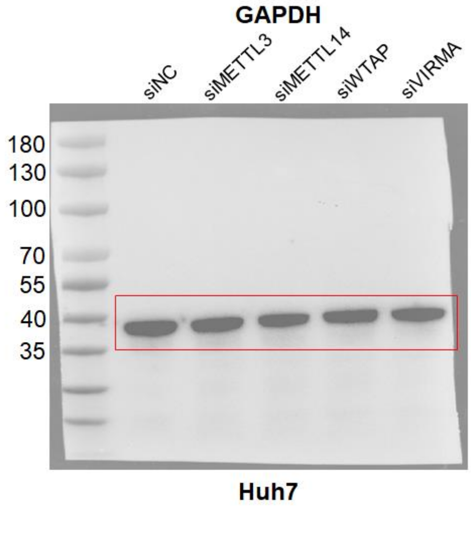

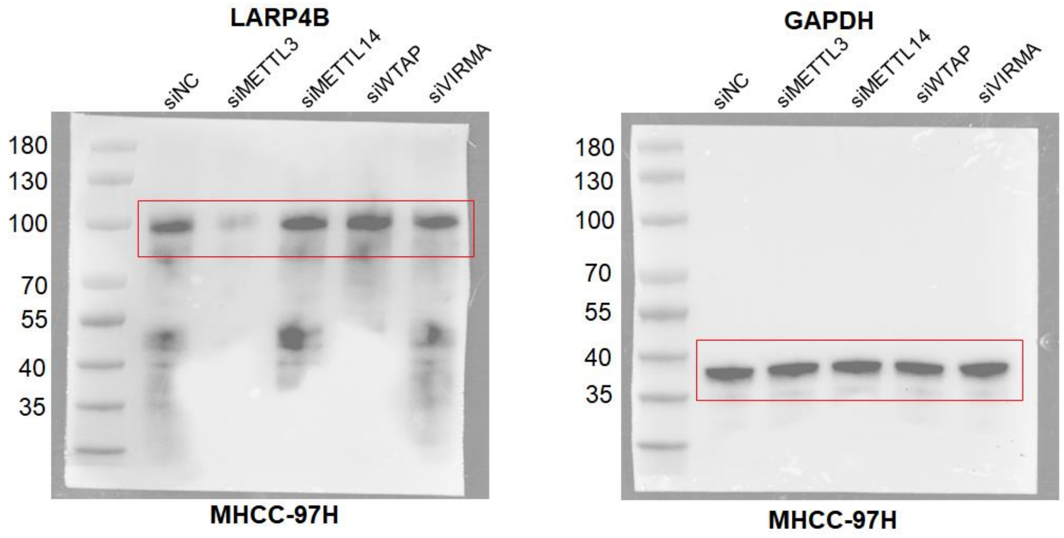
LARP4B
siNC
siMETTL3
siMETTL14
siWTAP
siVIRMA
180
130
100
70
55
40
35
MHCC-97H
GAPDH
siNC
siMETTL3
siMETTL14
siWTAP
siVIRMA
180
130
100
70
55
40
35
MHCC-97H

B

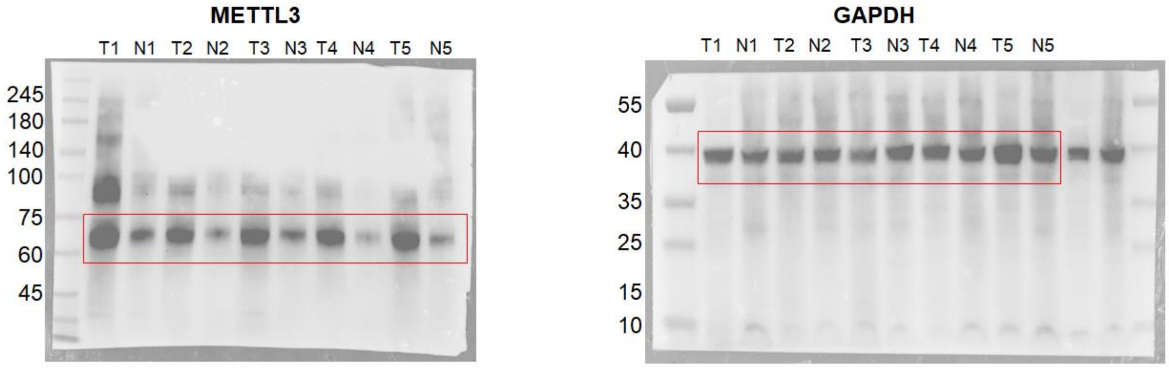
METTL3
T1 N1 T2 N2 T3 N3 T4 N4 T5 N5
245
180
140
100
75
60
45
GAPDH
T1 N1 T2 N2 T3 N3 T4 N4 T5 N5
55
40
35
25
15
10

D

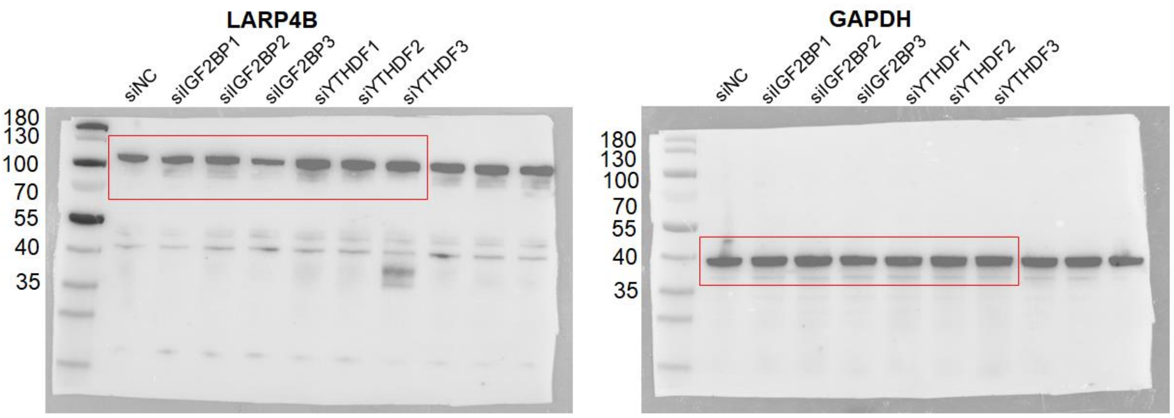
LARP4B
siNC
siIGF2BP1
siIGF2BP2
siIGF2BP3
siYTHDF1
siYTHDF2
siYTHDF3
180
130
100
70
55
40
35
GAPDH
siNC
siIGF2BP1
siIGF2BP2
siIGF2BP3
siYTHDF1
siYTHDF2
siYTHDF3
180
130
100
70
55
40
35

Supplementary Fig. S3

A

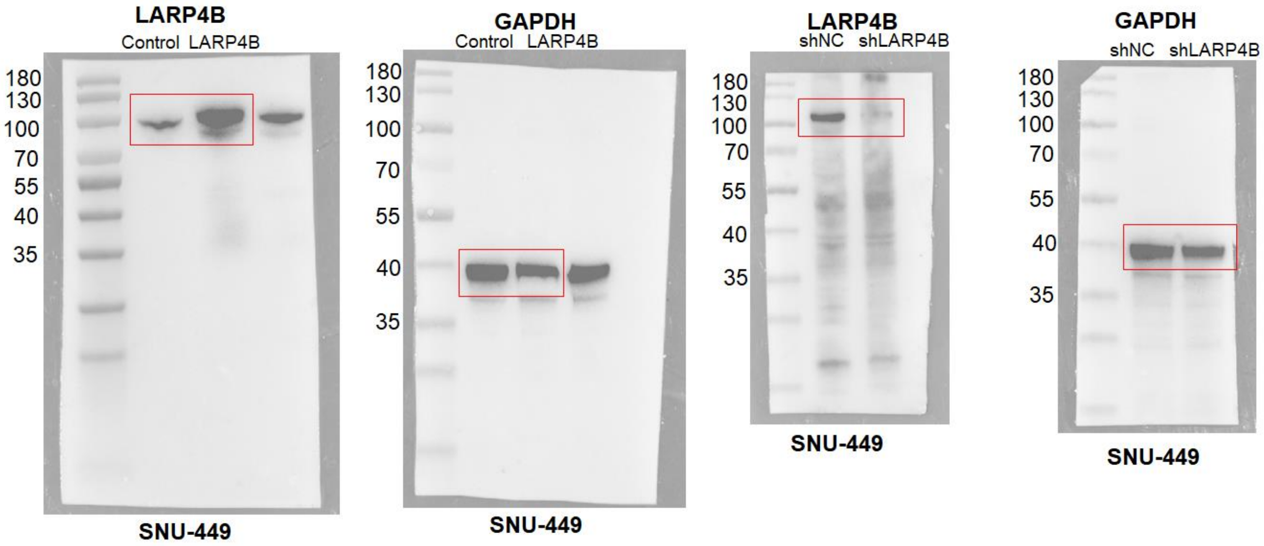

Supplementary Fig. S5

D

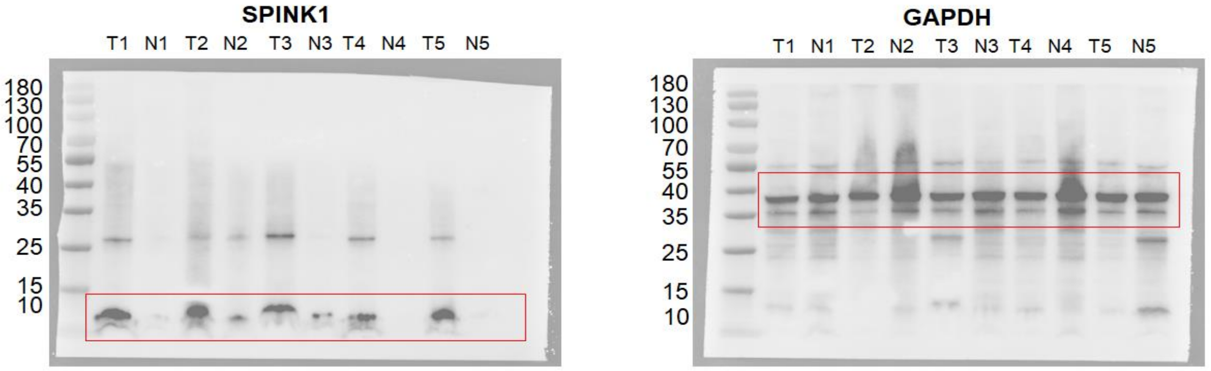

Supplementary Fig. S6

A

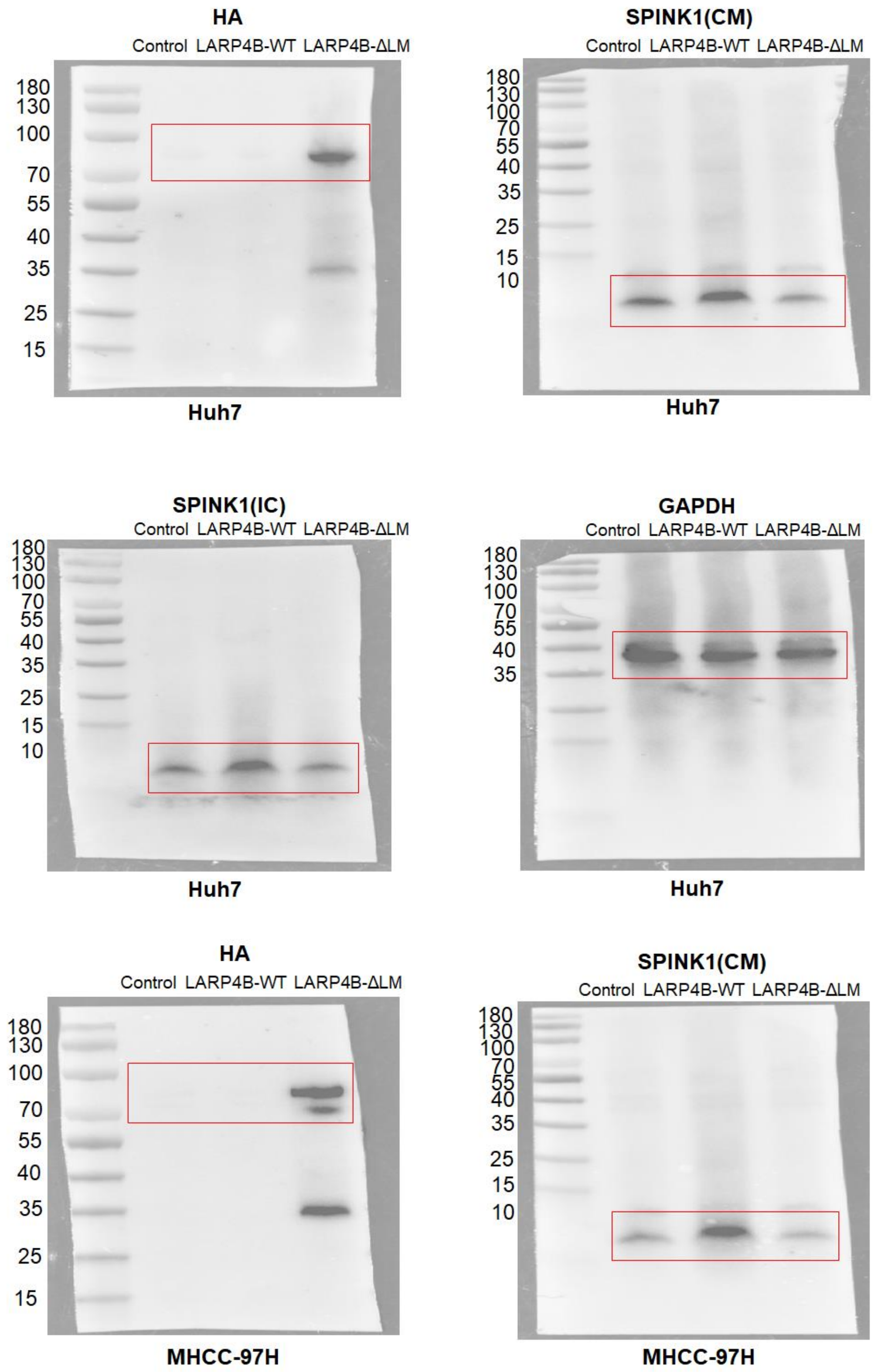

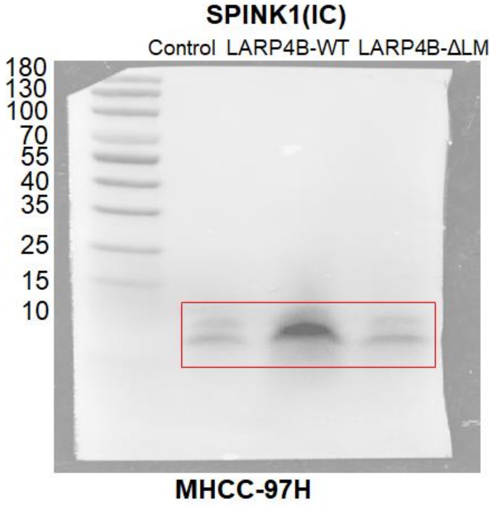

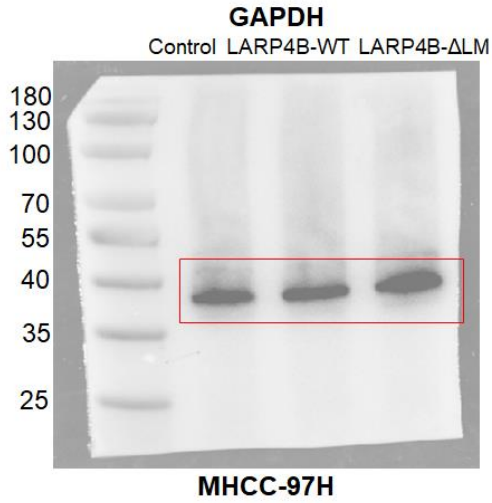

Supplementary Fig. S7

D

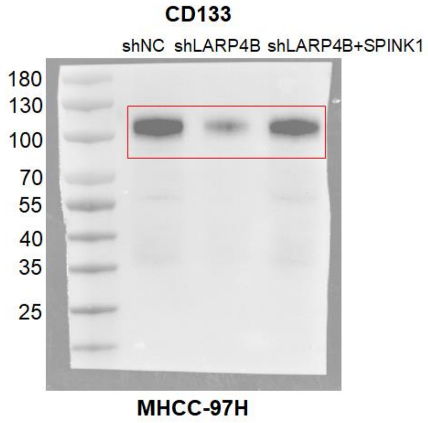

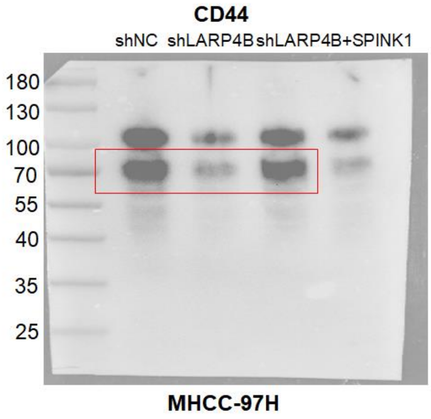

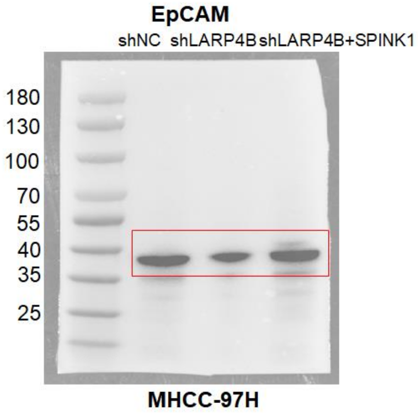

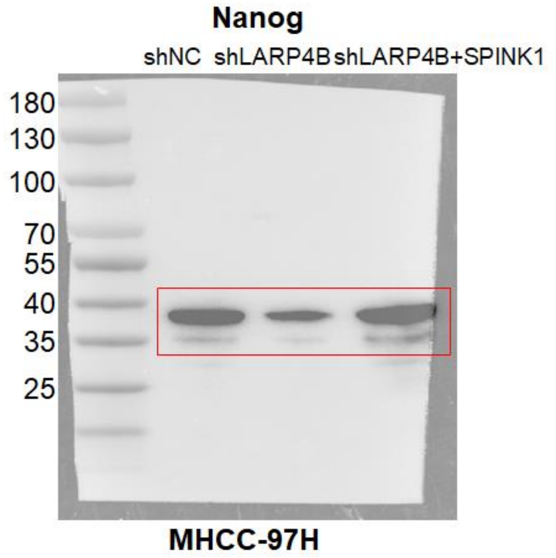
Nanog
shNC shLARP4B shLARP4B+SPINK1
180
130
100
70
55
40
35
25
MHCC-97H
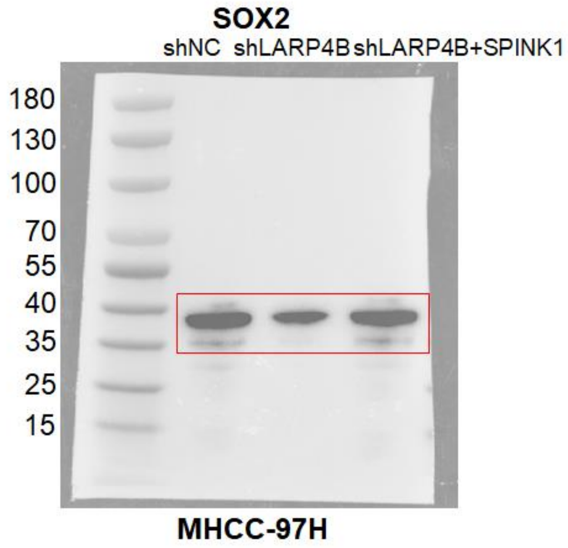
SOX2
shNC shLARP4B shLARP4B+SPINK1
180
130
100
70
55
40
35
25
15
MHCC-97H
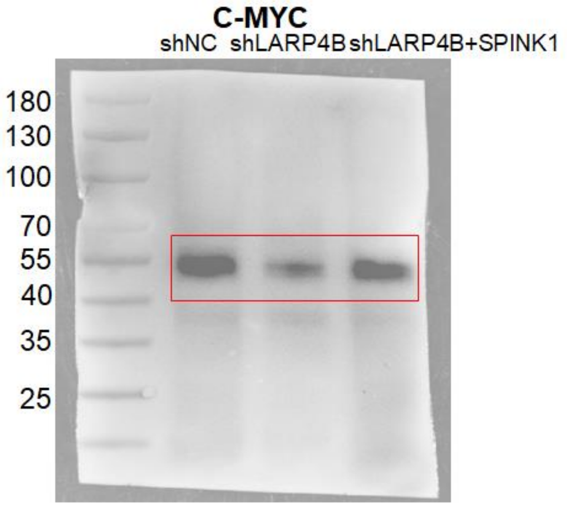
C-MYC
shNC shLARP4B shLARP4B+SPINK1
180
130
100
70
55
40
35
25
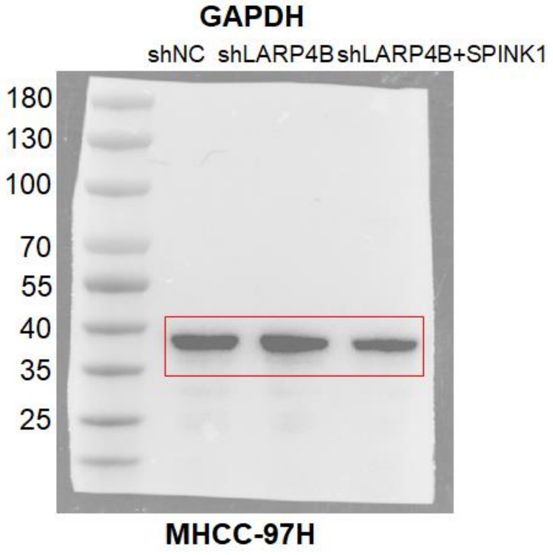
GAPDH
shNC shLARP4B shLARP4B+SPINK1
180
130
100
70
55
40
35
25
MHCC-97H

J

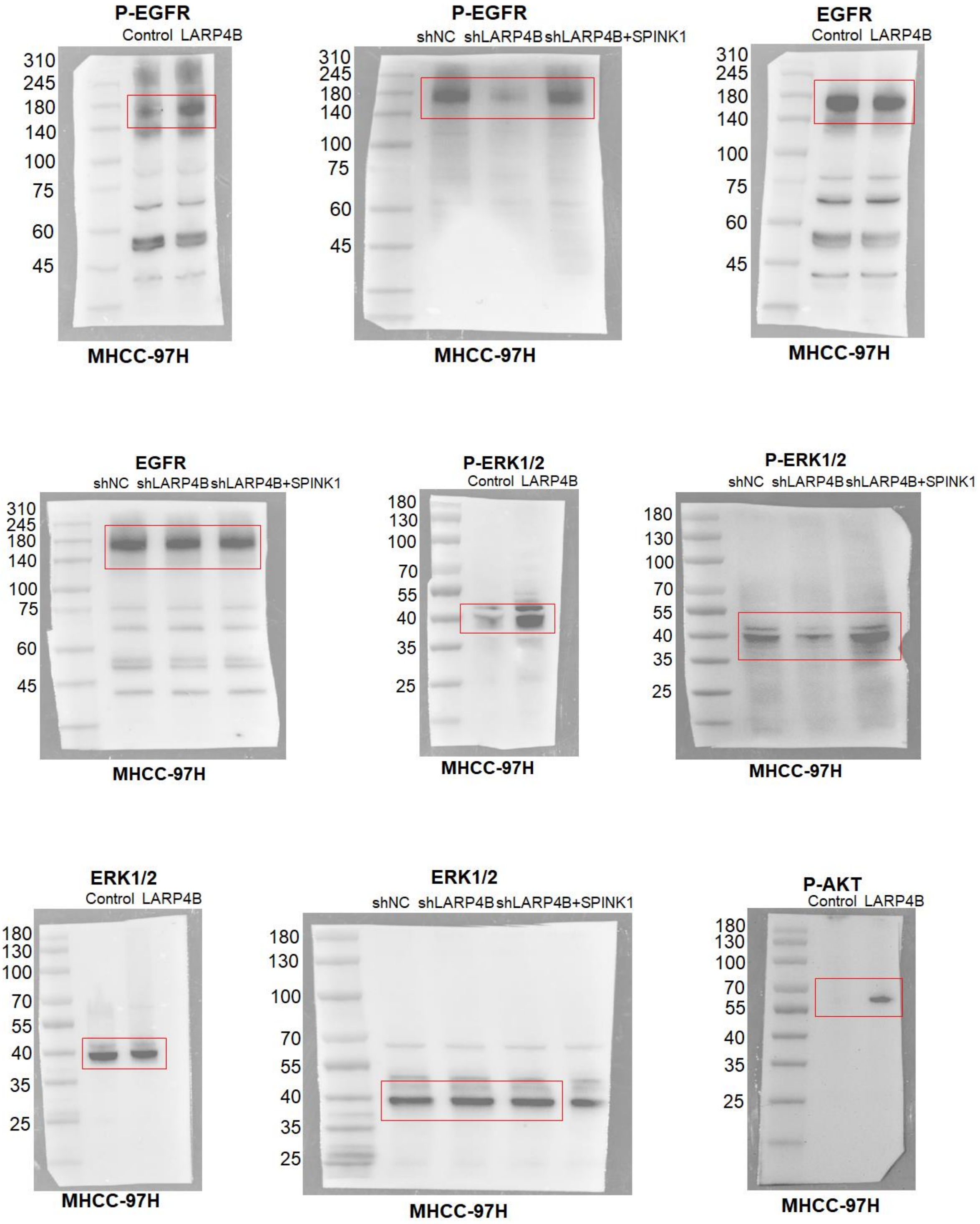
P-EGFR
Control LARP4B
310
245
180
140
100
75
60
45
MHCC-97H
P-EGFR
shNC shLARP4B shLARP4B+SPINK1
310
245
180
140
100
75
60
45
MHCC-97H
EGFR
Control LARP4B
310
245
180
140
100
75
60
45
MHCC-97H
EGFR
shNC shLARP4B shLARP4B+SPINK1
310
245
180
140
100
75
60
45
MHCC-97H
P-ERK1/2
Control LARP4B
180
130
100
70
55
40
35
25
MHCC-97H
P-ERK1/2
shNC shLARP4B shLARP4B+SPINK1
180
130
100
70
55
40
35
25
MHCC-97H
ERK1/2
Control LARP4B
180
130
100
70
55
40
35
25
MHCC-97H
ERK1/2
shNC shLARP4B shLARP4B+SPINK1
180
130
100
70
55
40
35
25
MHCC-97H
P-AKT
Control LARP4B
180
130
100
70
55
40
35
25
MHCC-97H

P-AKT

shNC shLARP4B shLARP4B+SPINK1

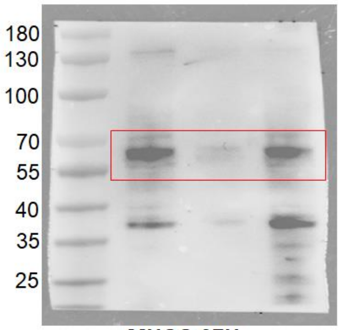

MHCC-97H

AKT

Control LARP4B

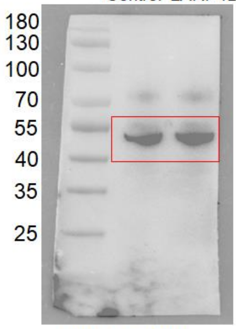

MHCC-97H

AKT

shNC shLARP4B shLARP4B+SPINK1

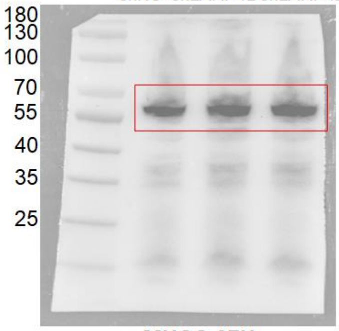

MHCC-97H

P-STAT3

Control LARP4B

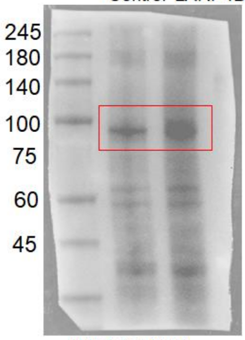

MHCC-97H

P-STAT3

shNC shLARP4B shLARP4B+SPINK1

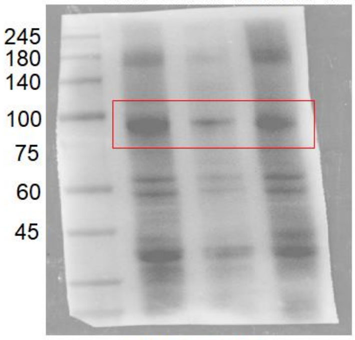

MHCC-97H

STAT3

Control LARP4B

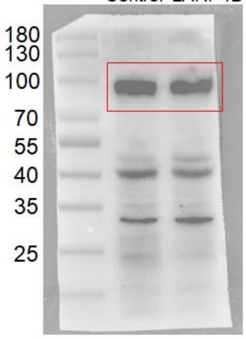

MHCC-97H

STAT3

shNC shLARP4B shLARP4B+SPINK1

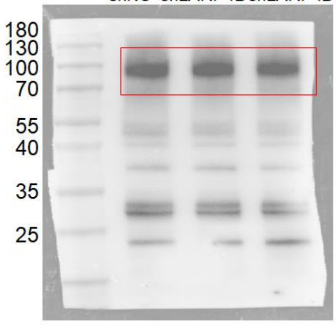

MHCC-97H

P-mTOR

Control LARP4B

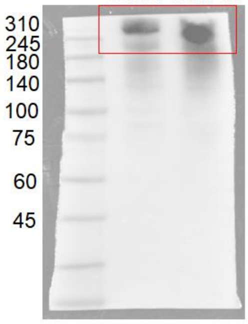

MHCC-97H

P-mTOR

shNC shLARP4B shLARP4B+SPINK1

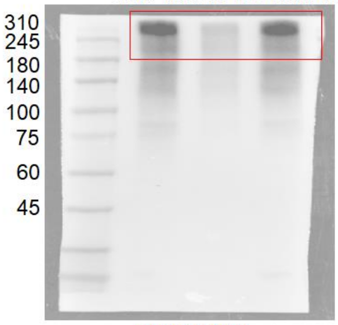

MHCC-97H

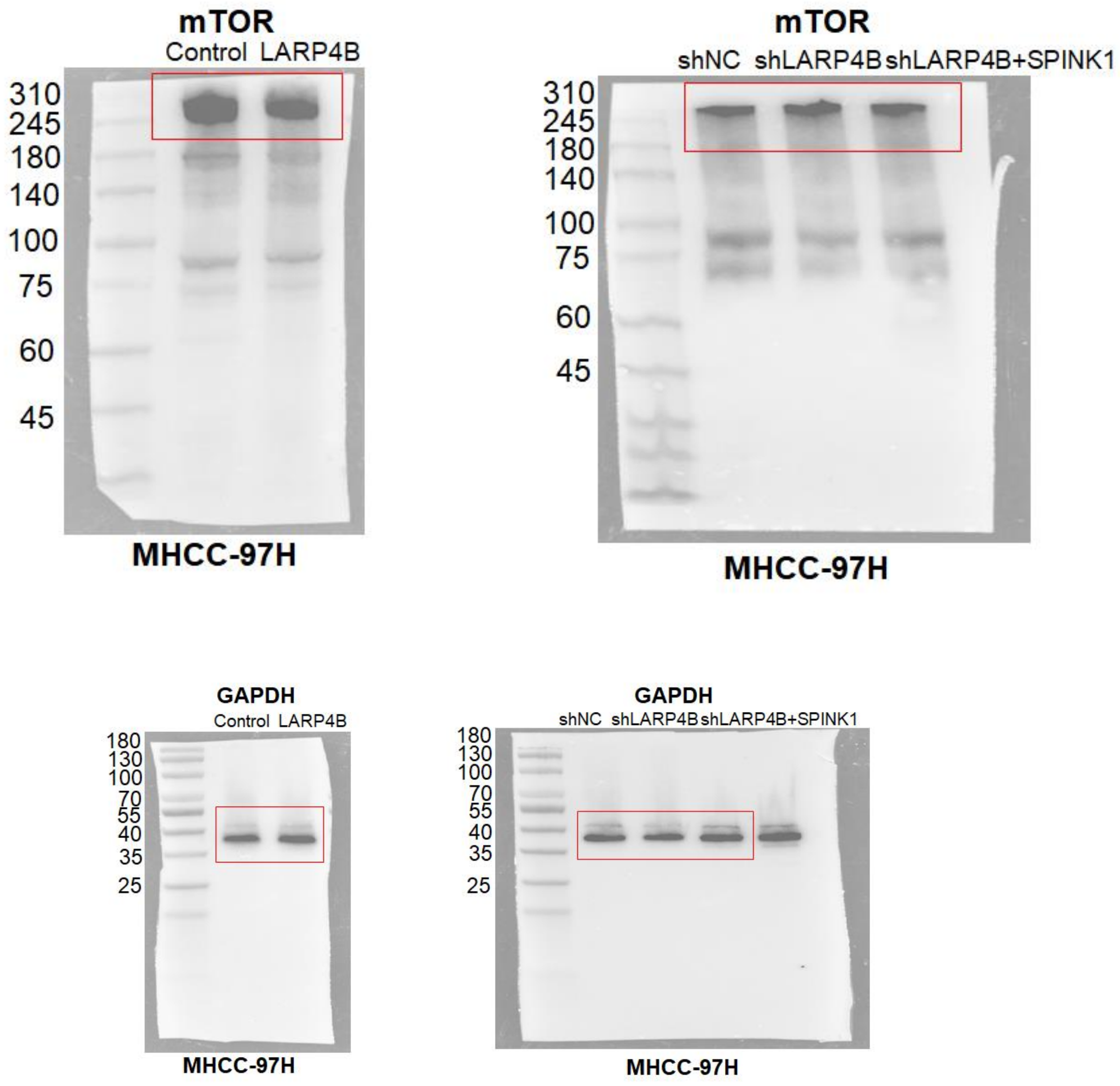

Supplementary Fig. S8

A

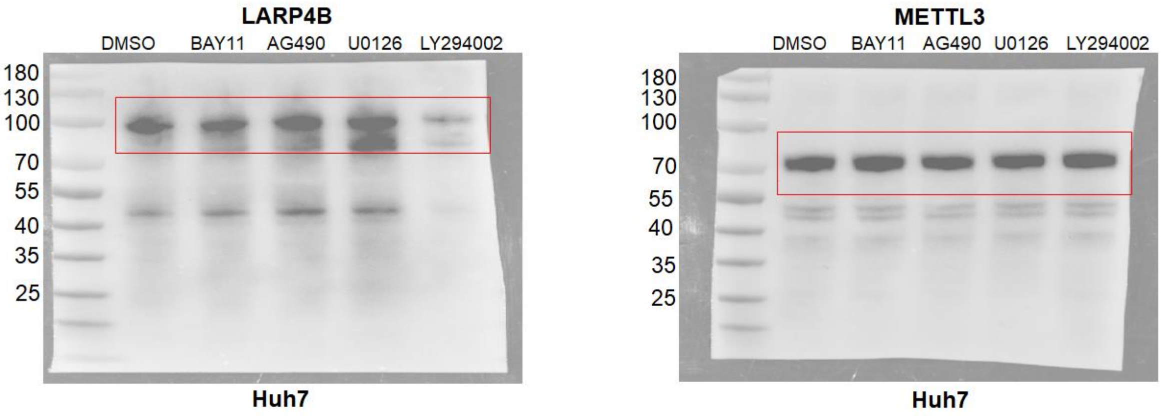

GAPDH

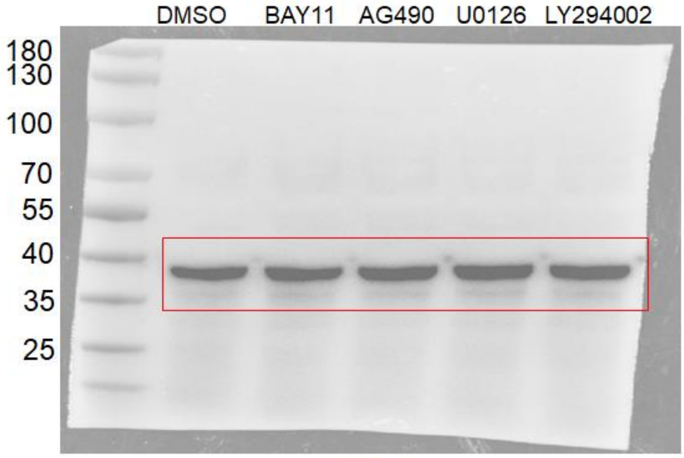

Huh7

LARP4B

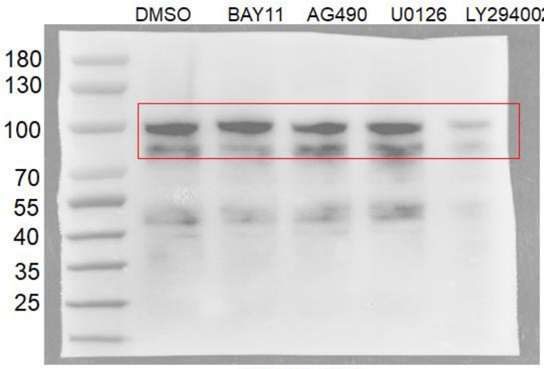

MHCC-97H

METTL3

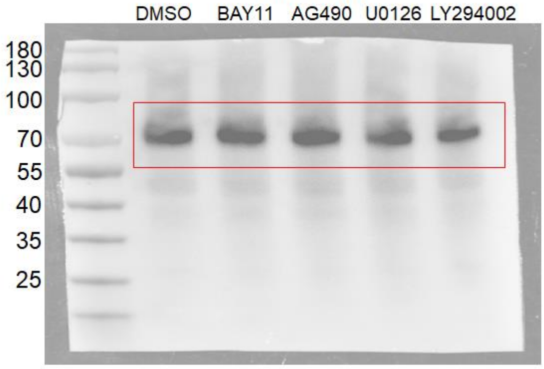

MHCC-97H

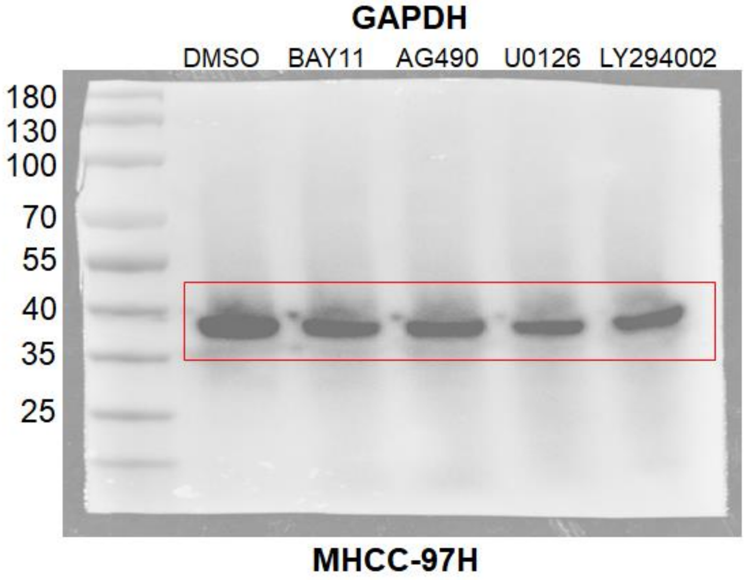
GAPDH
DMSO BAY11 AG490 U0126 LY294002
180
130
100
70
55
40
35
25
MHCC-97H

B

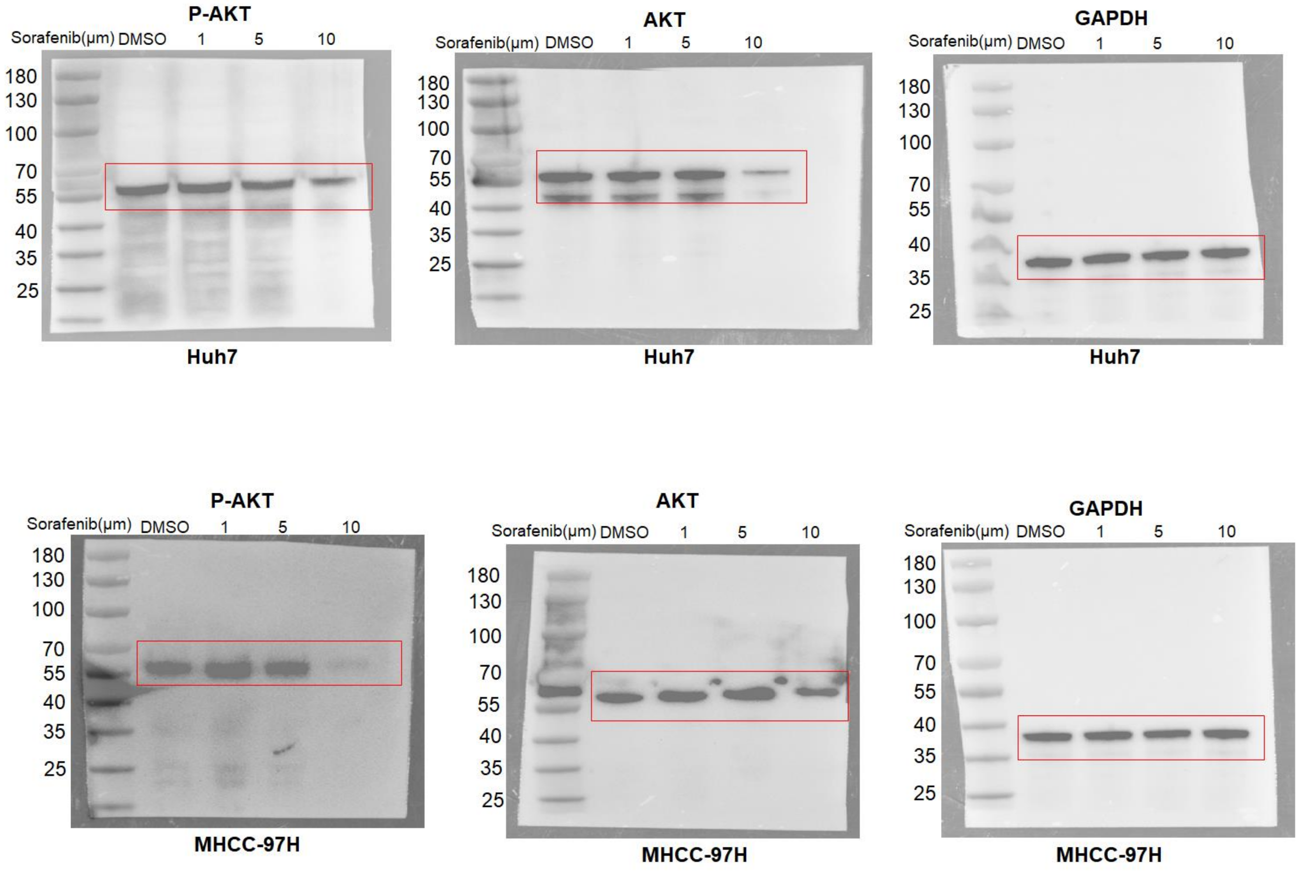
P-AKT
Sorafenib(μm) DMSO 1 5 10
180
130
100
70
55
40
35
25
Huh7
AKT
Sorafenib(μm) DMSO 1 5 10
180
130
100
70
55
40
35
25
Huh7
GAPDH
Sorafenib(μm) DMSO 1 5 10
180
130
100
70
55
40
35
25
Huh7
P-AKT
Sorafenib(μm) DMSO 1 5 10
180
130
100
70
55
40
35
25
MHCC-97H
AKT
Sorafenib(μm) DMSO 1 5 10
180
130
100
70
55
40
35
25
MHCC-97H
GAPDH
Sorafenib(μm) DMSO 1 5 10
180
130
100
70
55
40
35
25
MHCC-97H
